# Author Correction: A modular and synthetic biosynthesis platform for de novo production of diverse halogenated tryptophan-derived molecules

**DOI:** 10.1038/s41467-024-49314-w

**Published:** 2024-06-07

**Authors:** Kevin B. Reed, Sierra M. Brooks, Jordan Wells, Kristin J. Blake, Minye Zhao, Kira Placido, Simon d’Oelsnitz, Adit Trivedi, Shruti Gadhiyar, Hal S. Alper

**Affiliations:** 1 McKetta Department of Chemical Engineering, The University of Texas at Austin, 200 E Dean Keeton St. Stop C0400, Austin, TX USA; 2 Mass Spectrometry Facility, Department of Chemistry, The University of Texas at Austin, 105 E 24th Street, Austin, TX USA; 3 Institute for Cellular and Molecular Biology, The University of Texas at Austin, 2500 Speedway Avenue, Austin, TX USA

**Keywords:** Biocatalysis, Metabolic engineering, Applied microbiology, Metabolic pathways

Correction to: *Nature Communications* 10.1038/s41467-024-47387-1, published online 12 April 2024

The original version of this article and the Supplementary Information file contained an error in figures 4D, 5A, 6A, 7, and 8, as well as Supplementary Figs. 18–22, 47, and 68–72. In the original version of these figures and Supplementary Figures, the formyl group was incorrectly connected to the alpha nitrogen of the amino group for N-formyl-L-kynurenine and the derived compounds. The correct connection of the formyl group is the indolic nitrogen clśose to the aromatic ring. These have been corrected in both the PDF and HTML versions of the article, and the HTML version of the Supplementary Information.

### Supplementary information

Updated Supplementary Information

