# Supplementary material for: A modular and synthetic biosynthesis platform for de novo production of diverse halogenated tryptophan-derived molecules
Source: Nat Commun. 2024 Apr 12;15:3188. doi: 10.1038/s41467-024-47387-1 (PMC11015028; doi:10.1038/s41467-024-47387-1)
Supplement: Supplementary file 1 — Supplementary Information [file 41467_2024_47387_MOESM1_ESM.pdf]

A modular and synthetic biosynthesis platform for *de novo* production of diverse halogenated tryptophan-derived molecules

Reed and Brooks *et al.*

Supplementary Note 1. Development of optimized halogenase expression strategy

We explored production of halogenated tryptophan with different copy number plasmids and promoters using Th-Hal as a model halogenase. Th-Hal was either expressed under an inducible version of promoter J23101 on plasmid p15A or under an inducible Tac promoter with origin pBR322, denoted as pHal. We discovered that expression on plasmid pHal led to the production of around 1.6-fold as much halogenated tryptophan compared to expression on plasmid p15A, reaching nearly 250 µM after 24 hours. Since *de novo* production from glucose has not been realized for all possible flavors of halogenated tryptophan, we proceeded forward with the best expression strategy developed herein.

Supplementary Discussion 1. Removal of feedback inhibition to bolster tryptophan titer

Removal of feedback inhibition in three particular biosynthesis proteins has been shown to have the largest impact on tryptophan overproduction in a variety of studies[49–51]. These include proteins TrpE, AroG, and SerA, where removal of feedback inhibition is well documented[52]. A linear integration cassette was constructed to enable strong expression of SerA(fbr) and either weak or strong expression of AroG(fbr) to assess the need for general expression optimization. TrpE(fbr) was expressed with a medium strength promoter on a medium copy number plasmid (pTrpOp). In addition, an integration cassette was constructed to replace the TrpL gene locus with a strong, constitutive, unregulated promoter and TrpE(fbr), thus generating a similar overexpression cassette for the trp operon in the genome to compare to the plasmid-borne approach. It was found that the highest levels of tryptophan were generated when AroG(fbr) was strongly expressed and the trp operon was expressed on a plasmid with a medium-strength promoter. Interestingly, additive overexpressions of the tryptophan biosynthesis genes (e.g. expression using both the pTrpOp plasmid and the TrpL::Ptac trp operon cassette) yielded a significant growth deficit, corroborating results from previous tryptophan overproduction endeavors, both empirical and computational[54,55].

Supplementary Discussion 2. Computational investigation confirms enzyme promiscuity trends observed experimentally

Enzymes iaaM and McbB complexed with tryptophan gave binding energies of -139 and -235 REU (Rosetta Energy Units) respectively with the given score function. To determine the relative ability of each enzyme to accommodate the halogenation on tryptophan, we compared the enzyme's ability to bind the halo-trps (ΔΔG of binding) to the enzyme’s ability to bind normal tryptophan (ΔΔG of binding). These two terms were divided, creating a normalized binding potential. This results in a score where 1 represents a binding potential that is near native, 0 represents no binding, and a negative score represents a binding that has a positive ΔΔG. The results are shown in **Supplementary Table 1** with binding structures displayed in **Supplementary Fig. 4-7**. For **Supplemental Figures 5 and 6**, the Tyr216 residue has been labeled for ease of viewing, with the down and up confirmations labeled in pink and blue, respectively. Computationally, iaaM has a greater ability to accommodate halogenated tryptophan substrates in relation to its native substrate when compared to McbB. Additionally, the non-normalized

magnitude of the binding energy for iaaM is also greater than McbB, further supporting this claim. For both enzymes, the binding of different types of halogenated tryptophan, varying in both position and halogen group, were comparable. From these results, we conclude that, computationally, iaaM is more promiscuous than McbB, and thus able to better accommodate the halogen group on tryptophan. This aligns with the experimental results of this study, where McbB was unable to convert any of the 6-chloro- or 6-bromo- tryptophan whereas iaaM could convert all halogenated substrates with very high relative conversion based on HPLC peak heights and LCMS intensities. Interestingly, we do not see a significant drop in binding potential for McbB on the 6-halo- position substrates, which did not turnover experimentally. However, by inspecting the Rosetta docking structures, we can draw a few conclusions about the effect of halogenation on the binding of the tryptophan substrates. For the McbB structures, for both a 5-halo- and 6-halo- substitution, there is a major confirmational change in the Tyr216 due to the introduction of a nearby halogen group, with this residue closest to the 6-position. This is a potential explanation for the experimental data, where the 5-halo- and, especially, 6-halo- substituted substrates show a lesser promiscuity than the 7-halo- substituted substrates. IaaM appears to have a much less compact binding site near the 5, 6, and 7-halo- positions of tryptophan, with no major confirmational changes occurring in the side chains of IaaM with differently substituted tryptophans. This is a possible explanation for the generally increased promiscuity of IaaM when compared to McbB. Differences in binding energy between the various halogenated tryptophan alongs were not observed in the same enzyme. Allowance for major shifts in the position of the substrate or the confirmation of the active site could be attempted in future studies to further elucidate nuanced binding trends. However, certain halogenated positions may have significant effects on the enzyme's reaction mechanism and could thus not be determined through binding studies alone. In all, high level promiscuity trends were corroborated through computational investigation and similar studies could act to narrow down large sets of enzymes to rapidly predict the most promiscuous variants.

Supplementary Table 1. Docking study binding energy scores. Binding energy scores were calculated for both iaaM and McbB complexed with tryptophan (none) and each halo-tryptophan variant that was experimentally tested in this study. Each enzyme's ability to bind the halo-trps (ΔΔG of binding) was compared to the enzyme's ability to bind non-halogenated tryptophan (ΔΔG of binding).

Receptor	Position	Halogen	Binding energy (REU)	Normalized binding energy
iaaM	None	None	-139.0819818	1
	5	Br	-31.63990489	0.229439306
		Cl	-30.05327287	0.22083995
	6	Br	-35.04231958	0.257561137
		Cl	-32.31333541	0.233504574
	7	Br	-29.42593187	0.219063975
		Cl	-28.22415826	0.214507904
McbB	None	None	-234.2799555	1
	5	Br	-26.70300691	0.113979051
		Cl	-27.50496442	0.117402124
	6	Br	-30.52214523	0.130280651
		Cl	-29.31562485	0.125130743
	7	Br	-30.30724716	0.129363381
		Cl	-29.9880519	0.128000929

Supplementary Table 2. Titer benchmarking for halo-tryptamine production. Benchmarking estimated coculture titers from Supplementary Data 2 against commercially available analytical standards for 5-chloro-tryptamine and 5-bromo-tryptamine. Exact titers were determined via HPLC via comparison with an analytical standard and are graphically represented in Supplementary Figure 53. Estimated titers were determined via the method described in Supplementary Data 2.

Product	Exact titer via analytical standard (mg/L)	Estimated titer from Supplementary Data 2 (mg/L)
5-Cl-Tryptamine	38.7 +/- 1.4	58.4
5-Br-Tryptamine	51.7 +/- 6.4	50.2

Supplementary Table 3. Plasmids used in this study.

Plasmid name	Description	Source	Purpose
pHal	$pBR322^{ori}$, Amp^{R}, P_{TacI}	This study	Halogenase expression vector
p15A	$p15A^{ori}$, Cm^{R}, P_{500}	This study	Trp Operon (pTrpOp) and downstream enzyme expression vector

Supplementary Table 4. Primers used in this study (synthesized by IDT).

Primer name	Sequence	Purpose
pHal-iPCR-R	ggatcctctccttgtgtga	Amplify pHal backbone
pHal-iPCR-F	taactcgagagagaatataaaaagcc	Amplify pHal backbone
pTacI-ThHal-F*	ggataacaatttcacacaaggagaggatccATGCTTAATAATGTCGTTAT	Amplify ThHal Gibson fragment
pTacI-ThHal-R*	atctggctttttatattctctctcgagttaTTAACGCAGTTGGGTAAA	Amplify ThHal Gibson fragment
pTaci-PyrH-F	acaatttcacacaaggagaggatccATGGAACGCCGTAAACGT	Amplify PyrH Gibson fragment
pTaci-PyrH-R	atctggctttttatattctctctcgagttaTTACTGAATGCTTGCCAGATATTC	Amplify PyrH Gibson fragment
pHal-iPCR-2-R	ttgcgccttgagcgacac	Amplify pHal backbone to insert Th-Fre
pHal-iPCR-2-F	agcttgtcgaccctgcattagg	Amplify pHal backbone to insert Th-Fre
TrpR::Kan Lambda red primer-F	TGTTATTAGTTCGTTACTGGAAGTCCAGTCACCTTGTCAGGAGTATTATCattccggggatccgtcgacc	Generate TrpR homology fragment for LR deletion
TrpR::Kan Lambda red primer-R	AAAGCGGGTATAAATTCGCCCATCCGTTGCAGATGGGCGAGTAAGAAGTAgtgtaggctggagctgcttc	Generate TrpR homology fragment for LR deletion
TnaA::Kan Lambda red primer-F	TGTAATATTCACAGGGATCACTGTAATTAAAATAAATGAAGGATTATGTAattccggggatccgtcgacc	Generate TnaA homology fragment for LR deletion
TnaA::Kan Lambda red primer-R	TGTAGGGTAAGAGAGTGGCTAACATCCTTATAGCCACTCTGTAGTATTAAgtgtaggctggagctgcttcg	Generate TnaA homology fragment for LR deletion
RbsAR Integration Cassette PCR-F	gttcatctttcggttggtgg	Amplify int. cassette containing AroG, SerA with RbsAR homology
RbsAR Integration Cassette PCR-R	gtgtatatctccgaagaccgtaa	Amplify int. cassette containing AroG, SerA with RbsAR homology

*Note: Similar primers were utilized to amplify all other listed halogenases with identical Gibson assembly homology

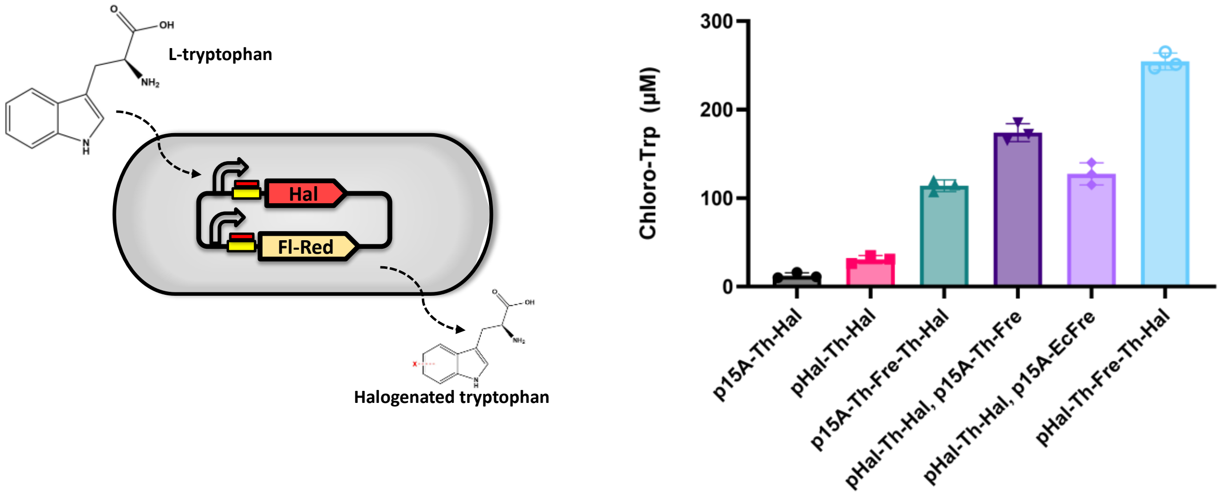

Supplementary Fig. 1. Production of 6-chloro-tryptophan (6-Cl-Trp) via varied expression regimes. Strain sKR-Trp0 harboring plasmids with varying expression regimes for halogenase Th-Hal and flavin reductases Fre and Th-Fre were grown for 24-hour incubation with 1mM of L-tryptophan fed in biological triplicate. Error bars represent S.E. of n=3 biological triplicates. Source data are provided as a Source Data file.

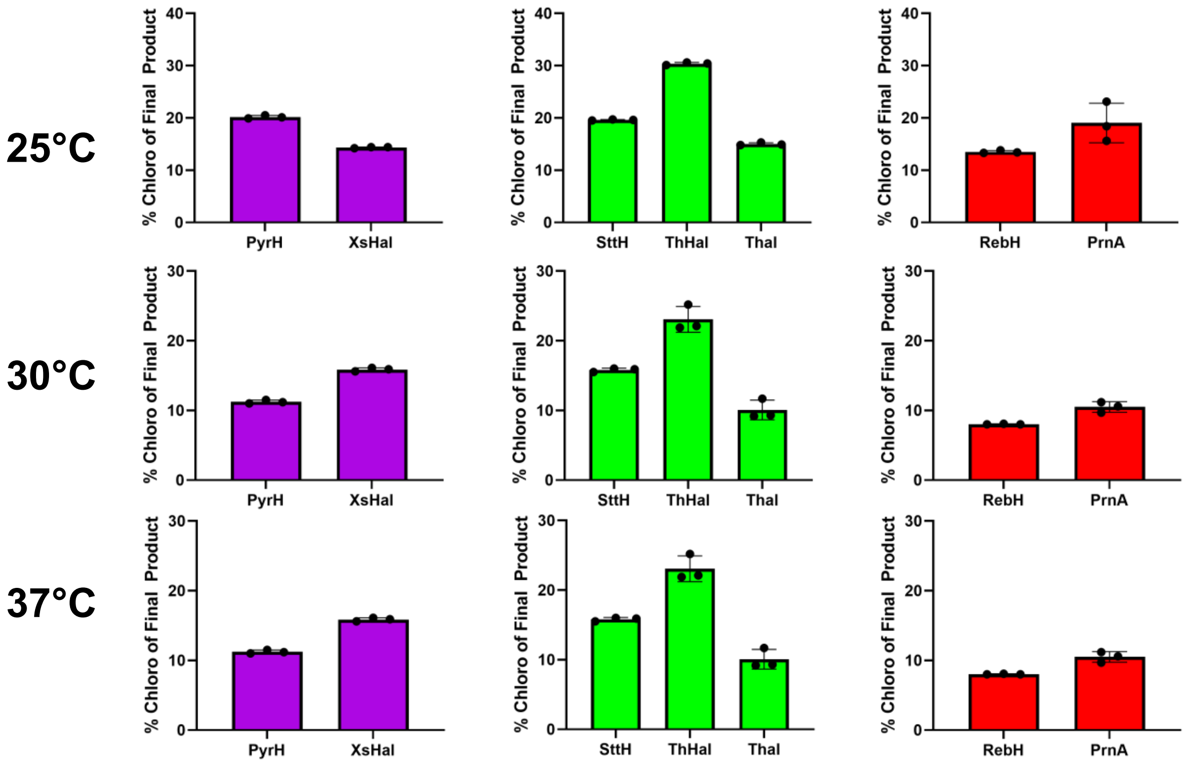

Supplementary Fig. 2. Residual chloro-tryptophan from halogenase panel. Percentage of chloro-tryptophan in final production for halogenase panel with media containing primarily NaBr salt. Error bars represent S.E. of n=3 biological replicates. Source data are provided as a Source Data file.

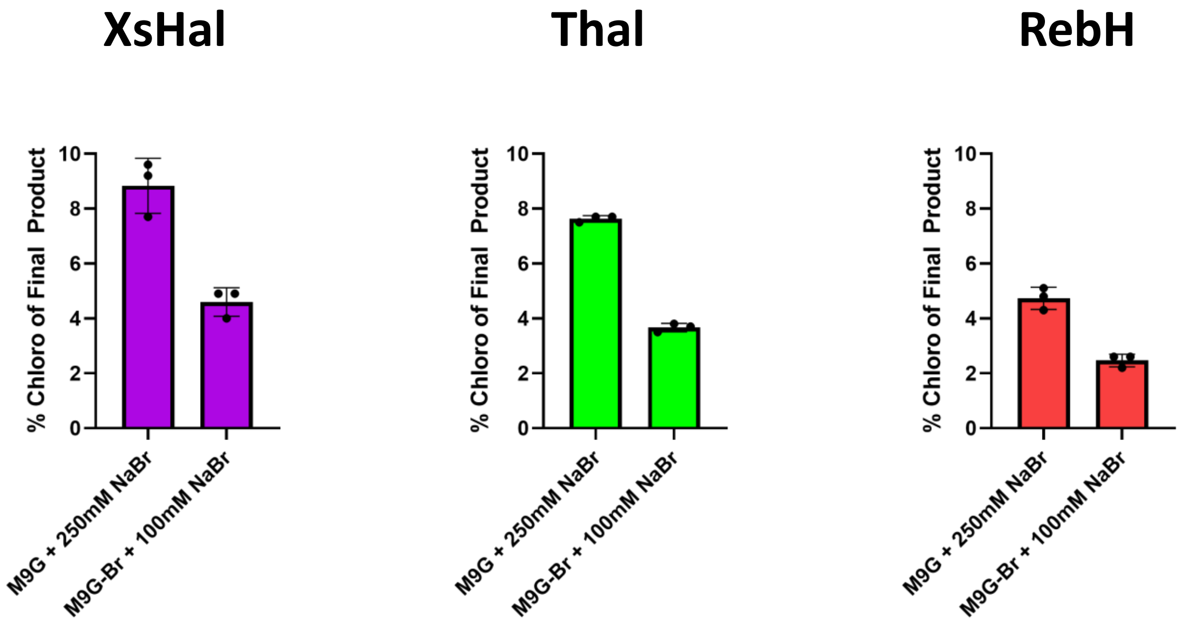

Supplementary Fig. 3. Comparison of media formulations for production of bromo-tryptophan. M9G is conventional M9 media with 0.4% glucose. Excess NaBr (250 mM) was added to this media. In comparison, media denoted M9G-Br has the typical formulation of M9 however all the chloride salts replaced with bromide salts (ammonium bromide, sodium bromide, etc.). An additional 100mM NaBr was added to this media. Error bars represent S.E. of n=3 biological replicates. Source data are provided as a Source Data file.

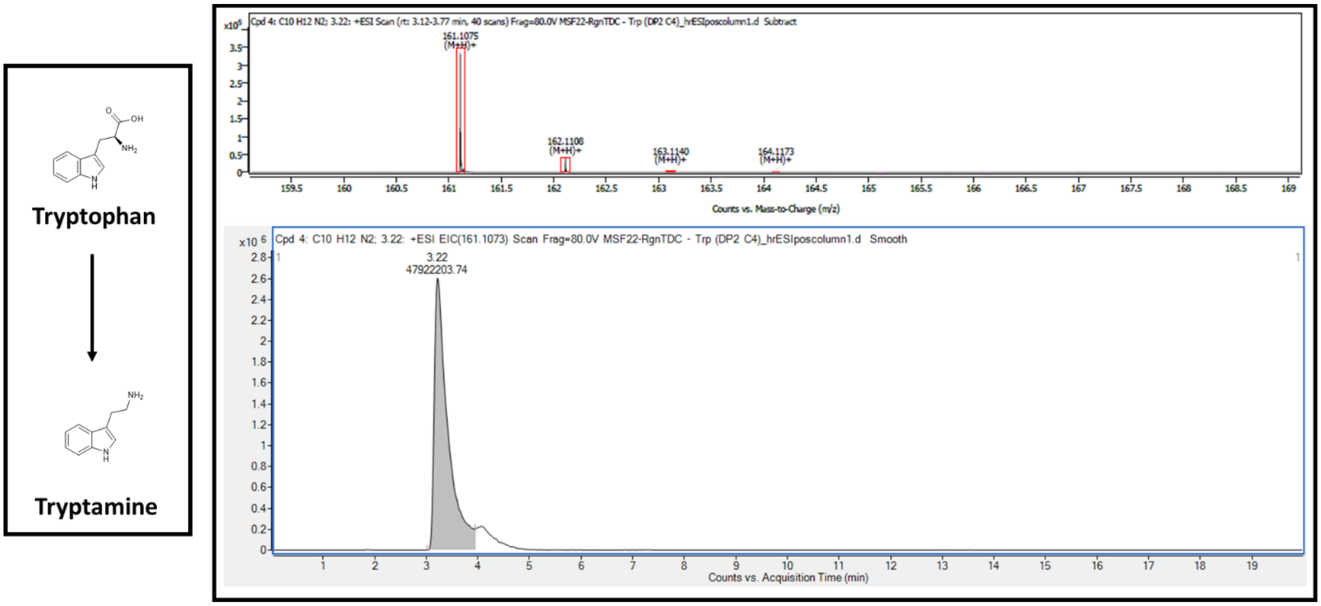

Supplementary Fig. 4. Analytical confirmation of formation of tryptamine from tryptophan.

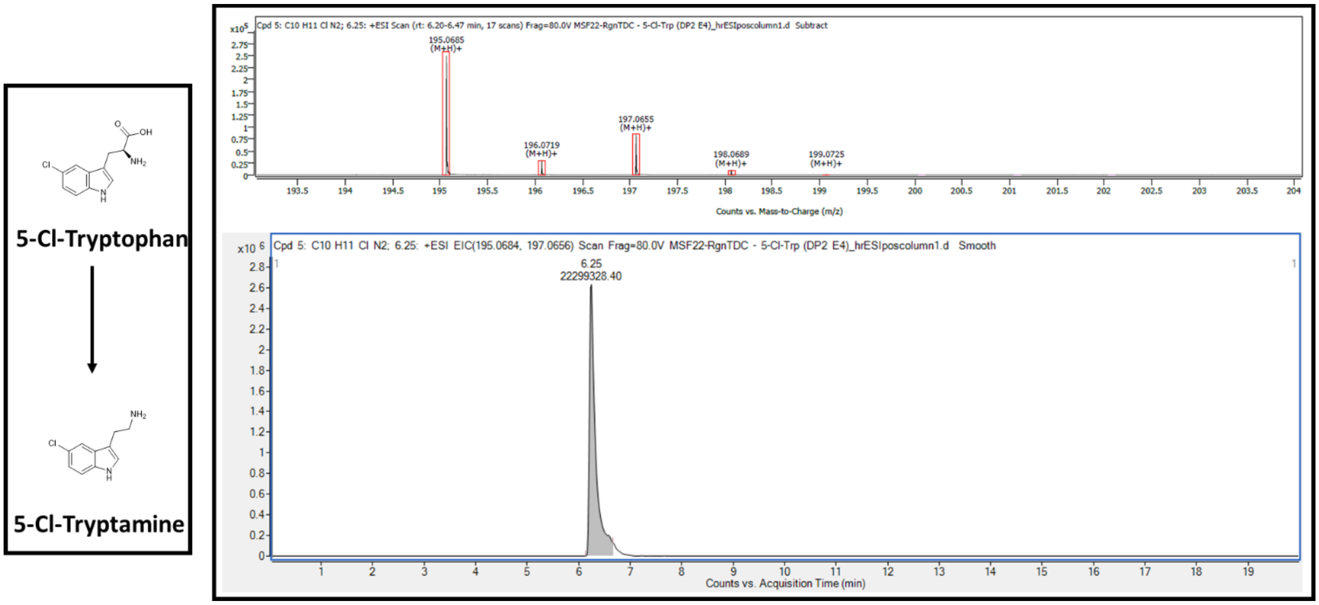

Supplementary Fig. 5. Analytical confirmation of formation of 5-Cl-tryptamine from 5-Cl-tryptophan.

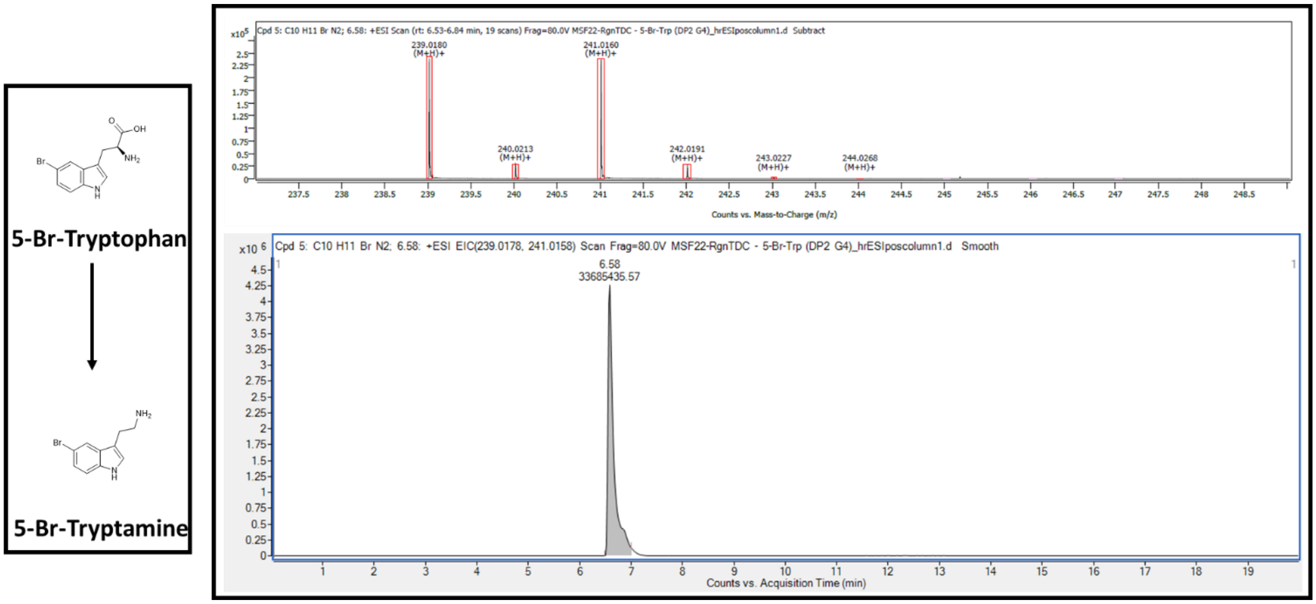

Supplementary Fig. 6. Analytical confirmation of formation of 5-Br-tryptamine from 5-Br-tryptophan.

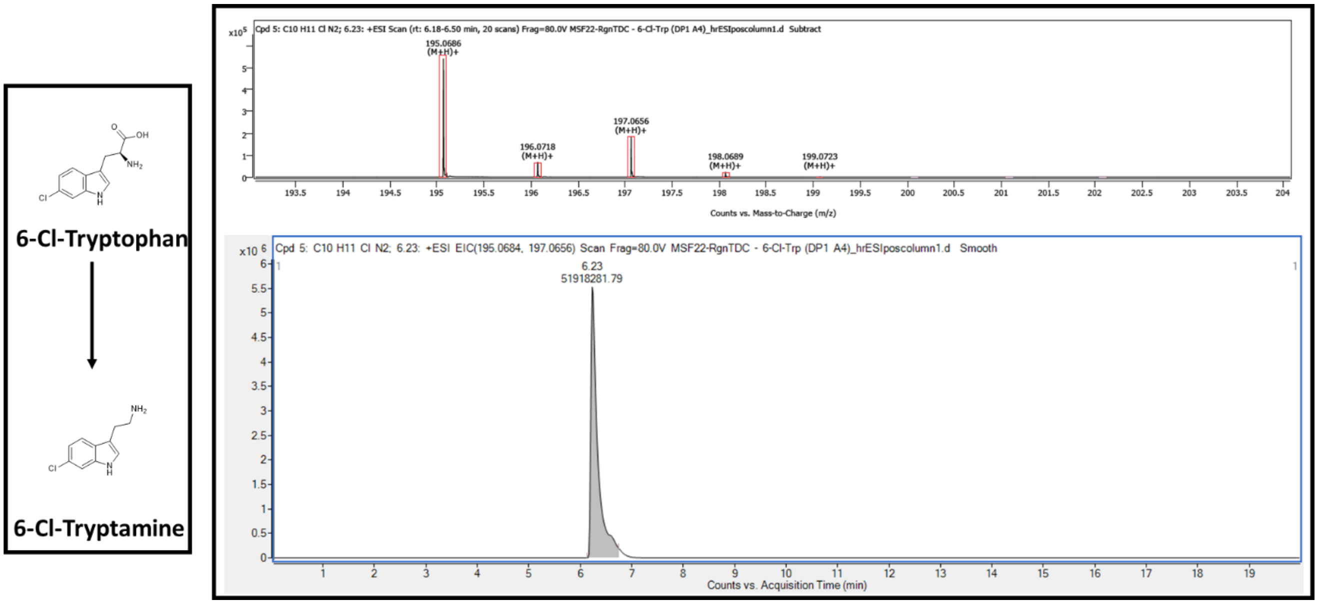

Supplementary Fig. 7. Analytical confirmation of formation of 6-Cl-tryptamine from 6-Cl-tryptophan.

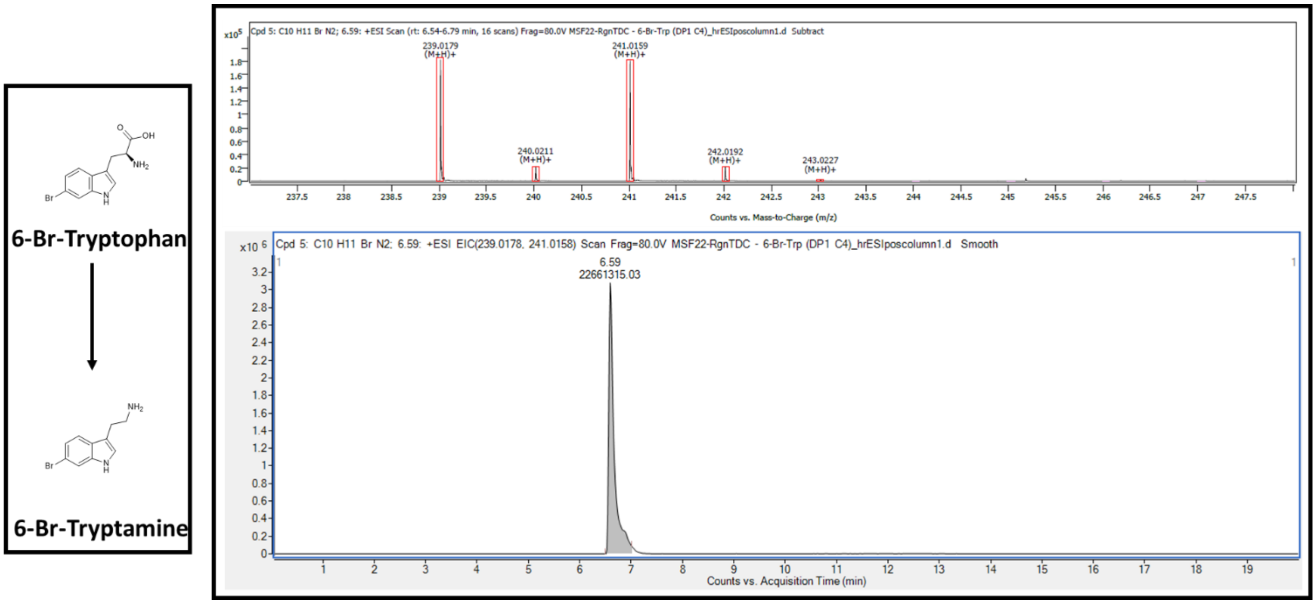

Supplementary Fig. 8. Analytical confirmation of formation of 6-Br-tryptamine from 6-Br-tryptophan.

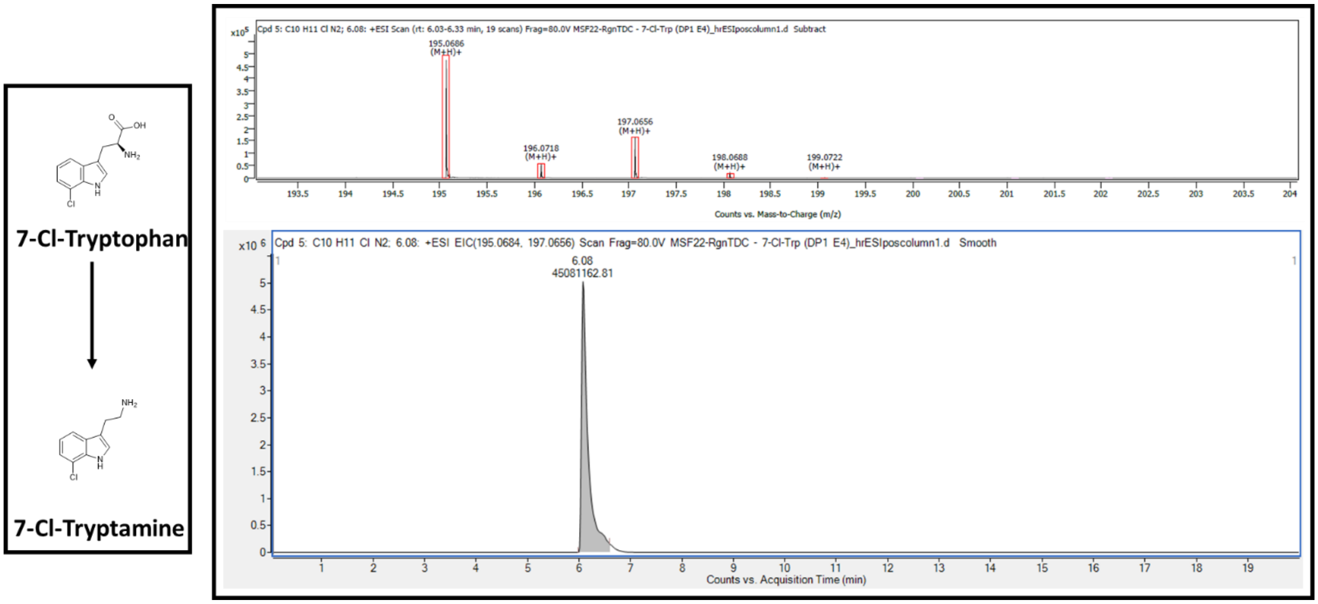

Supplementary Fig. 9. Analytical confirmation of formation of 7-Cl-tryptamine from 7-Cl-tryptophan.

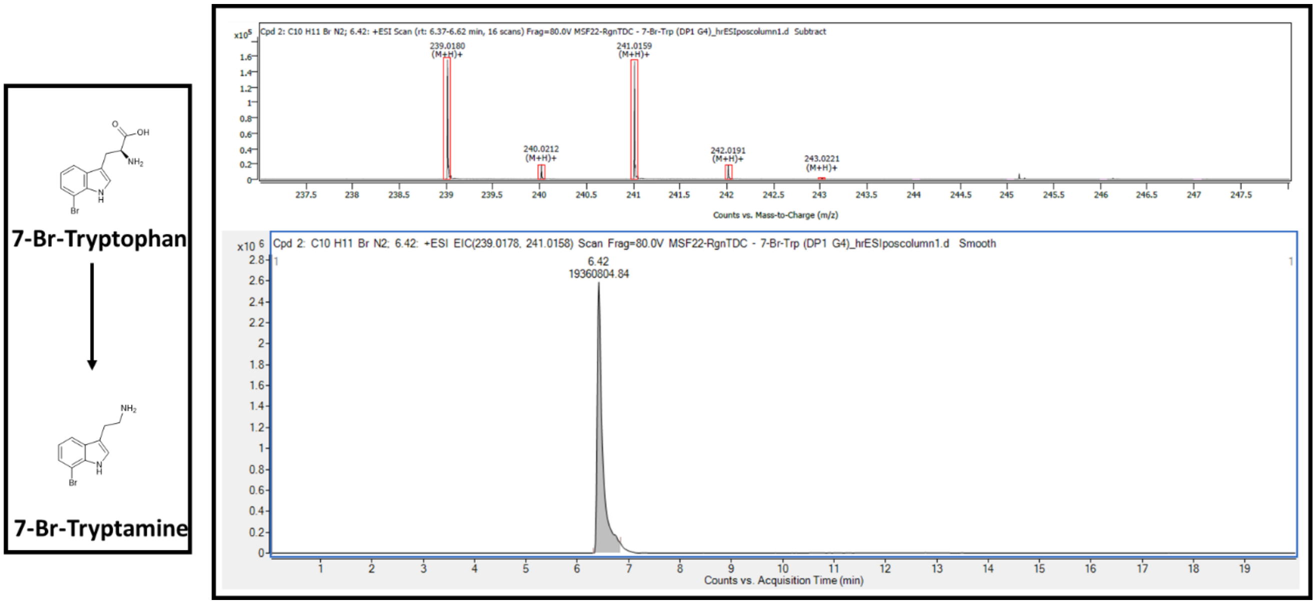

Supplementary Fig. 10. Analytical confirmation of formation of 7-Br-tryptamine from 7-Br-tryptophan.

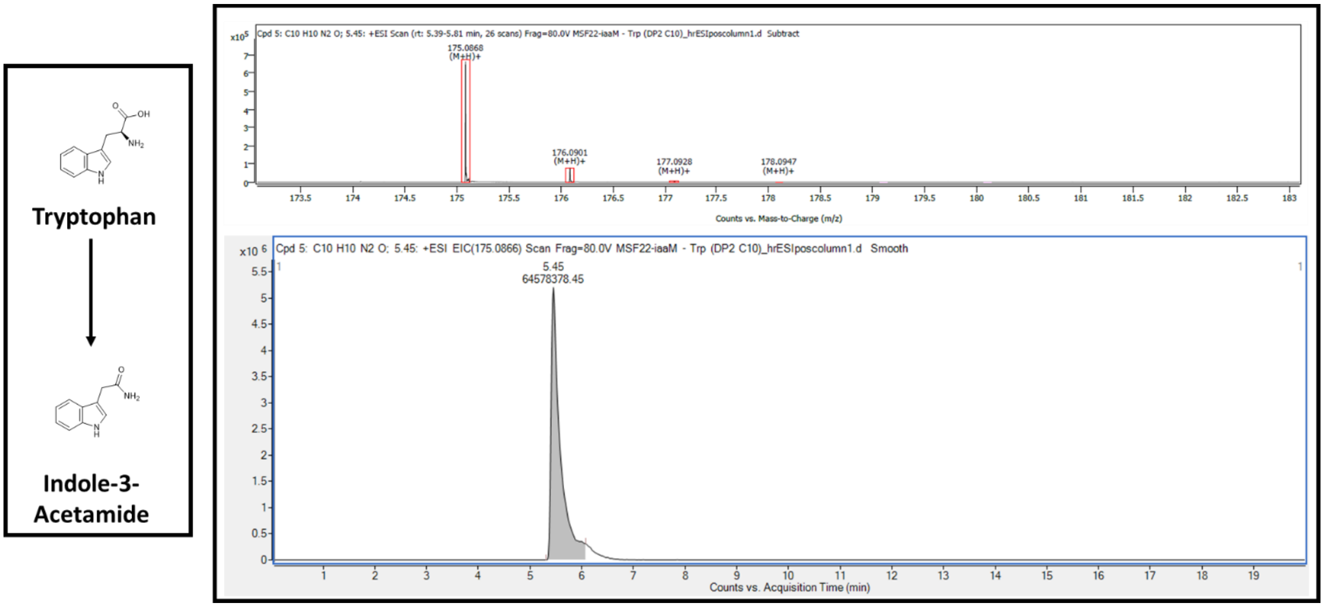

Supplementary Fig. 11. Analytical confirmation of formation of indole-3-acetamide from tryptophan.

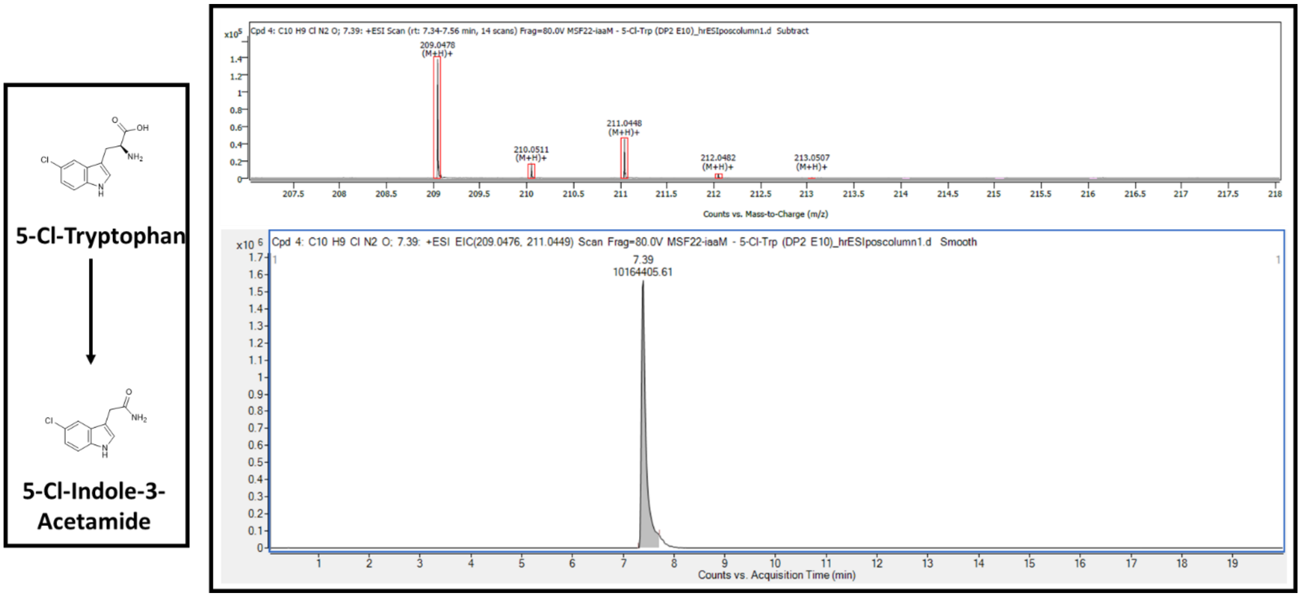

Supplementary Fig. 12. Analytical confirmation of formation of 5-Cl-indole-3-acetamide from 5-Cl-tryptophan.

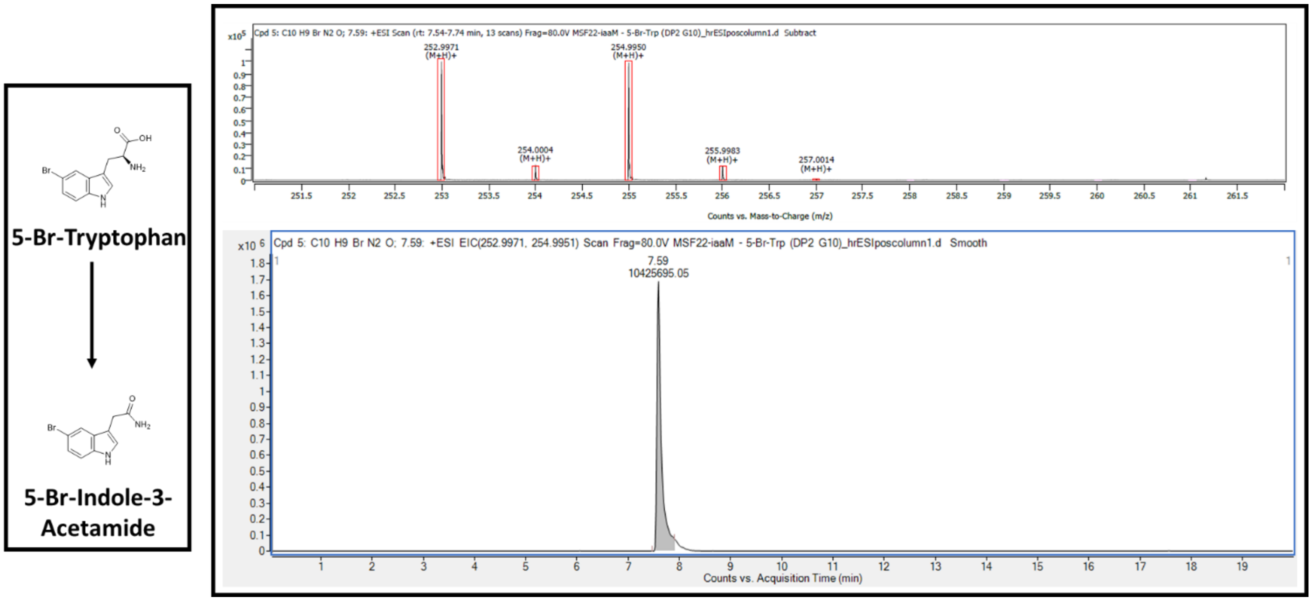

Supplementary Fig. 13. Analytical confirmation of formation of 5-Br-indole-3-acetamide from 5-Br-tryptophan.

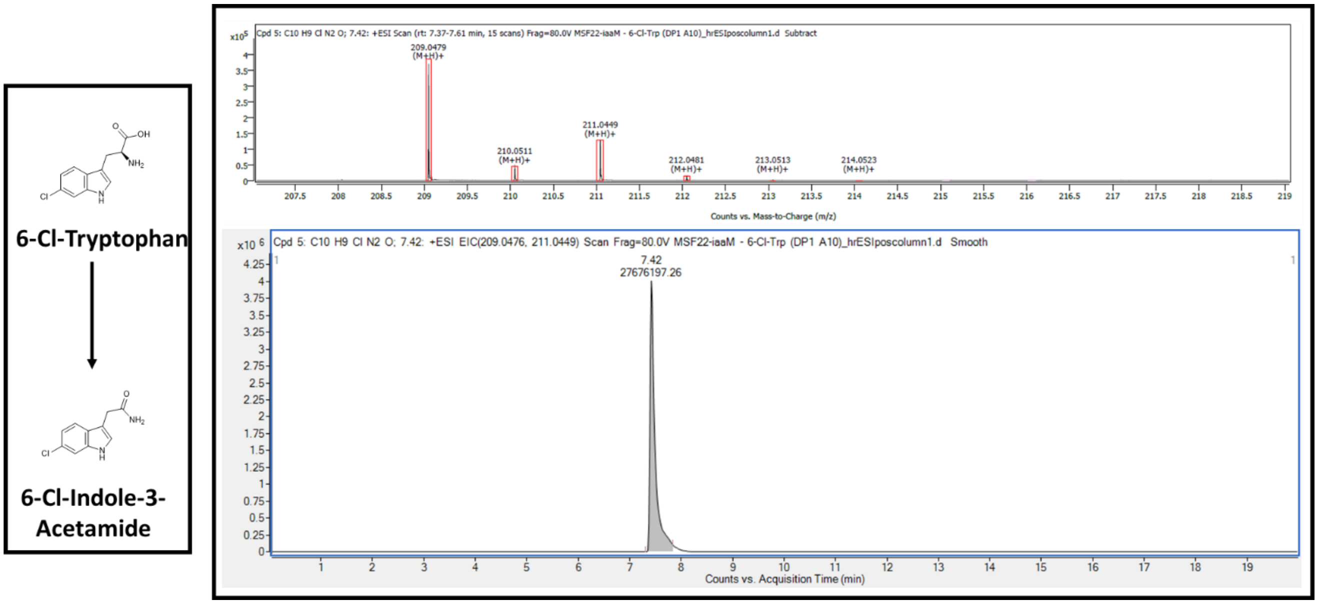

Supplementary Fig. 14. Analytical confirmation of formation of 6-Cl-indole-3-acetamide from 6-Cl-tryptophan.

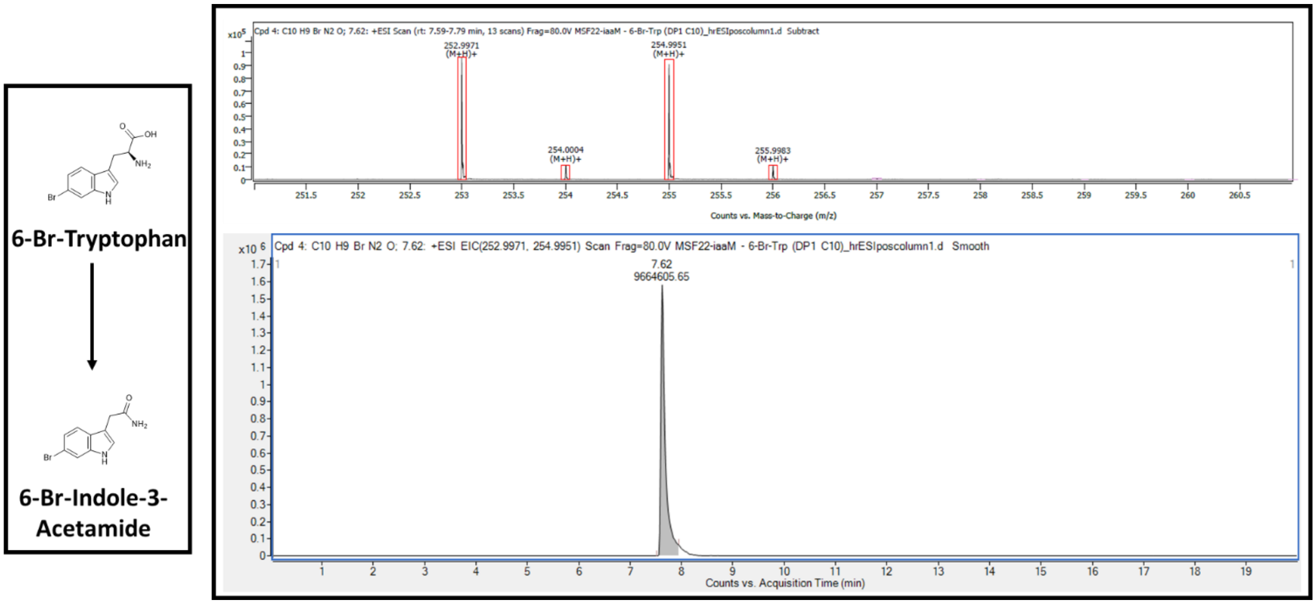

Supplementary Fig. 15. Analytical confirmation of formation of 6-Br-indole-3-acetamide from 6-Br-tryptophan.

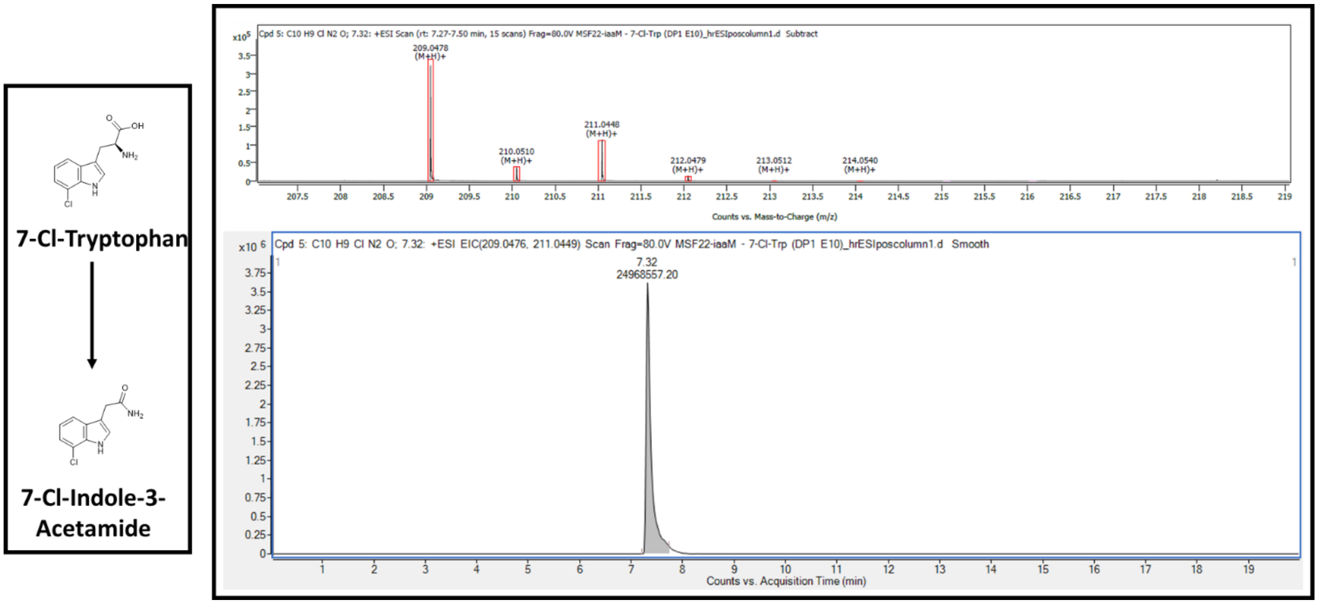

Supplementary Fig. 16. Analytical confirmation of formation of 7-Cl-indole-3-acetamide from 7-Cl-tryptophan.

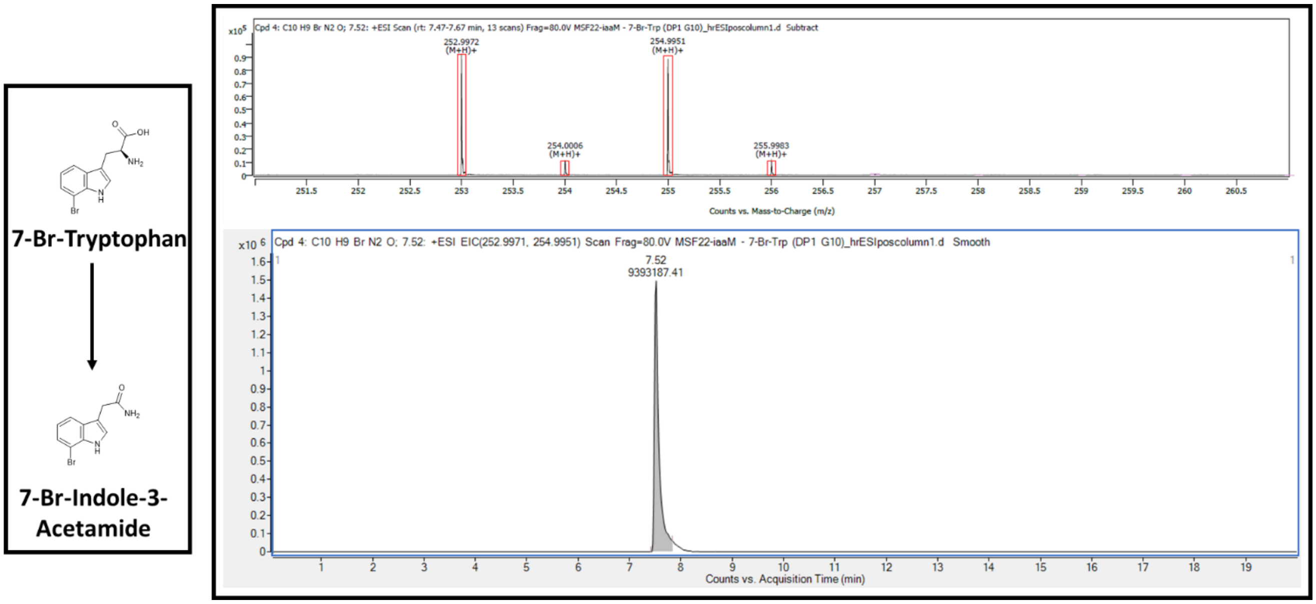

Supplementary Fig. 17. Analytical confirmation of formation of 7-Br-indole-3-acetamide from 7-Br-tryptophan.

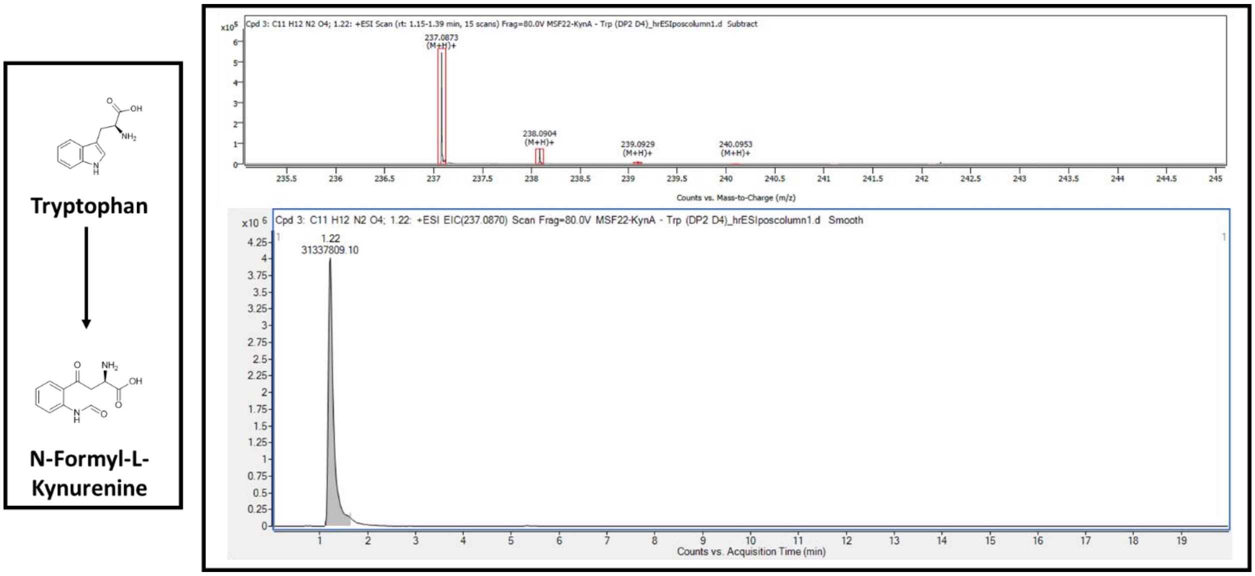

Supplementary Fig. 18. Analytical confirmation of formation of N-formyl-L-kynurenine from tryptophan.

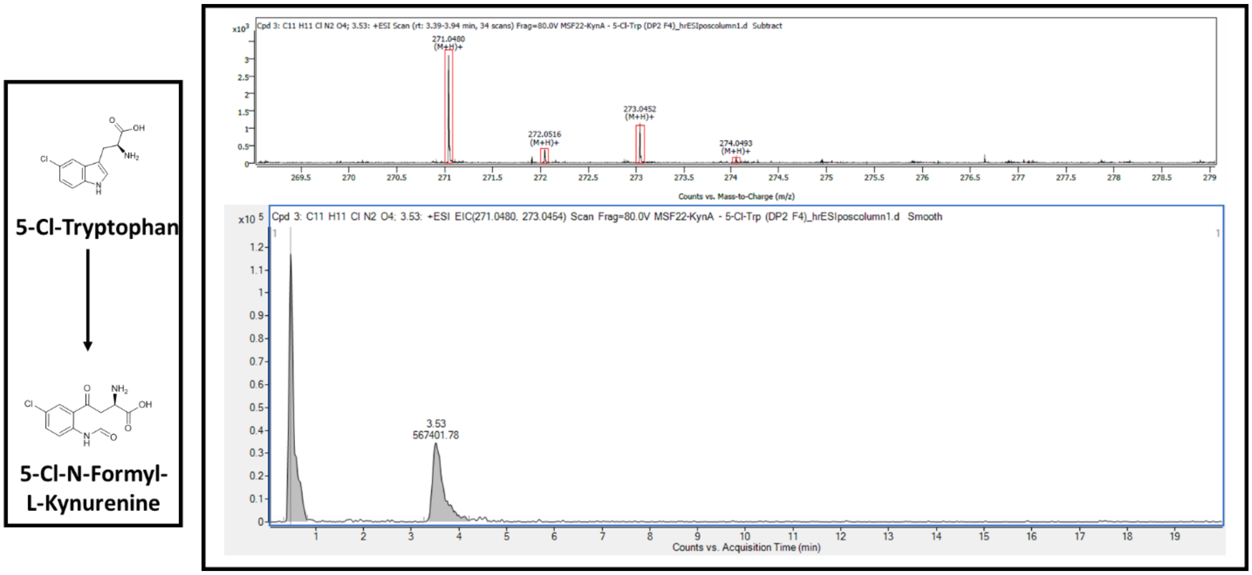

Supplementary Fig. 19. Analytical confirmation of formation of 5-Cl-N-formyl-L-kynurenine from 5-Cl-tryptophan.

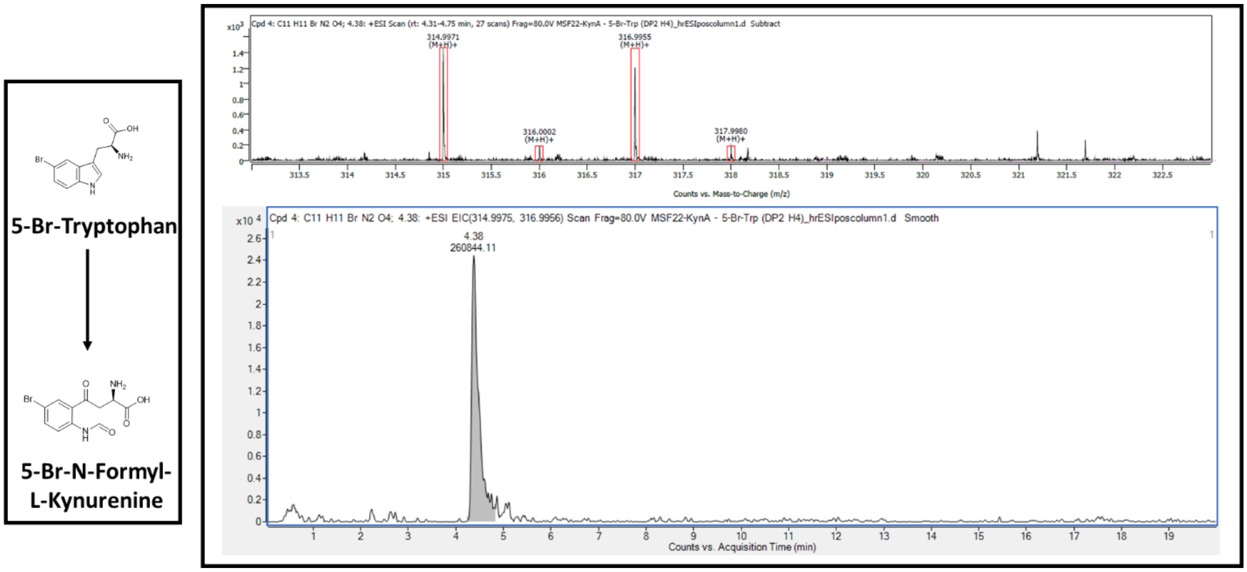

Supplementary Fig. 20. Analytical confirmation of formation of 5-Br-N-formyl-L-kynurenine from 5-Br-tryptophan.

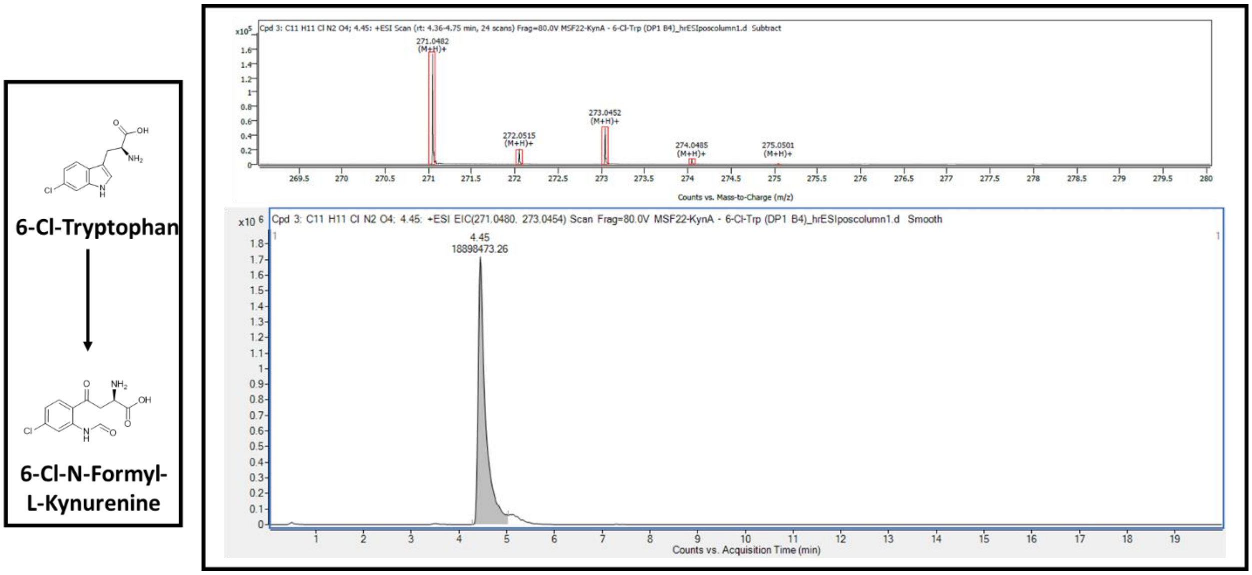

Supplementary Fig. 21. Analytical confirmation of formation of 6-Cl-N-formyl-L-kynurenine from 6-Cl-tryptophan.

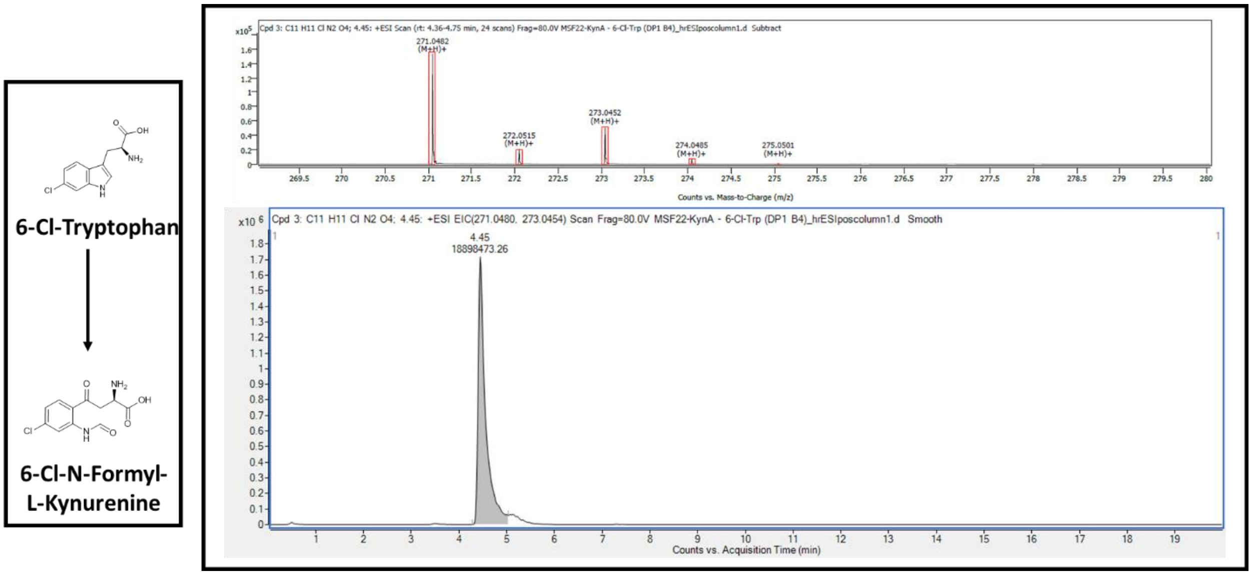

Supplementary Fig. 22. Analytical confirmation of formation of 6-Br-N-formyl-L-kynurenine from 6-Br-tryptophan.

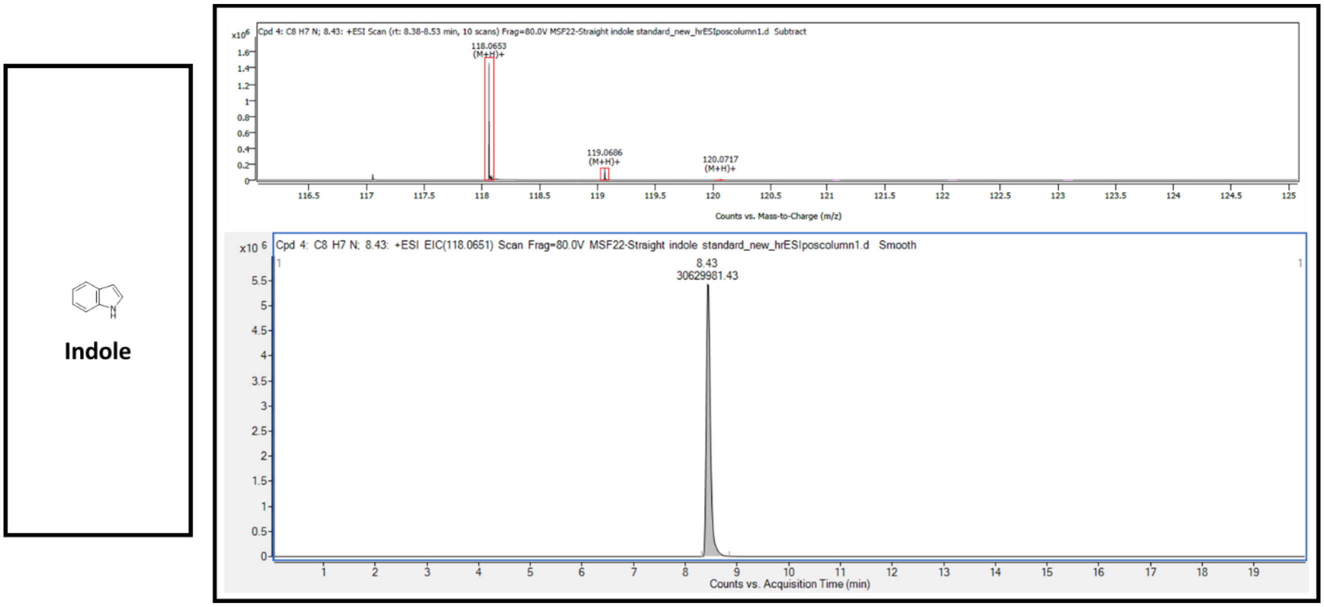

Supplementary Fig. 23. Analytical confirmation of indole standard.

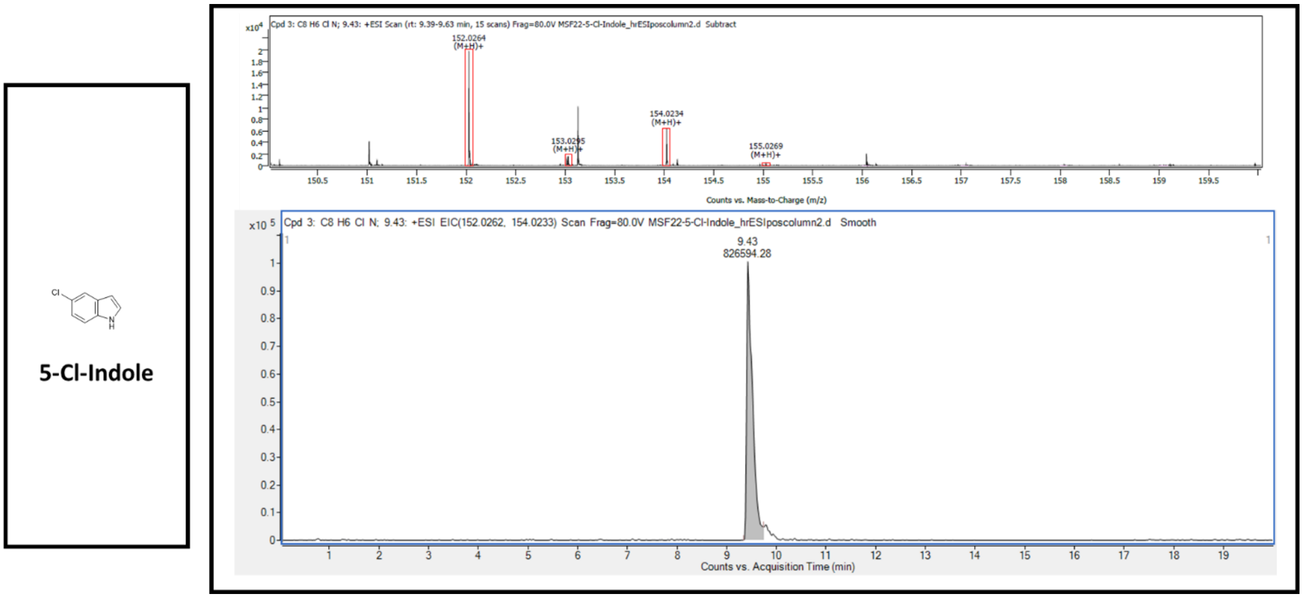

Supplementary Fig. 24. Analytical confirmation 5-Cl-indole standard.

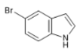

5-Br-Indole

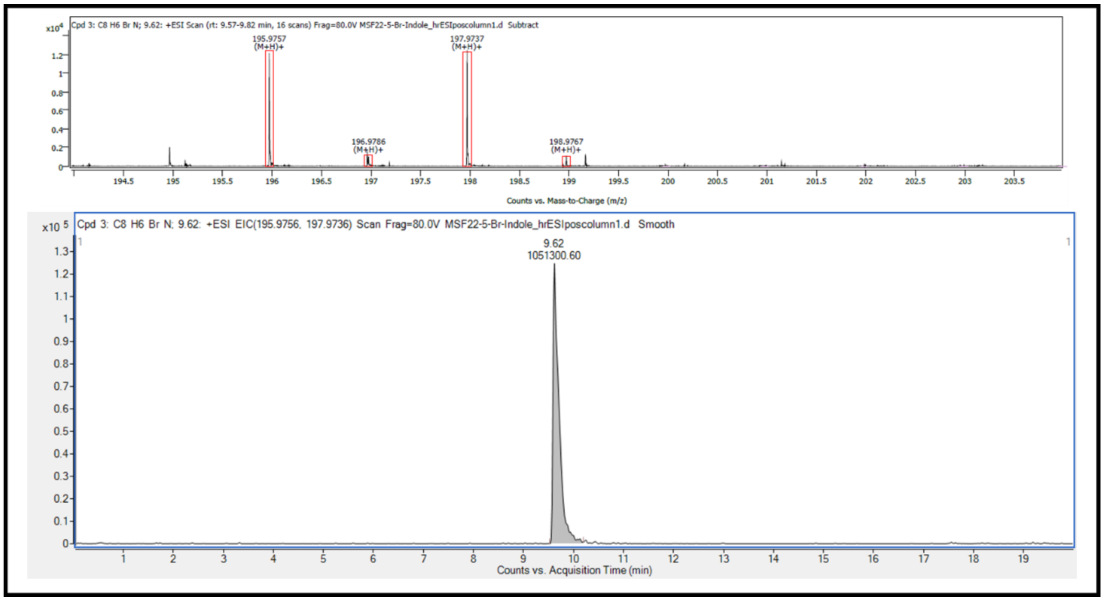

Supplementary Fig. 25. Analytical confirmation 5-Br-indole standard.

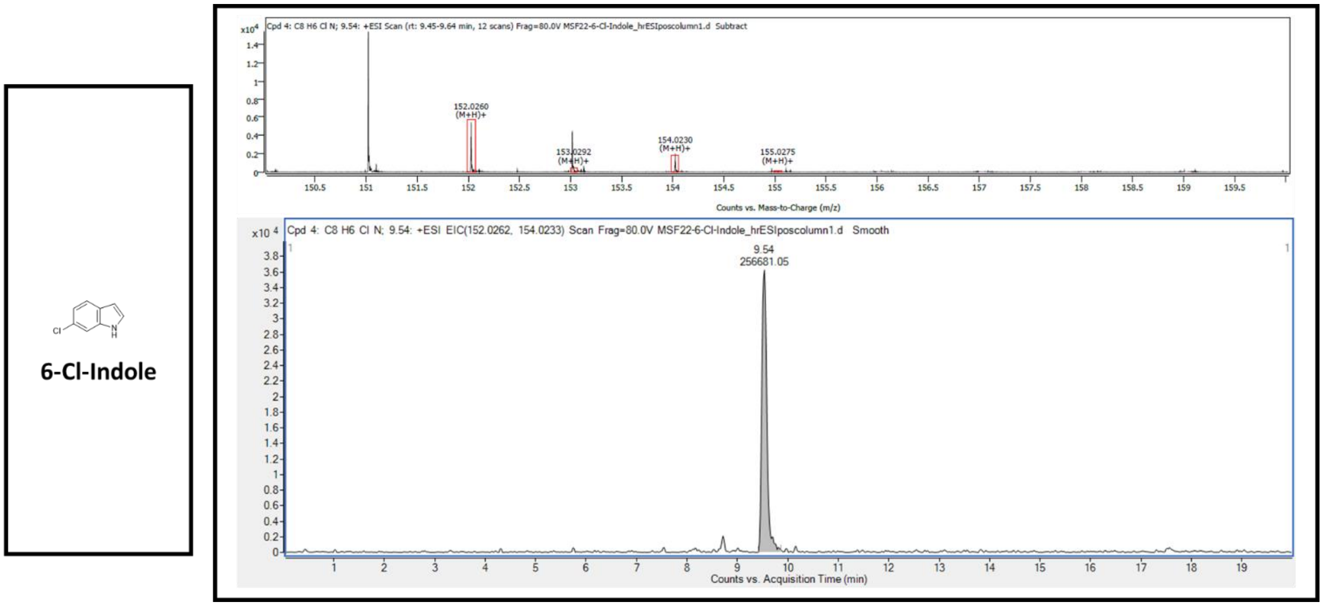

Supplementary Fig. 26. Analytical confirmation of 6-Cl-indole standard.

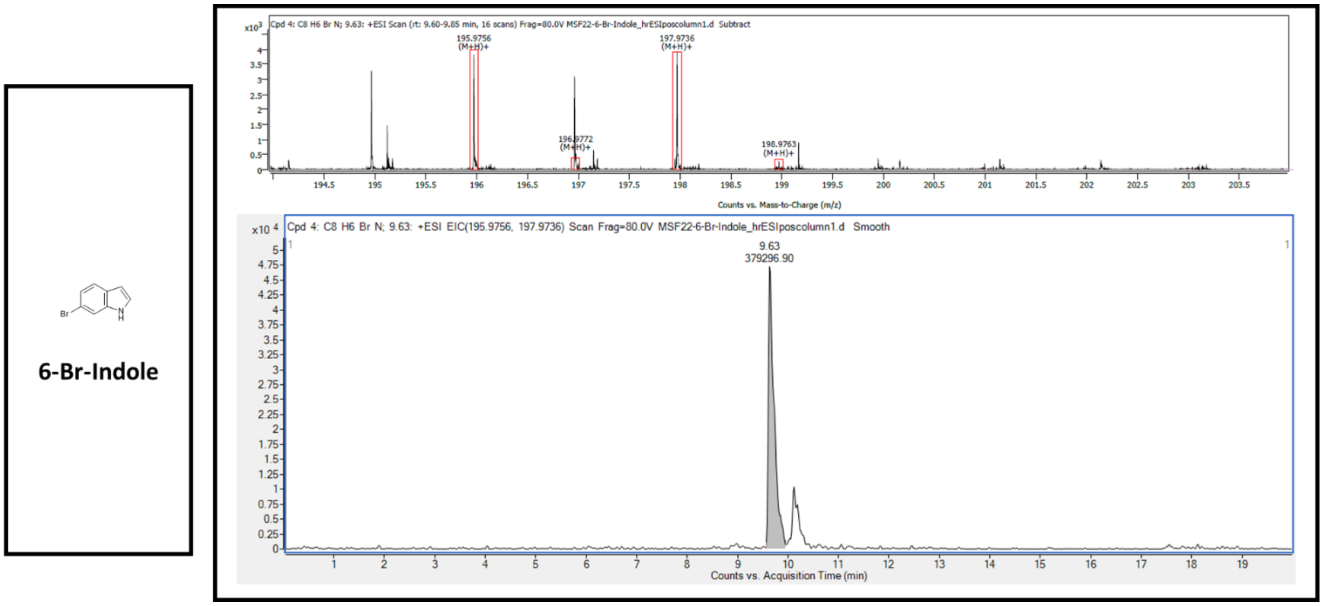

Supplementary Fig. 27. Analytical confirmation 6-Br-indole standard.

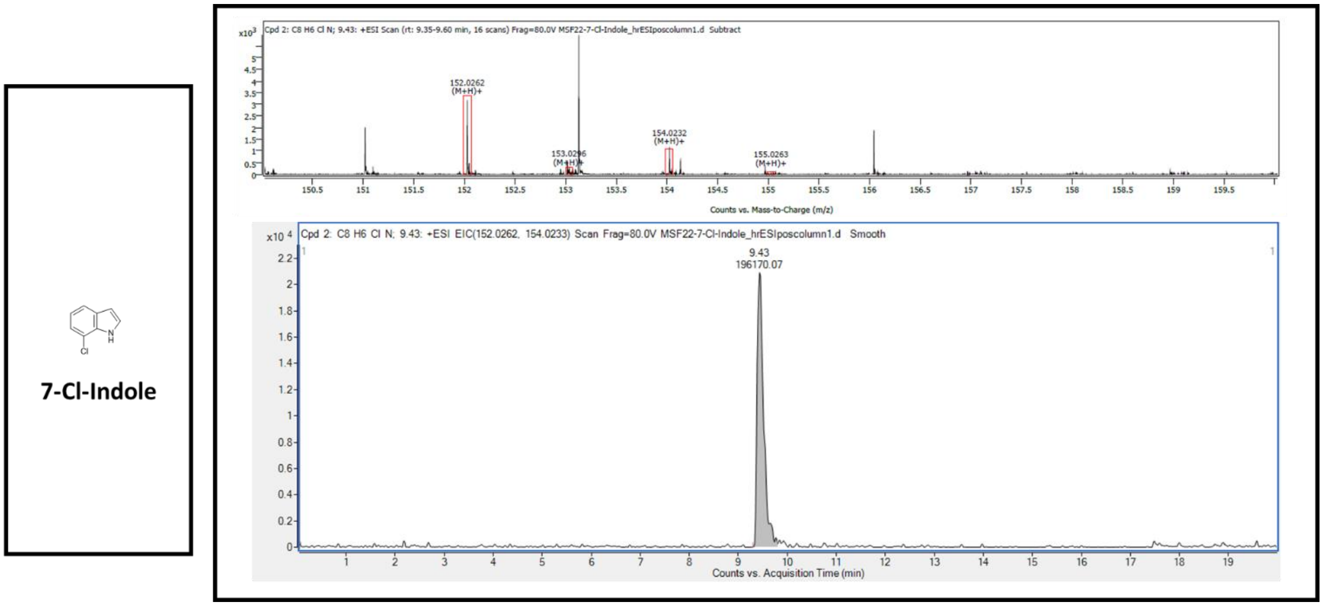

Supplementary Fig. 28. Analytical confirmation 7-Cl-indole standard.

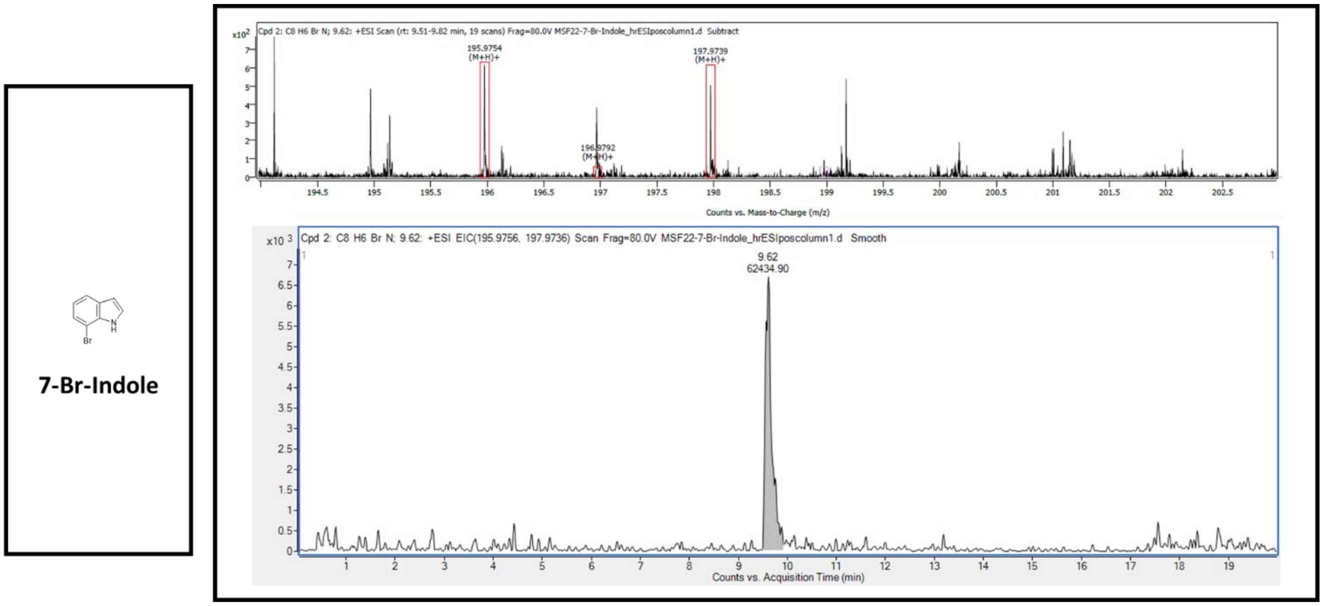

Supplementary Fig. 29. Analytical confirmation 7-Br-indole standard.

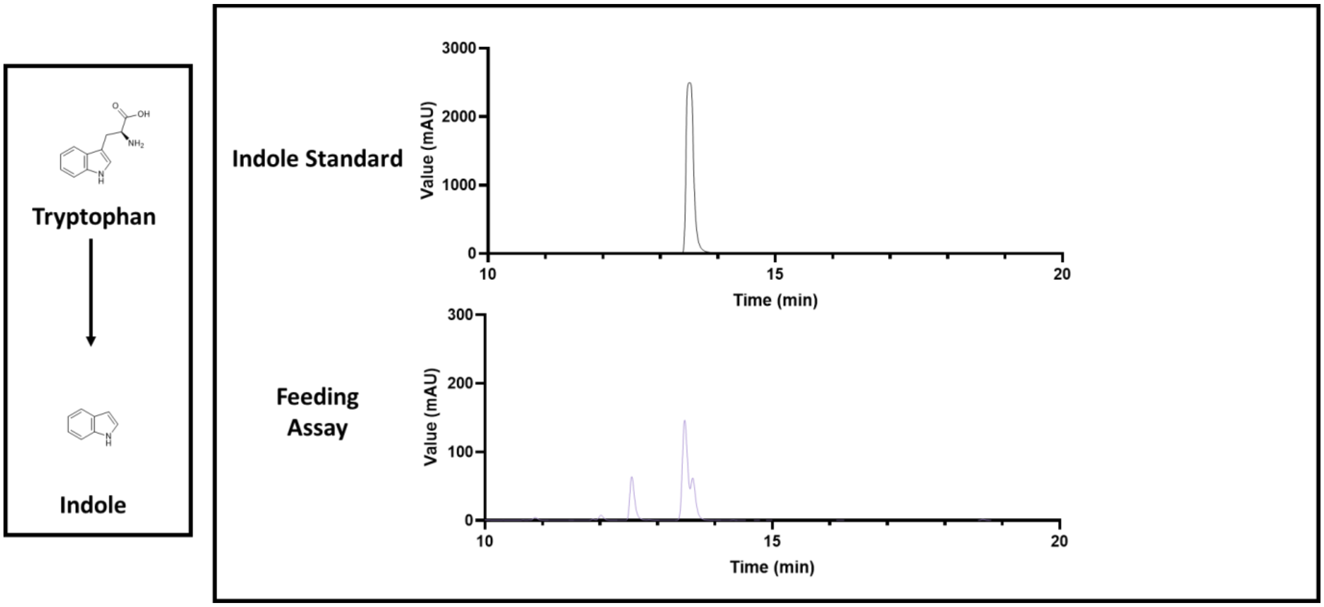

Supplementary Fig. 30. Analytical confirmation of formation of indole from tryptophan via HPLC.

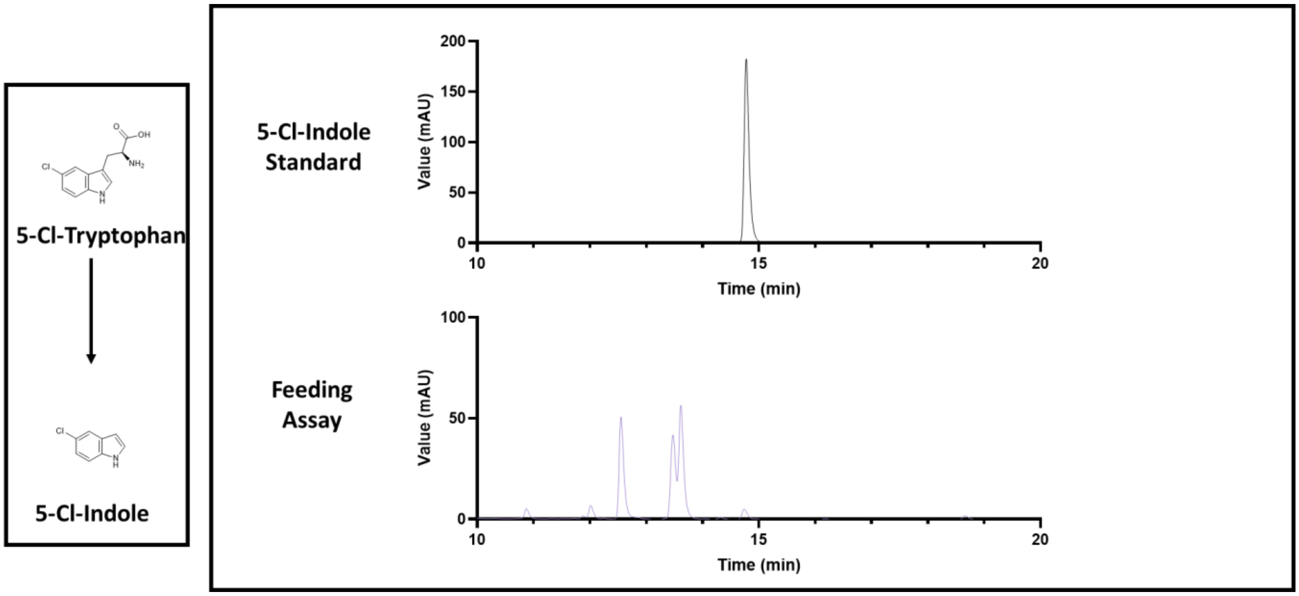

Supplementary Fig. 31. Analytical confirmation of formation of 5-Cl-indole from 5-Cl-tryptophan via HPLC.

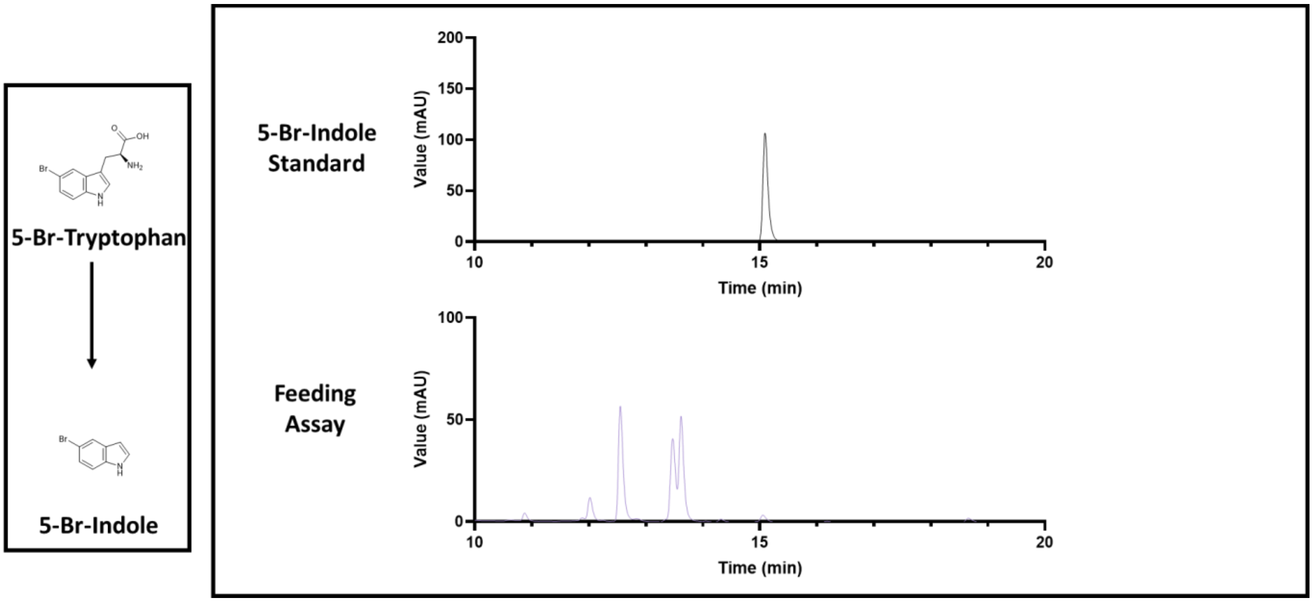

Supplementary Fig. 32. Analytical confirmation of formation of 5-br-indole from 5-Br-tryptophan via HPLC.

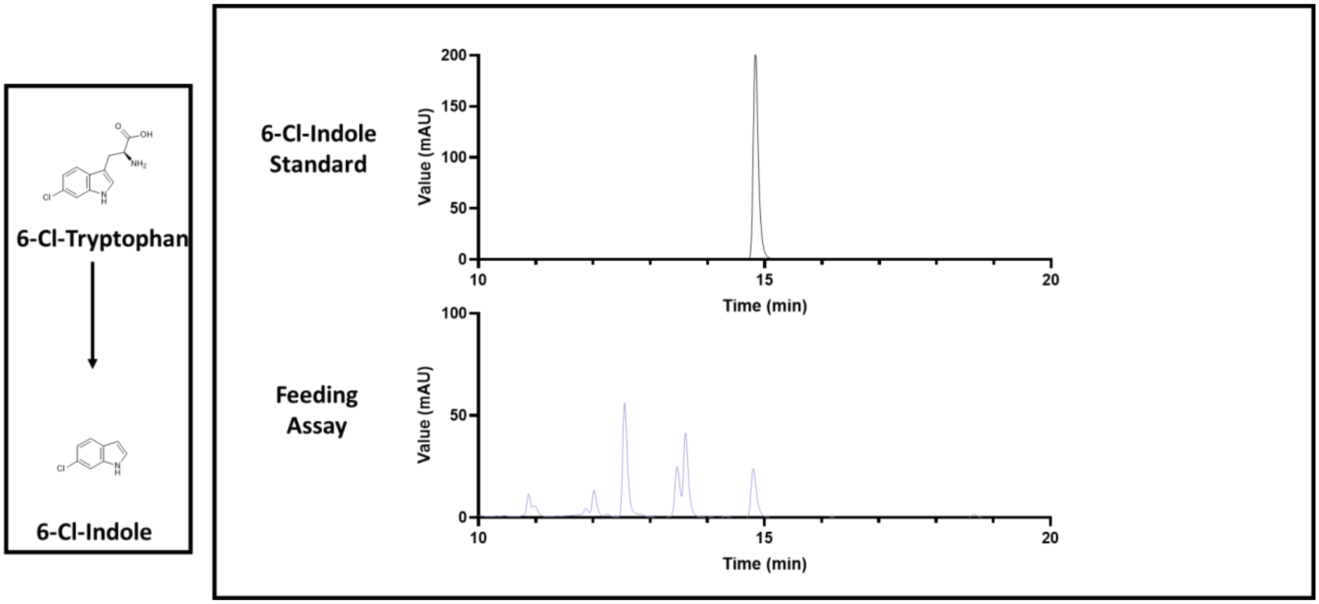

Supplementary Fig. 33. Analytical confirmation of formation of 6-Cl-indole from 6-Cl-tryptophan via HPLC.

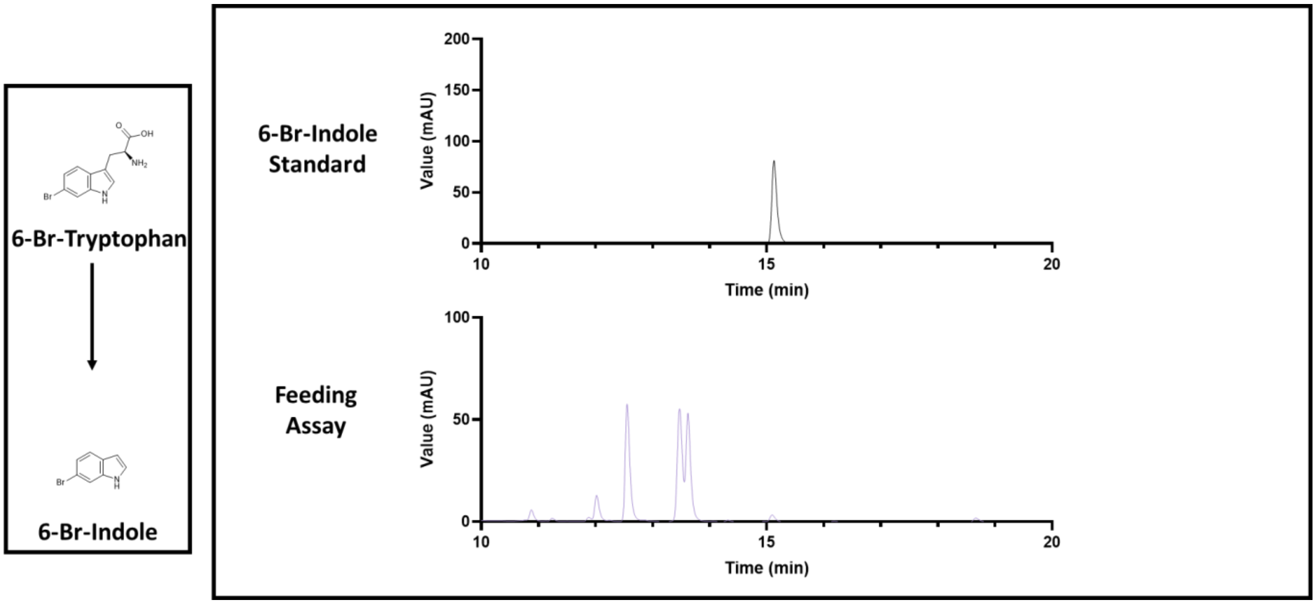

Supplementary Fig. 34. Analytical confirmation of formation of 6-Br-indole from 6-Br-tryptophan via HPLC.

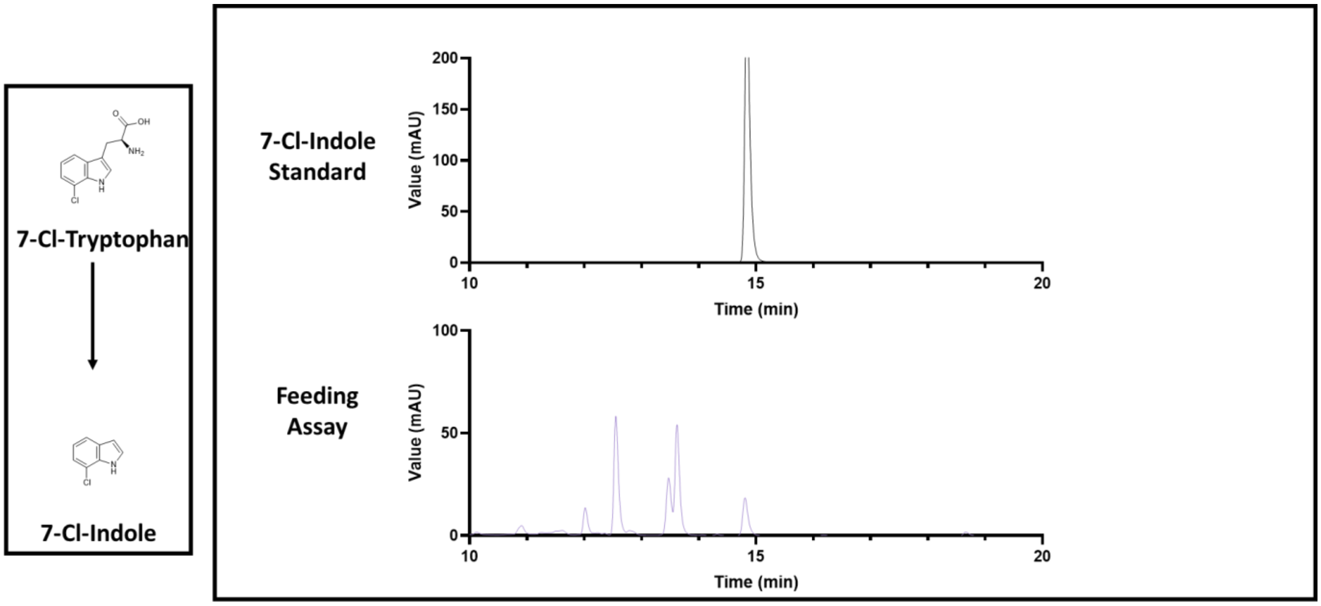

Supplementary Fig. 35. Analytical confirmation of formation of 7-Cl-indole from 7-Cl-tryptophan via HPLC.

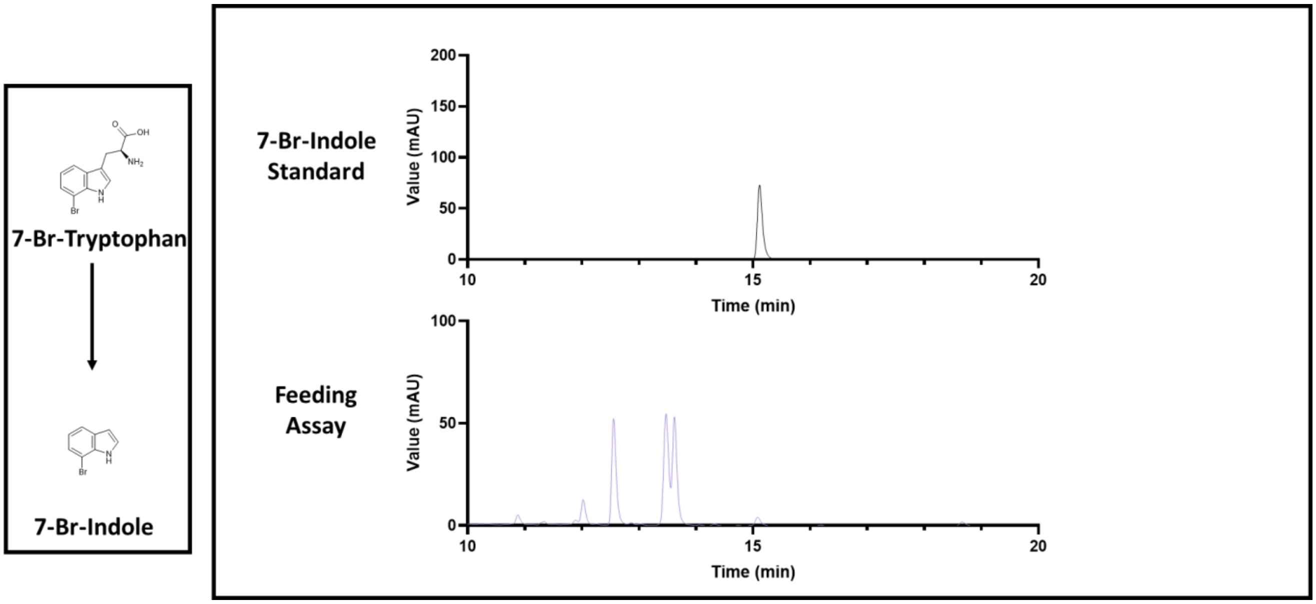

Supplementary Fig. 36. Analytical confirmation of formation of 7-Br-indole from 7-Br-tryptophan via HPLC.

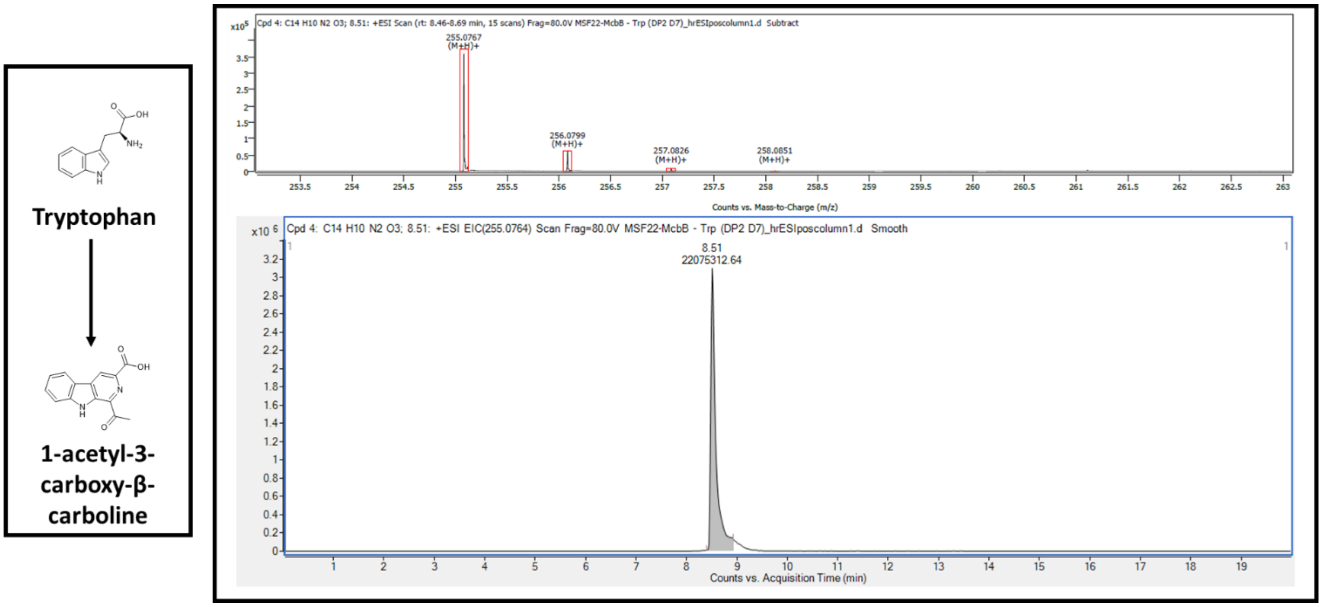

Supplementary Fig. 37. Analytical confirmation of formation of 1-acetyl-3-carboxyl-β-carboline from tryptophan.

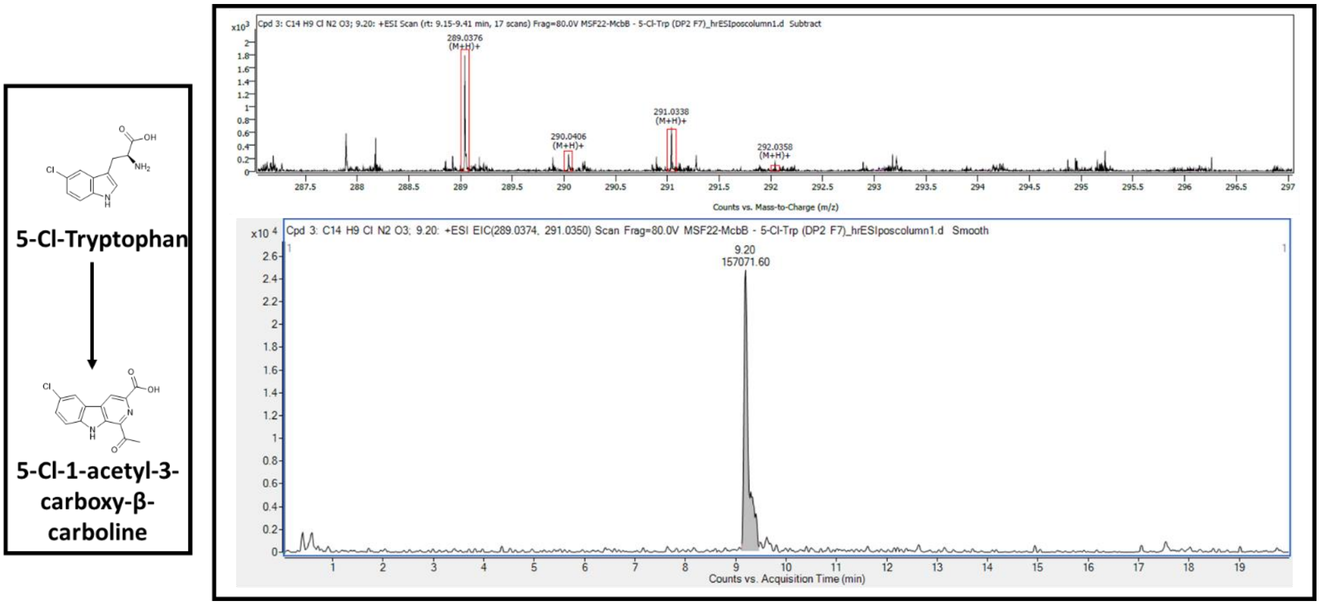

Supplementary Fig. 38. Analytical confirmation of formation of 5-Cl-1-acetyl-3-carboxyl-β-carboline from 5-Cl-tryptophan.

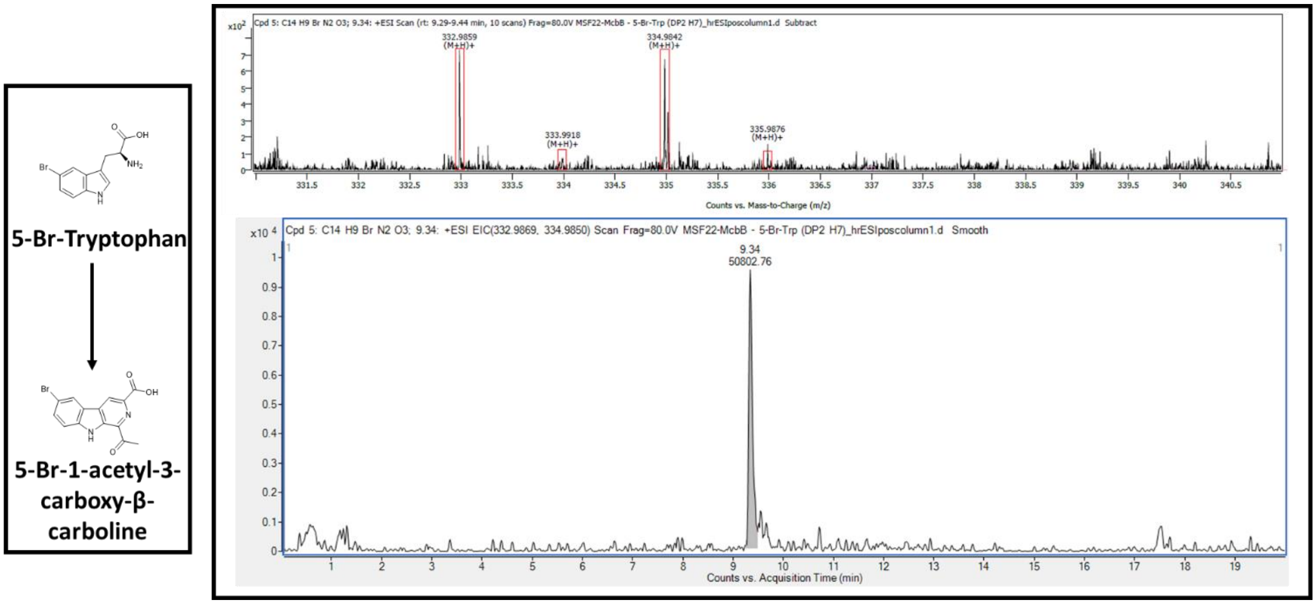

Supplementary Fig. 39. Analytical confirmation of formation of 5-Br-1-acetyl-3-carboxyl-β-carboline from 5-Br-tryptophan.

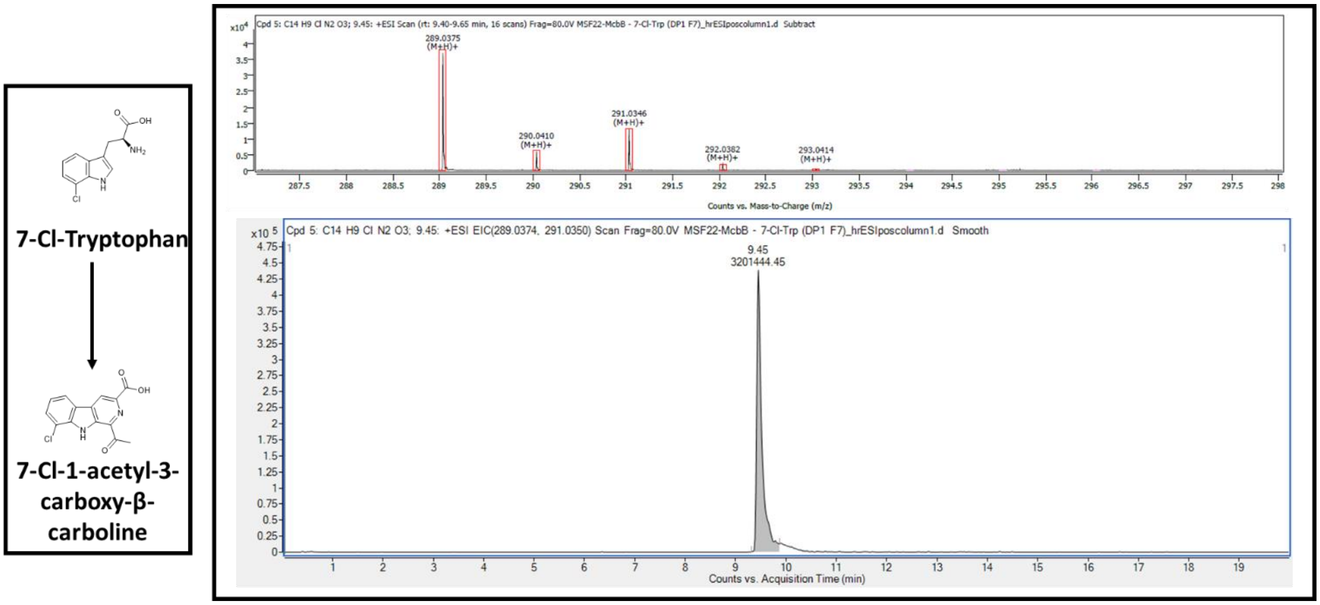

Supplementary Fig. 40. Analytical confirmation of formation of 7-Cl-1-acetyl-3-carboxyl-β-carboline from 7-Cl-tryptophan.

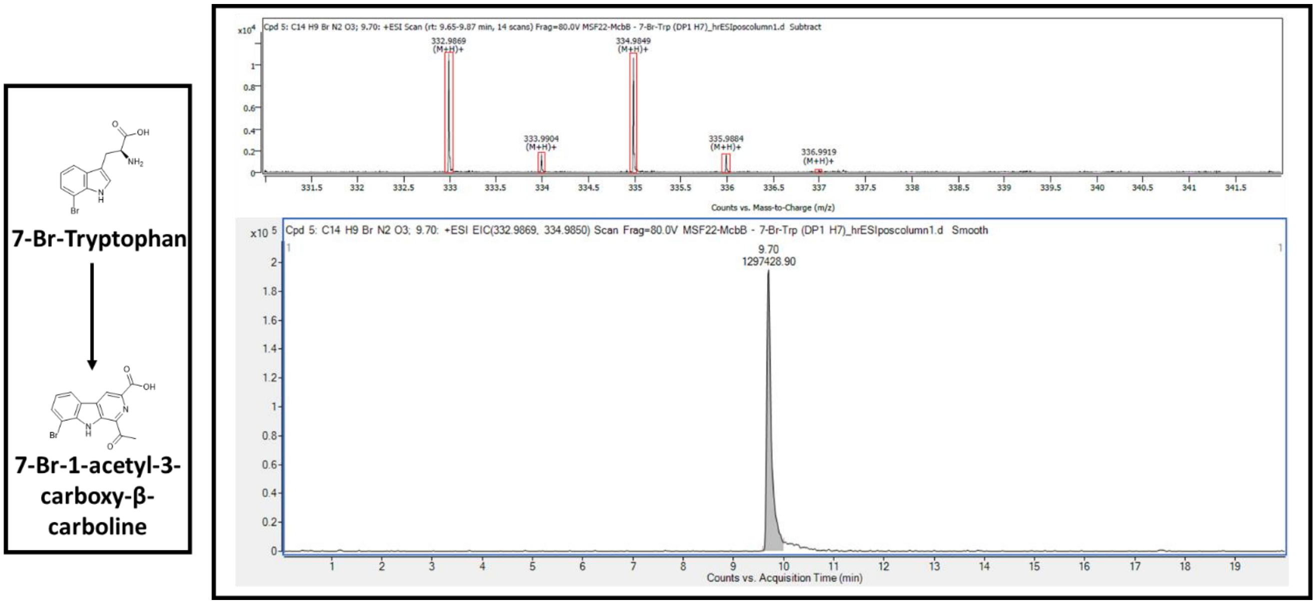

Supplementary Fig. 41. Analytical confirmation of formation of 7-Br-1-acetyl-3-carboxyl-β-carboline from 7-Br-tryptophan.

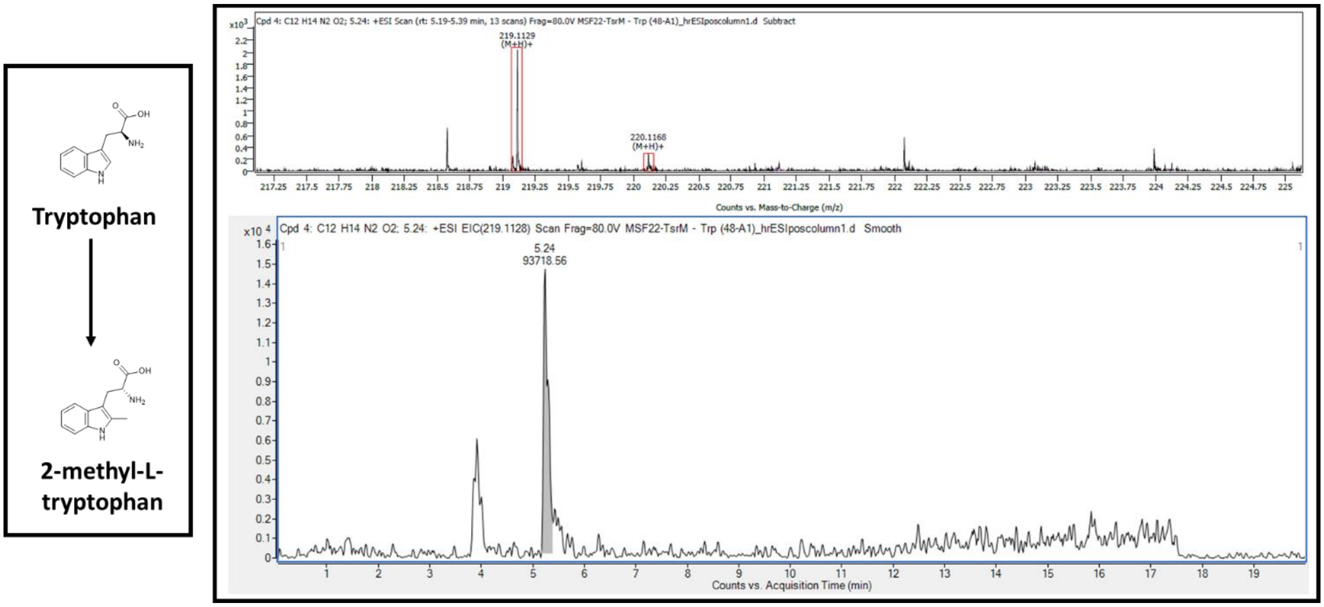

Supplementary Fig. 42. Analytical confirmation of formation of 2-methyl-L-tryptophan from tryptophan.

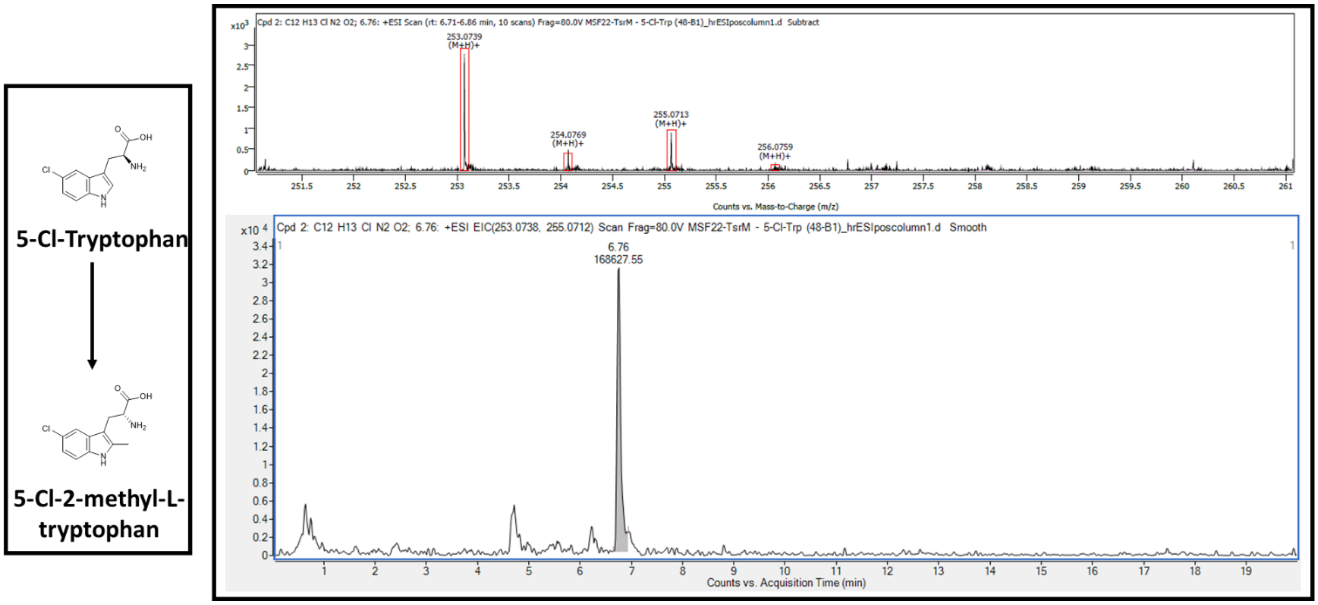

Supplementary Fig. 43. Analytical confirmation of formation of 5-Cl-2-methyl-L-tryptophan from 5-Cl-tryptophan.

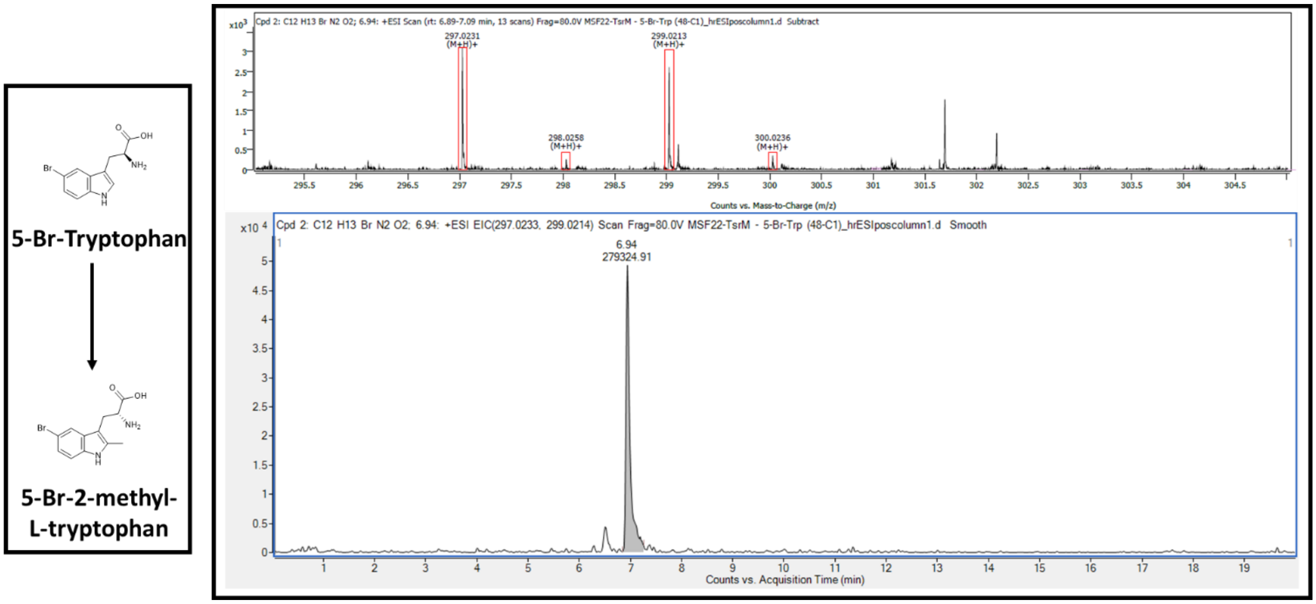

Supplementary Fig. 44. Analytical confirmation of formation of 5-Br-2-methyl-L-tryptophan from 5-Br-tryptophan.

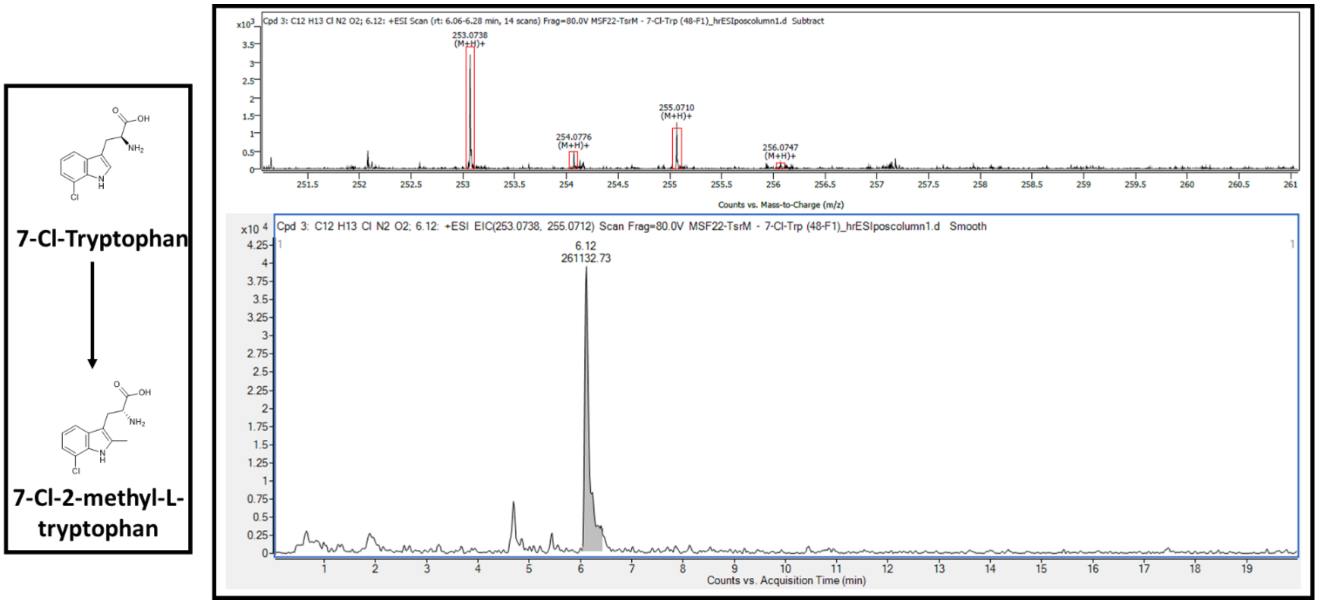

Supplementary Fig. 45. Analytical confirmation of formation of 7-Cl-2-methyl-L-tryptophan from 7-Cl-tryptophan.

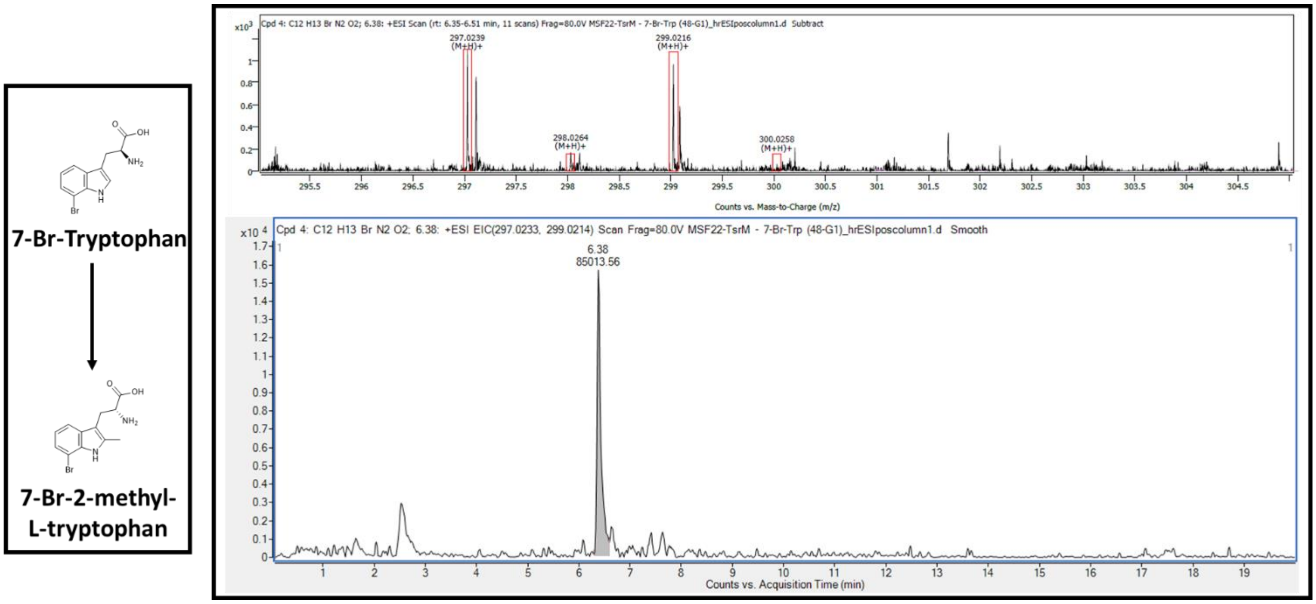

Supplementary Fig. 46. Analytical confirmation of formation of 7-Br-2-methyl-L-tryptophan from 7-Br-tryptophan.

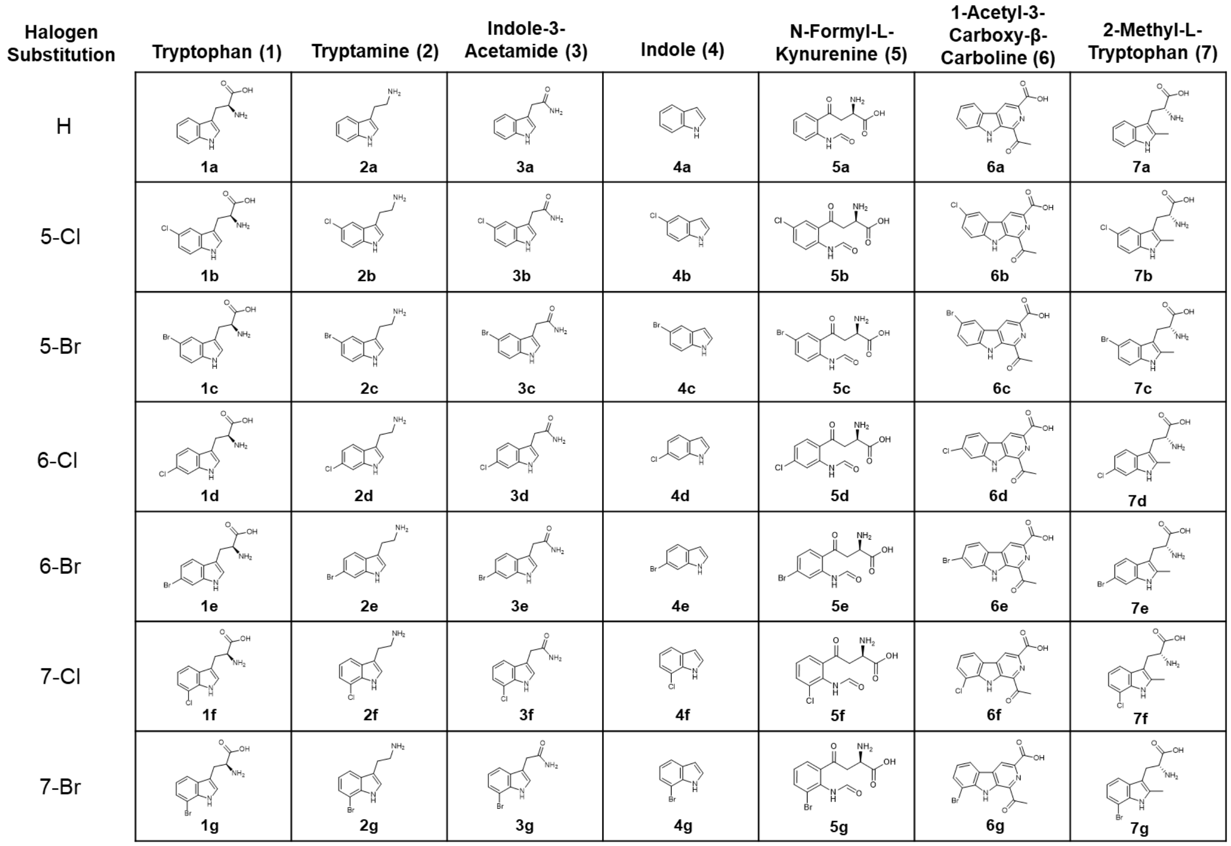

Supplementary Fig. 47. Overview of products investigated in this study. Molecule map containing all the theoretical molecules to be fed or produced from the six functioning downstream enzymes evaluated in this study, organized by functional group substitution (halogen) and base molecule. Molecule names are as follows, emphasizing the halogen first: tryptophan (1a), 5-chloro-tryptophan (1b), 5-bromo-tryptophan (1c), 6-chloro-tryptophan (1d), 6-bromo-tryptophan (1e), 7-chloro-tryptophan (1f), 7-bromo-tryptophan (1g), tryptamine (2a), 5-chloro-tryptamine (2b), 5-bromo-tryptamine (2c), 6-chloro-tryptamine (2d), 6-bromo-tryptamine (2e), 7-chloro-tryptamine (2f), 7-bromo-tryptamine (2g), indole-3-acetamide (3a), 5-chloro-indole-3-acetamide (3b), 5-bromo-indole-3-acetamide (3c), 6-chloro-indole-3-acetamide (3d), 6-bromo-indole-3-acetamide (3e), 7-chloro-indole-3-acetamide (3f), 7-bromo-indole-3-acetamide (3g), indole (4a), 5-chloro-indole (4b), 5-bromo-indole (4c), 6-chloro-indole (4d), 6-bromo-indole (4e), 7-chloro-indole (4f), 7-bromo-indole (4g), N-formyl-L-kynurenine (5a), 5-chloro-N-formyl-L-kynurenine (5b), 5-bromo-N-formyl-L-kynurenine (5c), 6-chloro-N-formyl-L-kynurenine (5d), 6-bromo-N-formyl-L-kynurenine (5e), 7-chloro-N-formyl-L-kynurenine (5f), 7-bromo-N-formyl-L-kynurenine (5g), 1-acetyl-3-carboxy-β-carboline (6a), 5-chloro-1-acetyl-3-carboxy-β-carboline (6b), 5-bromo-1-acetyl-3-carboxy-β-carboline (6c), 6-chloro-1-acetyl-3-carboxy-β-carboline (6d), 6-bromo-1-acetyl-3-carboxy-β-carboline (6e), 7-chloro-1-acetyl-3-carboxy-β-carboline (6f), 7-bromo-1-acetyl-3-carboxy-β-carboline (6g), 2-methyl-L-tryptophan (7a), 5-chloro-2-methyl-L-tryptophan (7b), 5-bromo-2-methyl-L-tryptophan (7c), 6-chloro-2-methyl-L-tryptophan (7d), 6-bromo-2-methyl-L-tryptophan (7e), 7-chloro-2-methyl-L-tryptophan (7f), 7-bromo-2-methyl-L-tryptophan (7g).

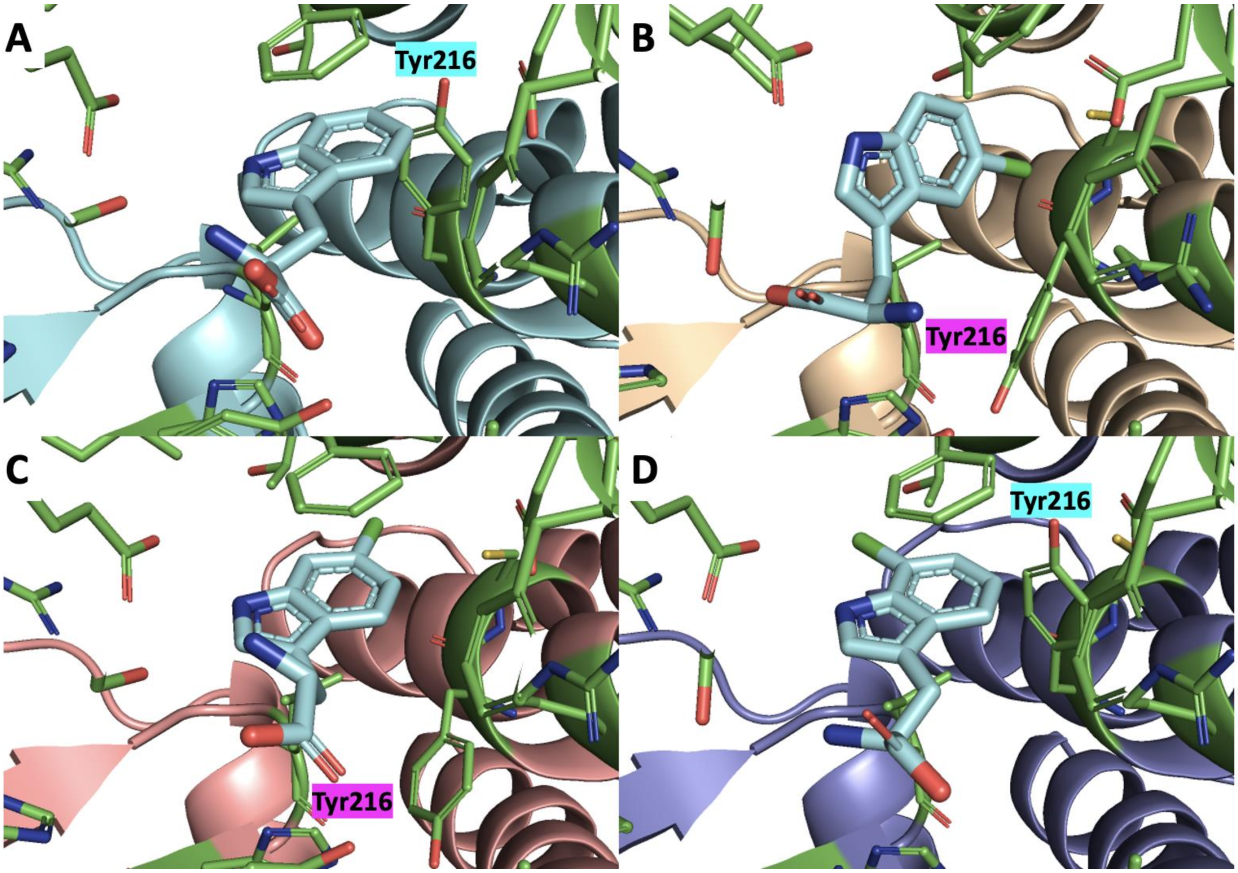

Supplementary Fig. 48. McbB docking study with chloro-tryptophans. McbB bound with A) L-tryptophan B) 5-Cl-Tryptophan C) 6-Cl-Tryptophan D) 7-Cl-Tryptophan. Tyr216 residues are labeled, with pink and blue text denoting down and up confirmations, respectively.

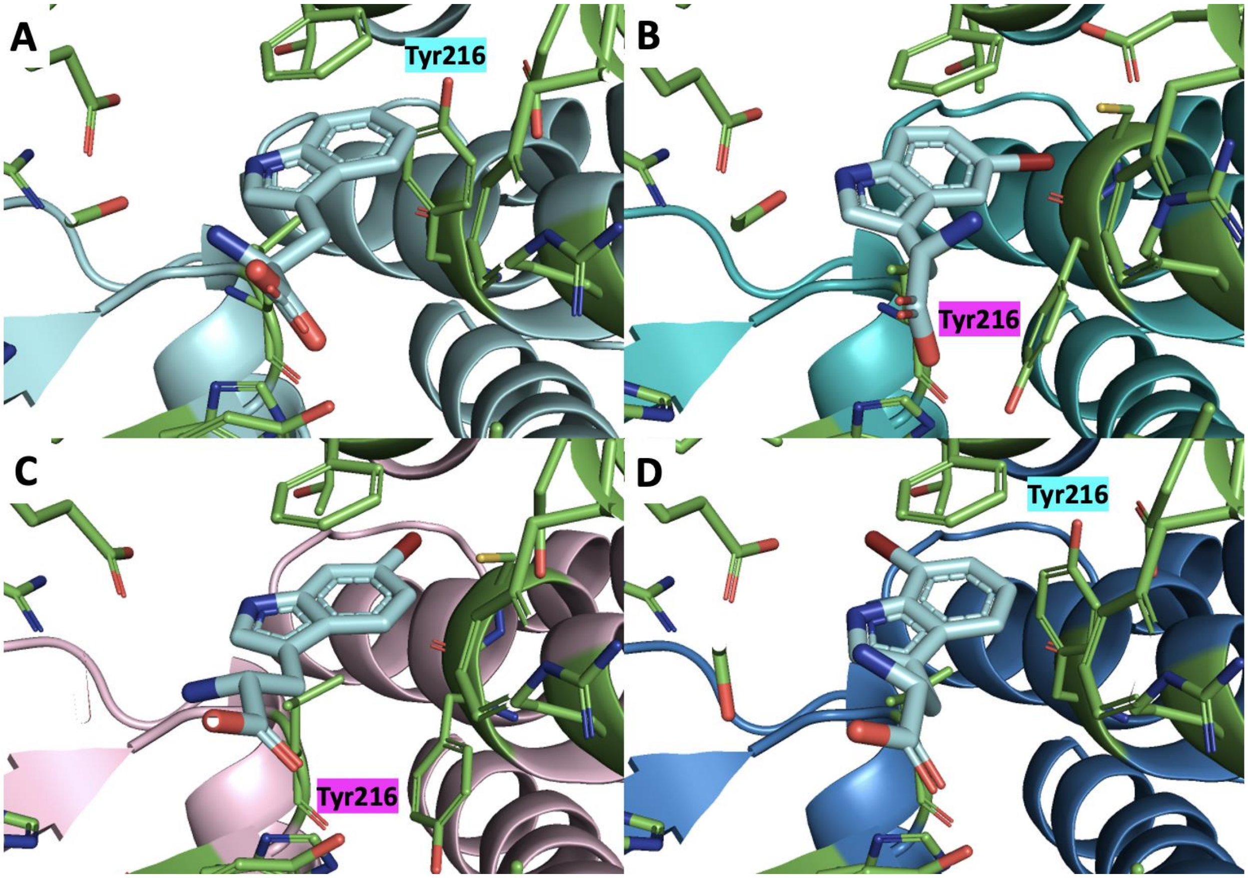

Supplementary Fig. 49. McbB docking study with bromo-tryptophans. McbB bound with A) L-tryptophan B) 5-Br-Tryptophan C) 6-Br-Tryptophan D) 7-Br-Tryptophan. Tyr216 residues are labeled, with pink and blue text denoting down and up confirmations, respectively.

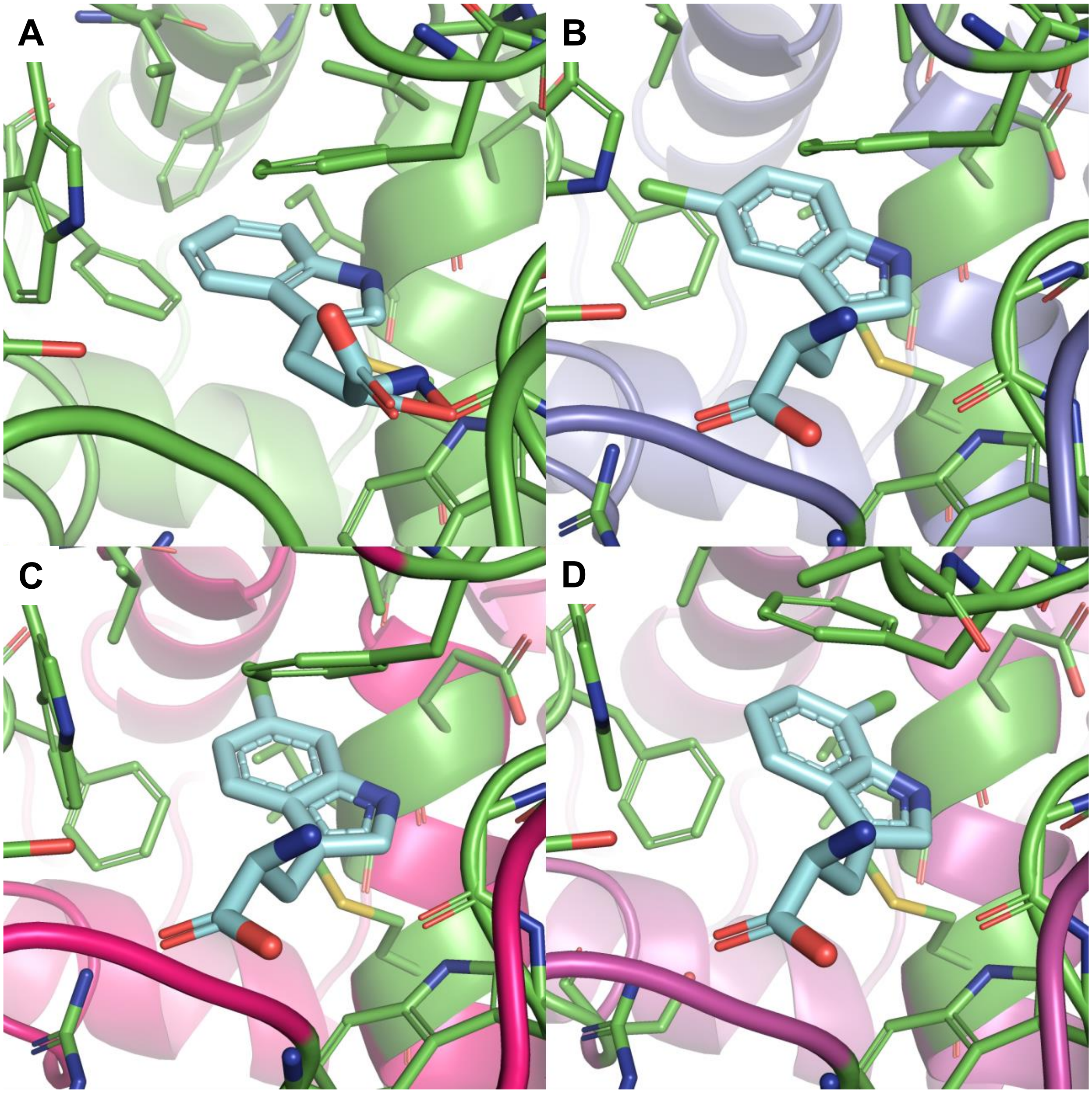

Supplementary Fig. 50. iaaM docking study with chloro-tryptophans. iaaM bound with A) L-tryptophan B) 5-Cl-Tryptophan C) 6-Cl-Tryptophan D) 7-Cl-Tryptophan

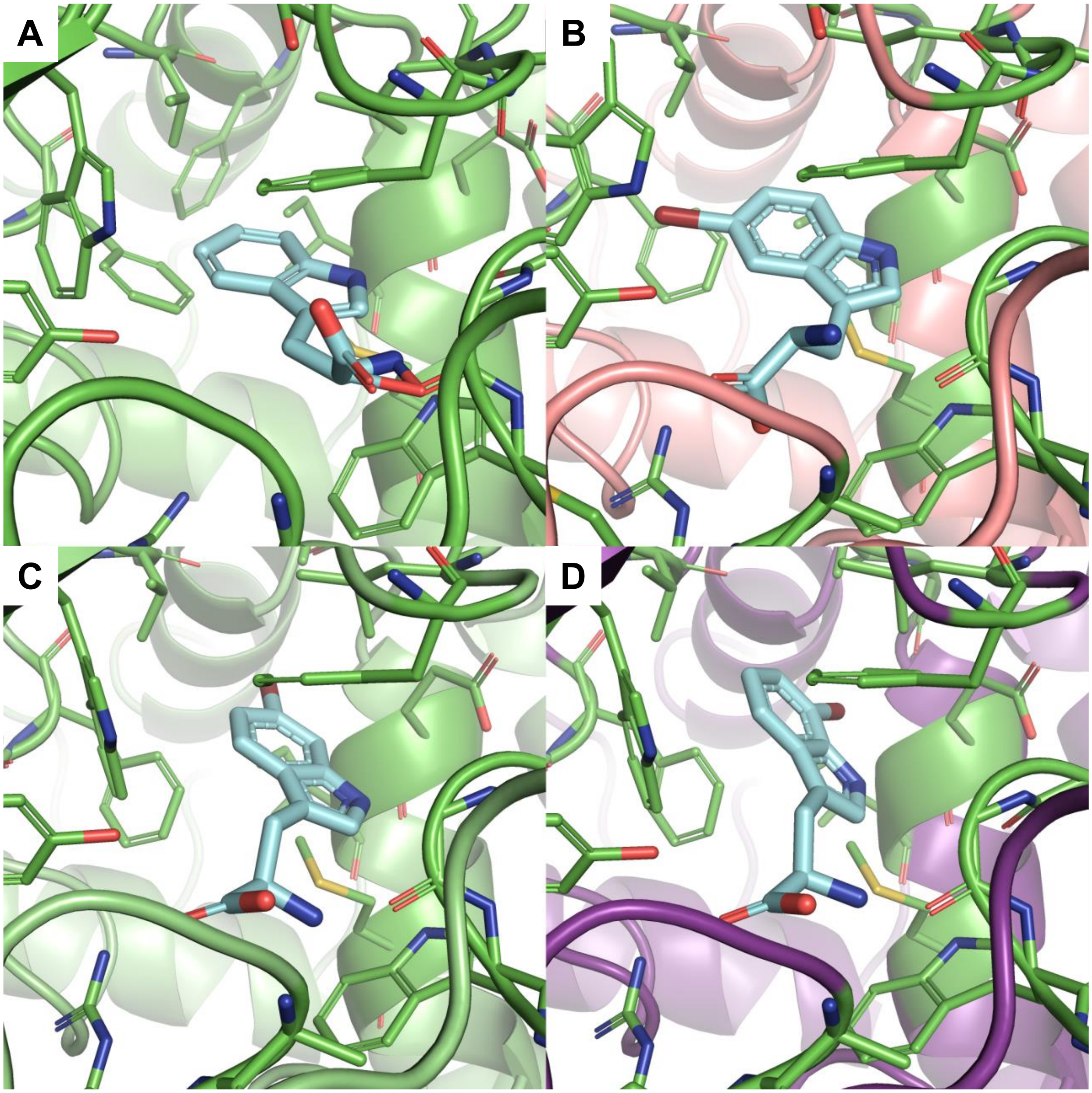

Supplementary Fig. 51. iaaM docking study with bromo-tryptophans. iaaM bound with A) L-tryptophan B) 5-Br-Tryptophan C) 6-Br-Tryptophan D) 7-Br-Tryptophan

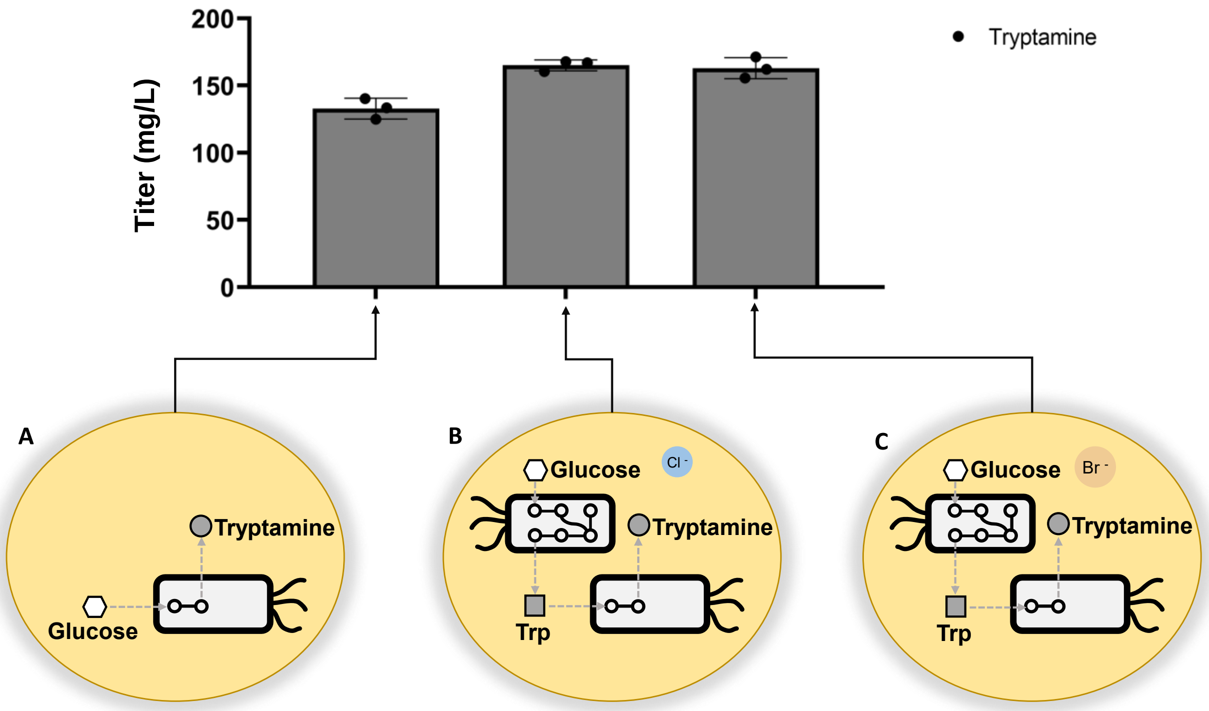

Supplementary Fig. 52. Tryptamine quantification in halo-tryptamine co-culture. Production of tryptamine using A) only the downstream module cell, B) upstream plus downstream co-culture in chloride-focused media, and C) upstream plus downstream co-culture in bromide-focused media. Error bars represent S.E. of 3 biological triplicates. Source data are provided as a Source Data file.

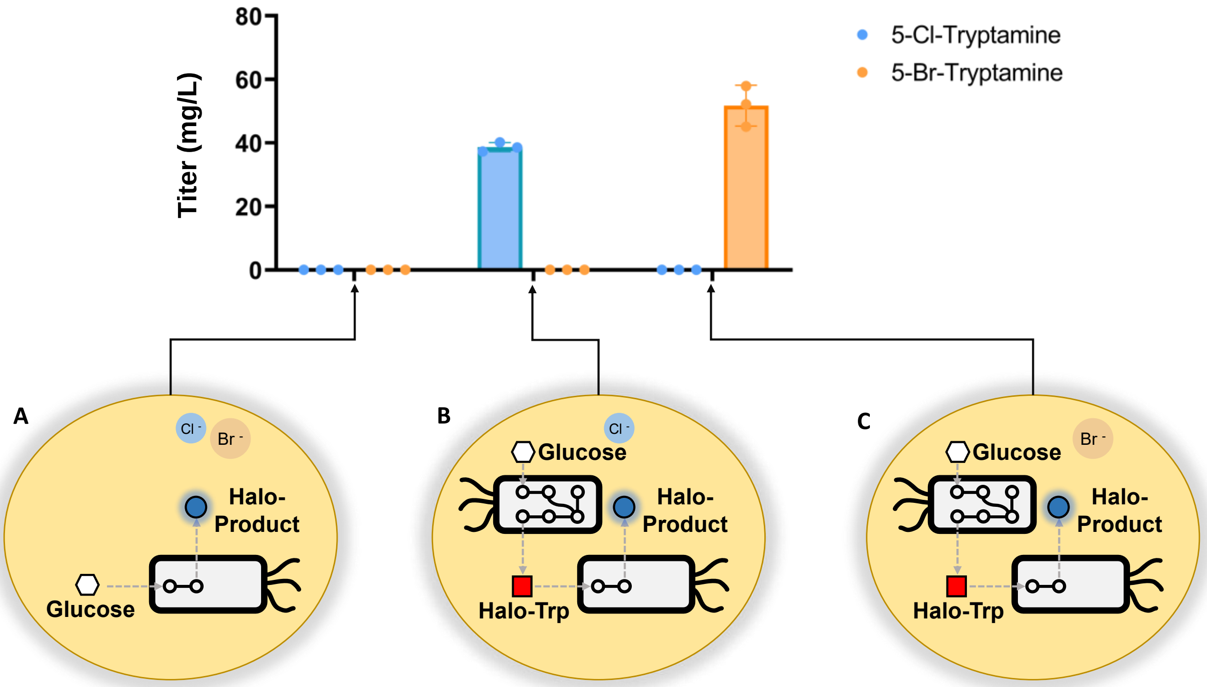

Supplementary Fig. 53. Halo-tryptamine quantification in halo-tryptamine co-culture. Production of 5-Cl-tryptamine and 5-Br-tryptamine using A) only the downstream module cell, B) upstream plus downstream co-culture in chloride-focused media, and C) upstream plus downstream co-culture in bromide-focused media. Error bars represent S.E. of 3 biological triplicates. Source data are provided as a Source Data file.

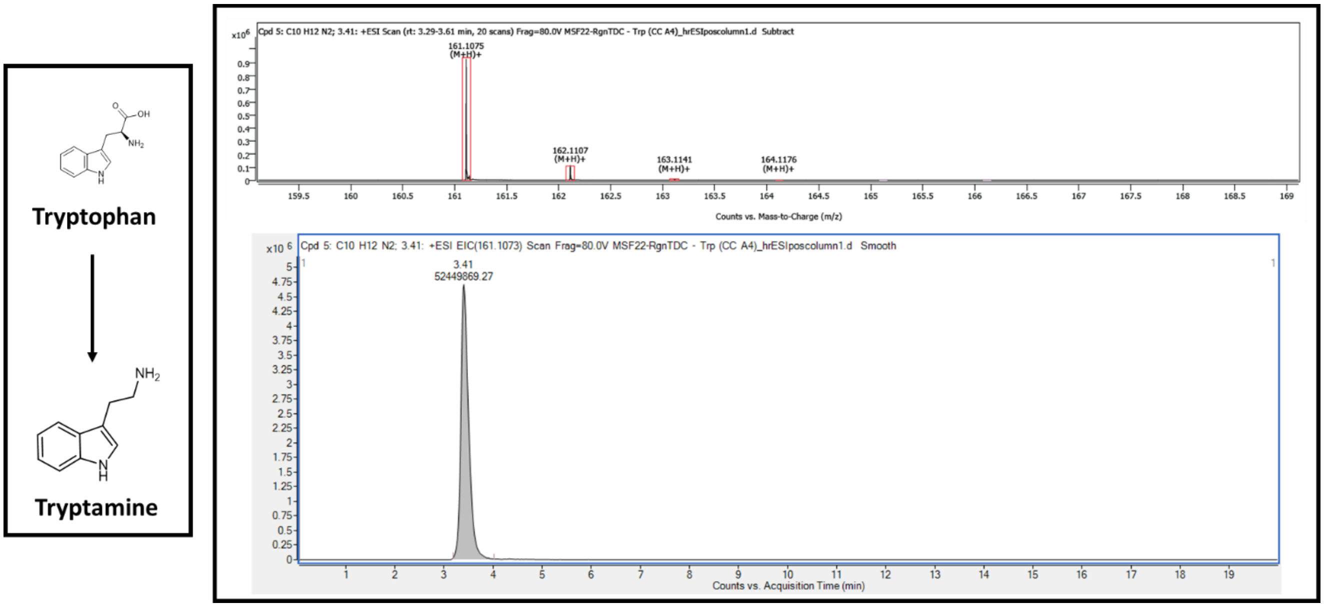

Supplementary Fig. 54. Analytical confirmation of *de novo* formation of tryptamine from glucose via coculture.

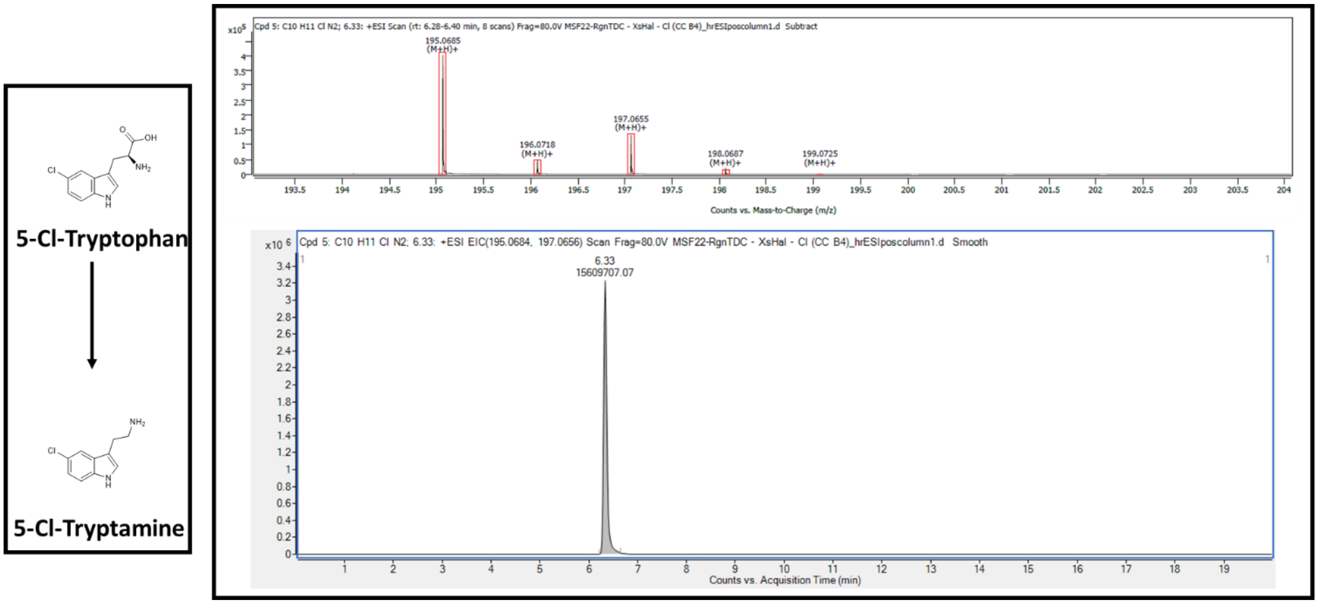

Supplementary Fig. 55. Analytical confirmation of *de novo* formation of 5-Cl-tryptamine from glucose via coculture.

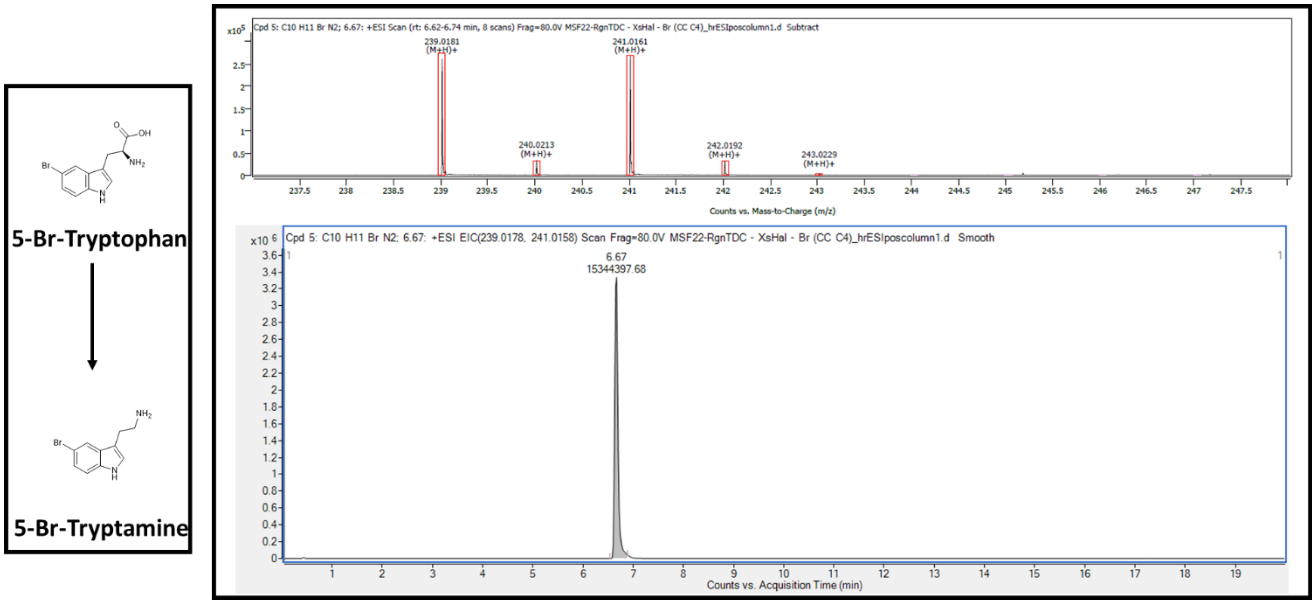

Supplementary Fig. 56. Analytical confirmation of *de novo* formation of 5-Br-tryptamine from glucose via coculture.

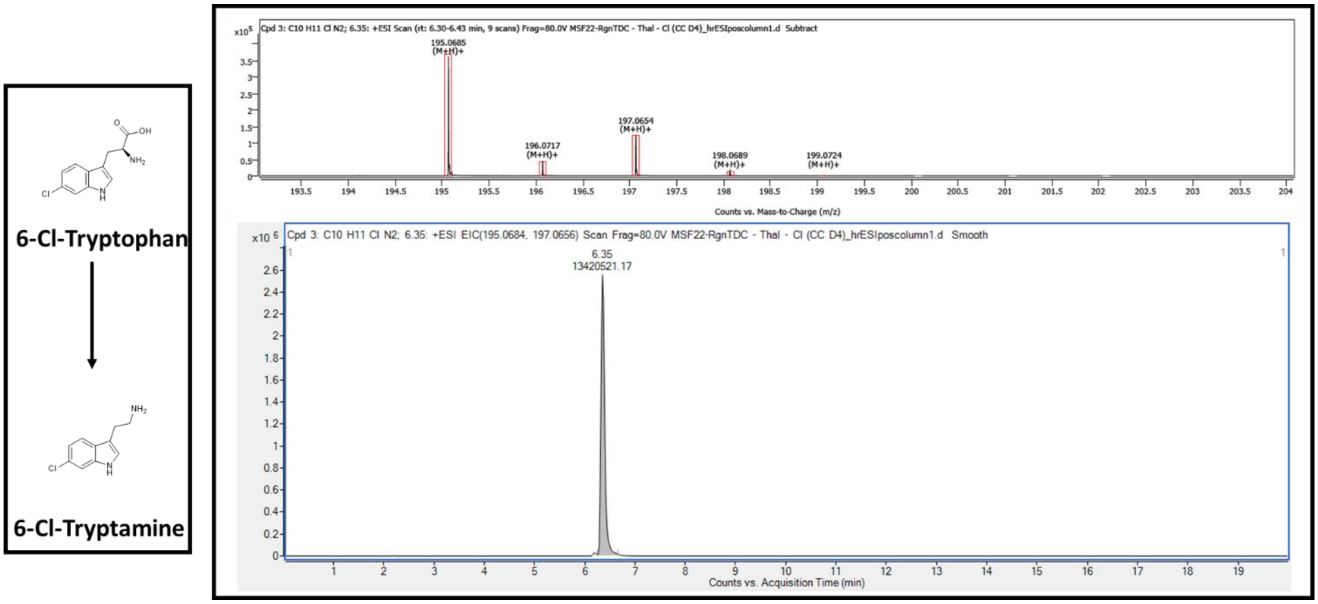

Supplementary Fig. 57. Analytical confirmation of *de novo* formation of 6-Cl-tryptamine from glucose via coculture.

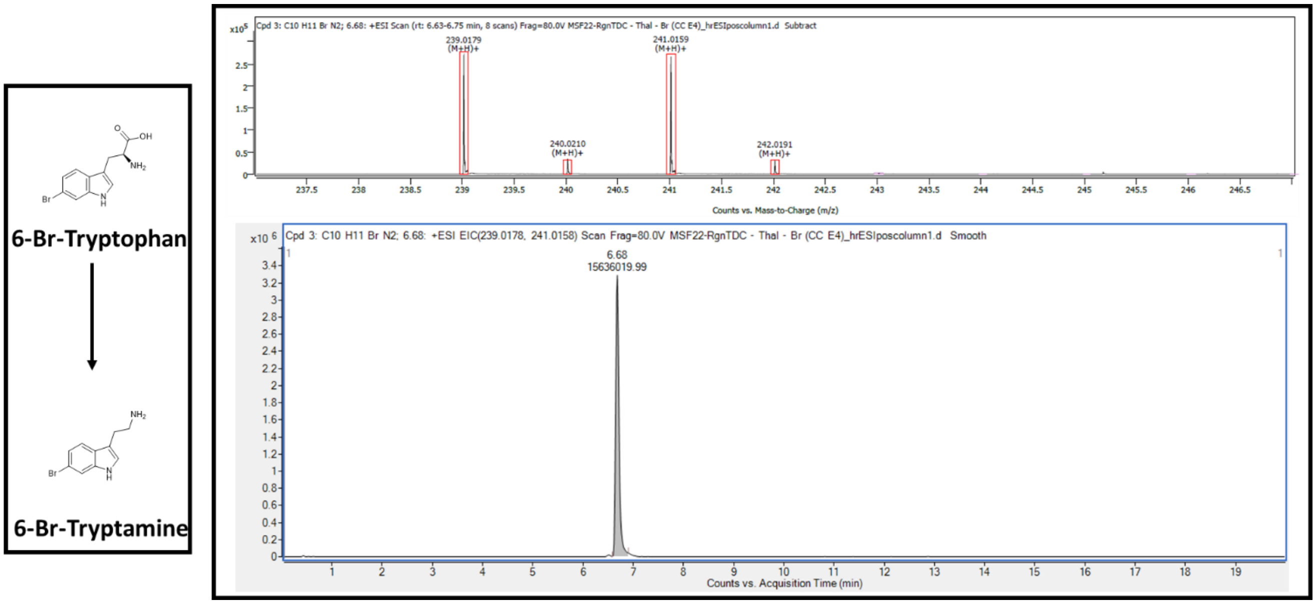

Supplementary Fig. 58. Analytical confirmation of *de novo* formation of 6-Br-tryptamine from glucose via coculture.

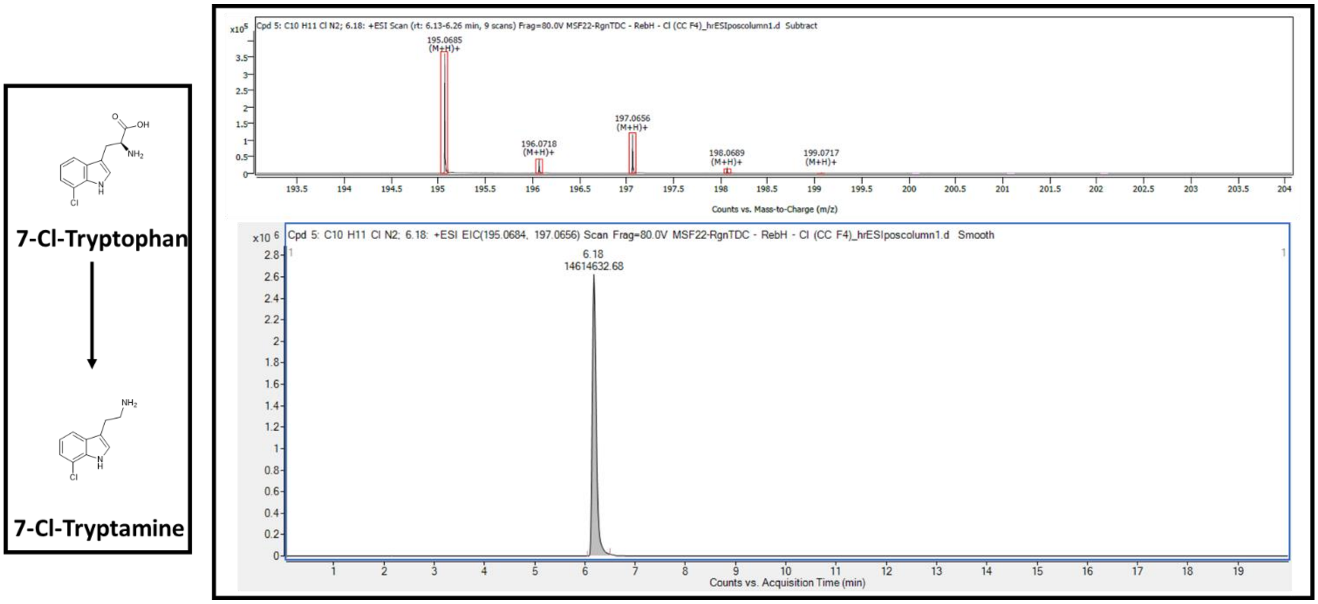

Supplementary Fig. 59. Analytical confirmation of *de novo* formation of 7-Cl-tryptamine from glucose via coculture.

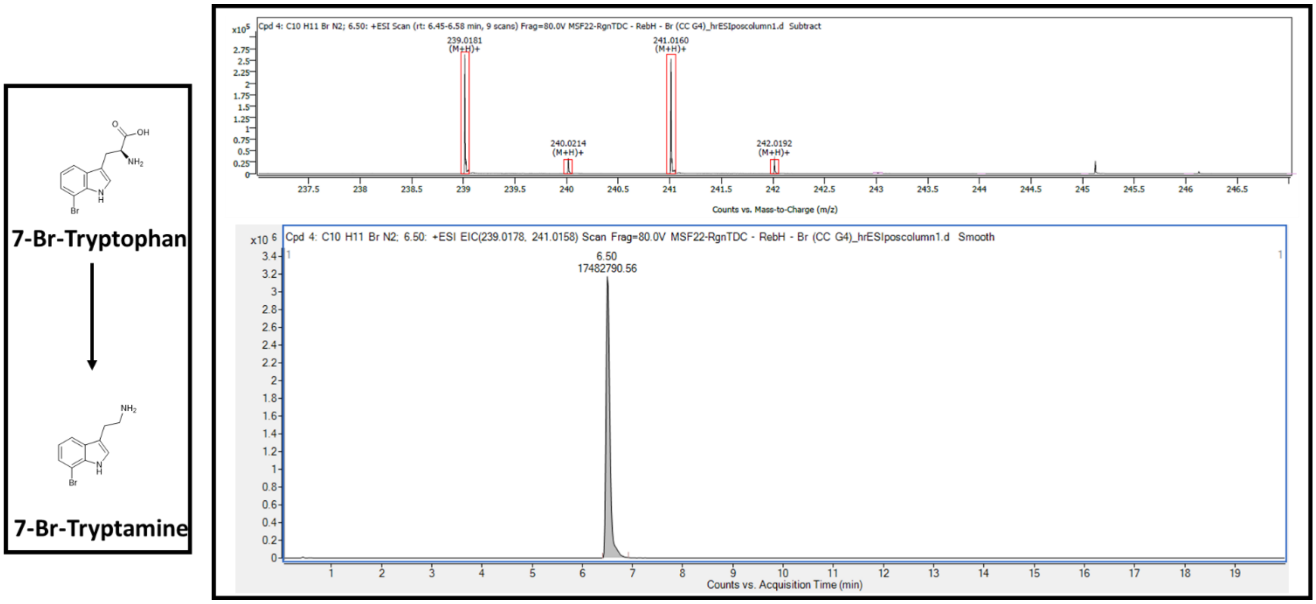

Supplementary Fig. 60. Analytical confirmation of *de novo* formation of 7-Br-tryptamine from glucose via coculture.

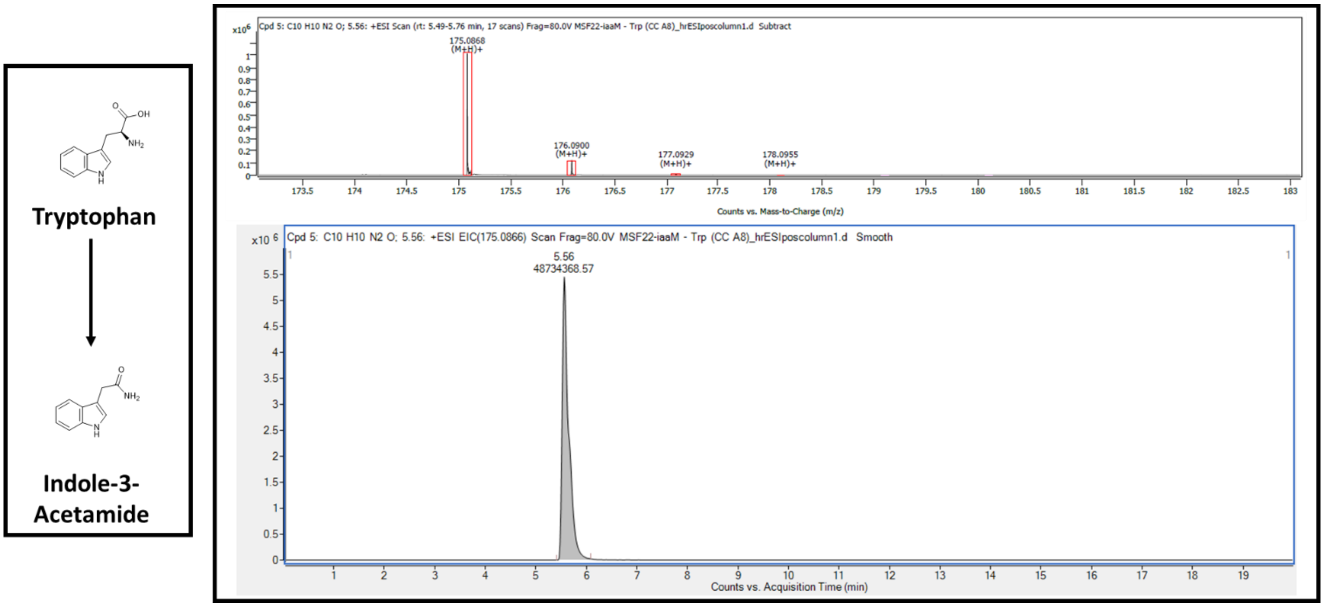

Supplementary Fig. 61. Analytical confirmation of *de novo* formation of indole-3-acetamide from glucose via coculture.

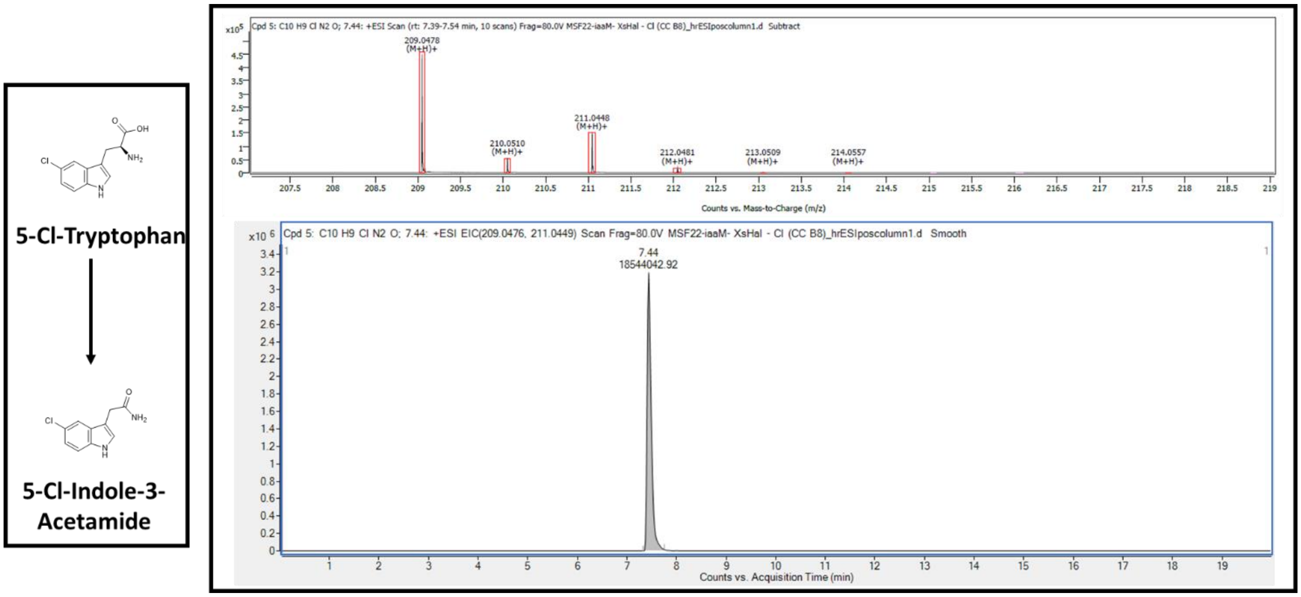

Supplementary Fig. 62. Analytical confirmation of *de novo* formation of 5-Cl-indole-3-acetamide from glucose via coculture.

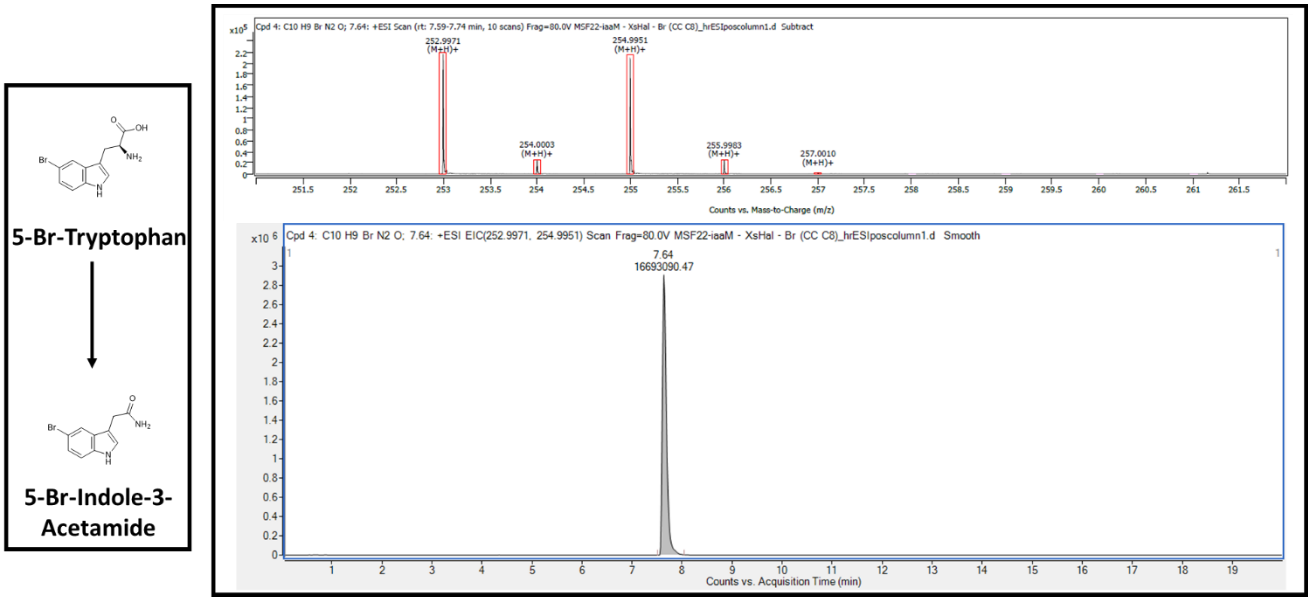

Supplementary Fig. 63. Analytical confirmation of *de novo* formation of 5-Br-indole-3-acetamide from glucose via coculture.

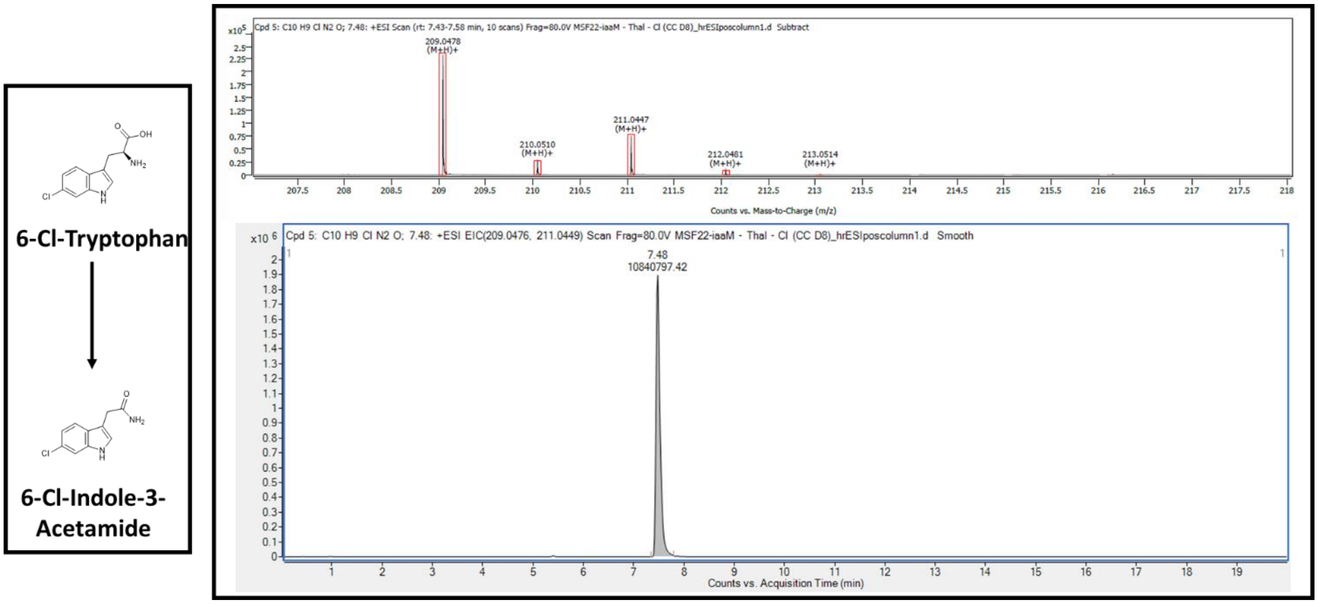

Supplementary Fig. 64. Analytical confirmation of *de novo* formation of 6-Cl-indole-3-acetamide from glucose via coculture.

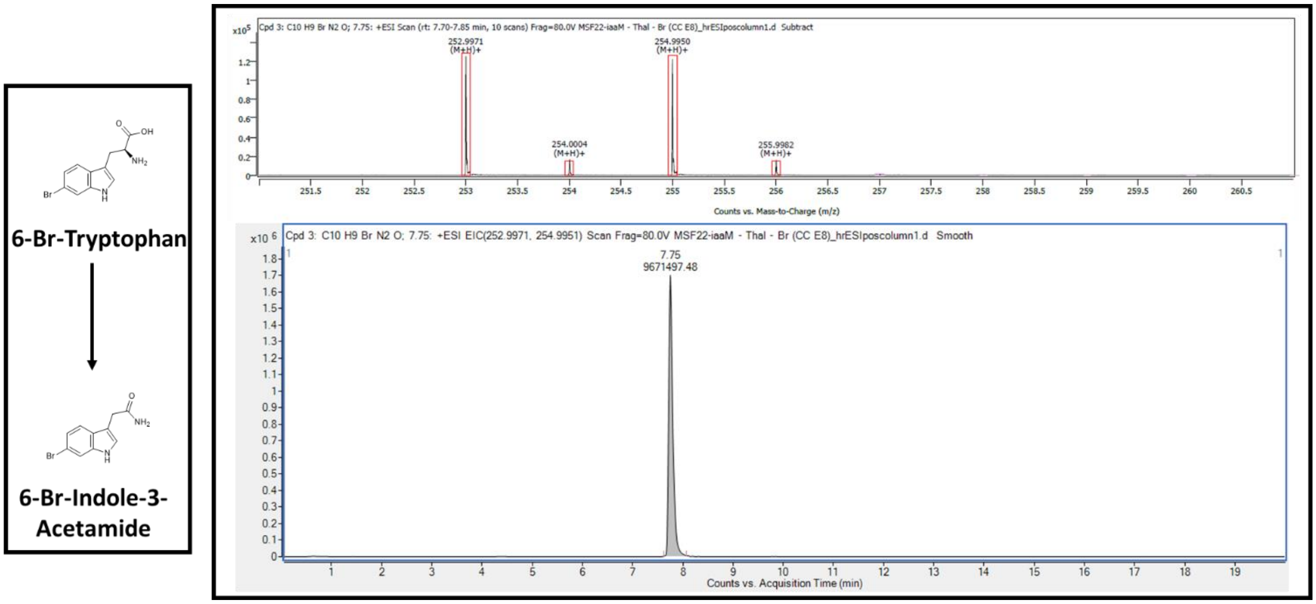

Supplementary Fig. 65. Analytical confirmation of *de novo* formation of 6-Br-indole-3-acetamide from glucose via coculture.

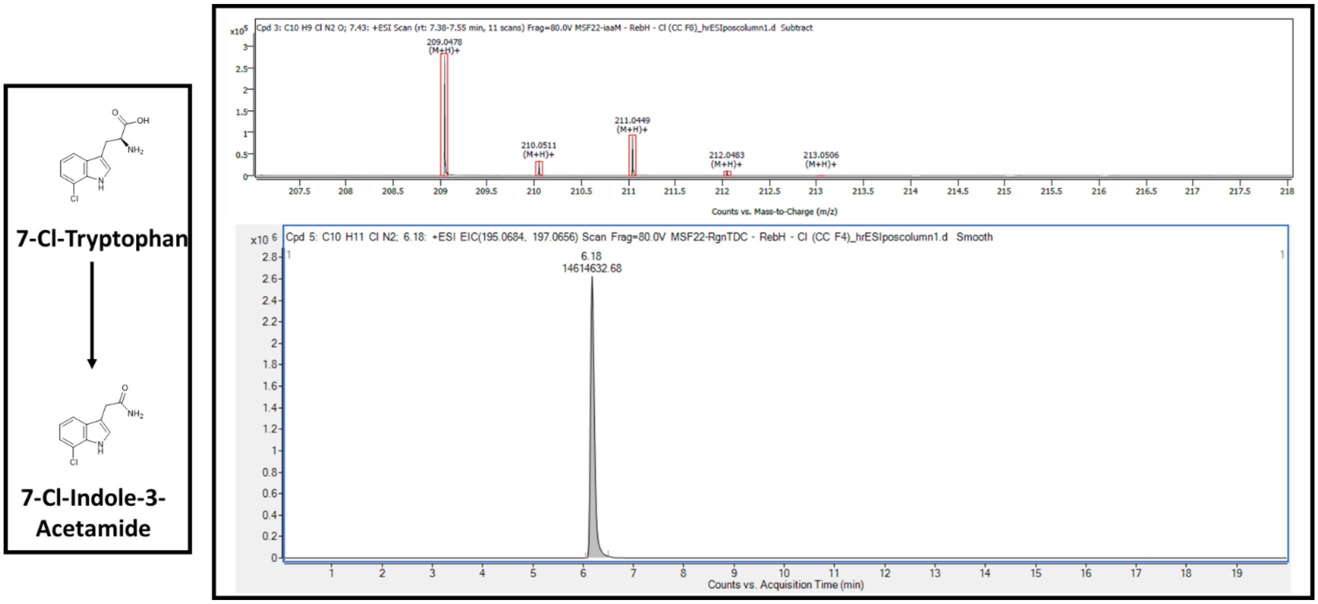

Supplementary Fig. 66. Analytical confirmation of *de novo* formation of 7-Cl-indole-3-acetamide from glucose via coculture.

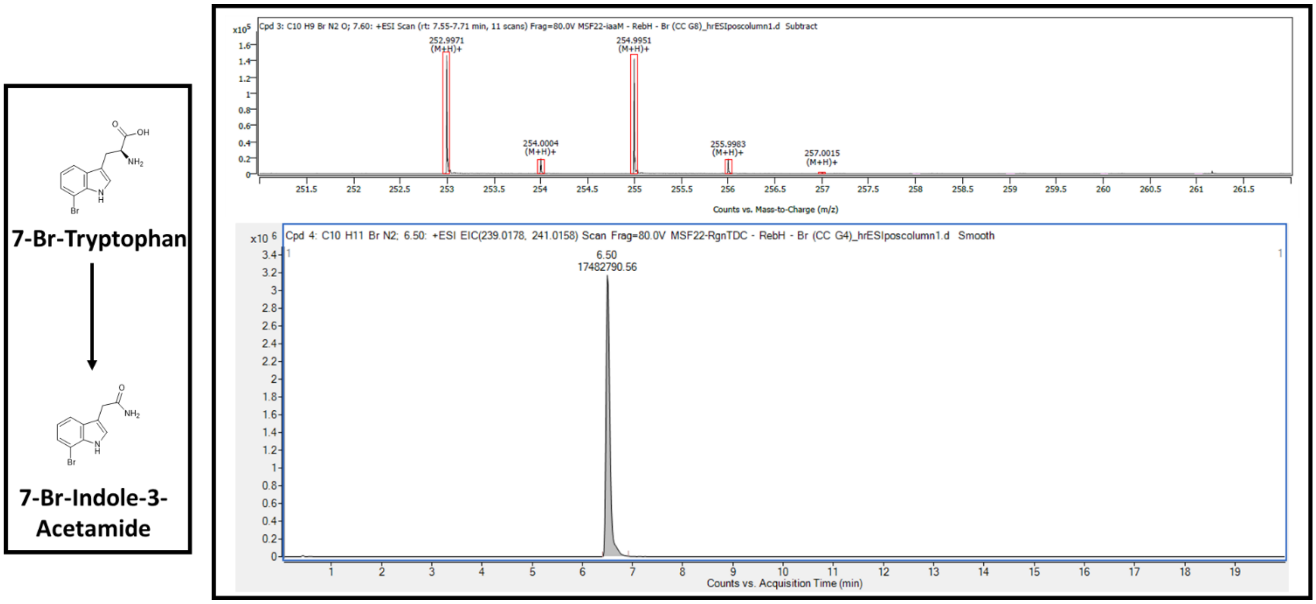

Supplementary Fig. 67. Analytical confirmation of *de novo* formation of 7-Br-indole-3-acetamide from glucose via coculture.

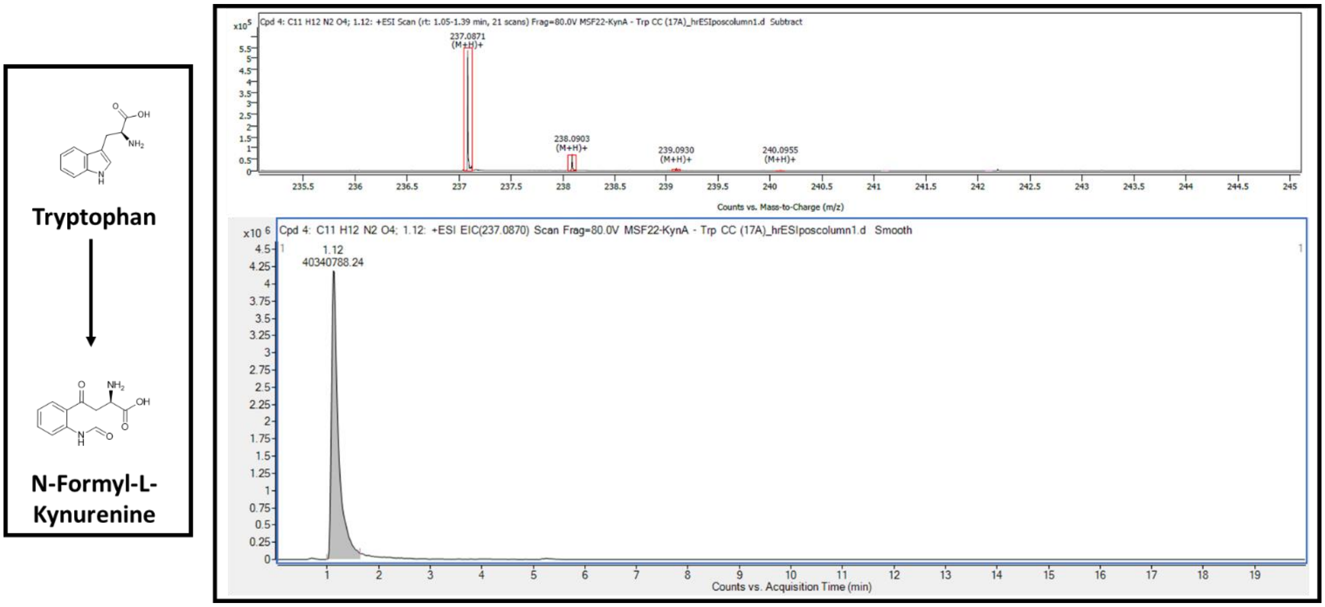

Supplementary Fig. 68. Analytical confirmation of *de novo* formation of N-formyl-L-kynurenine from glucose via coculture.

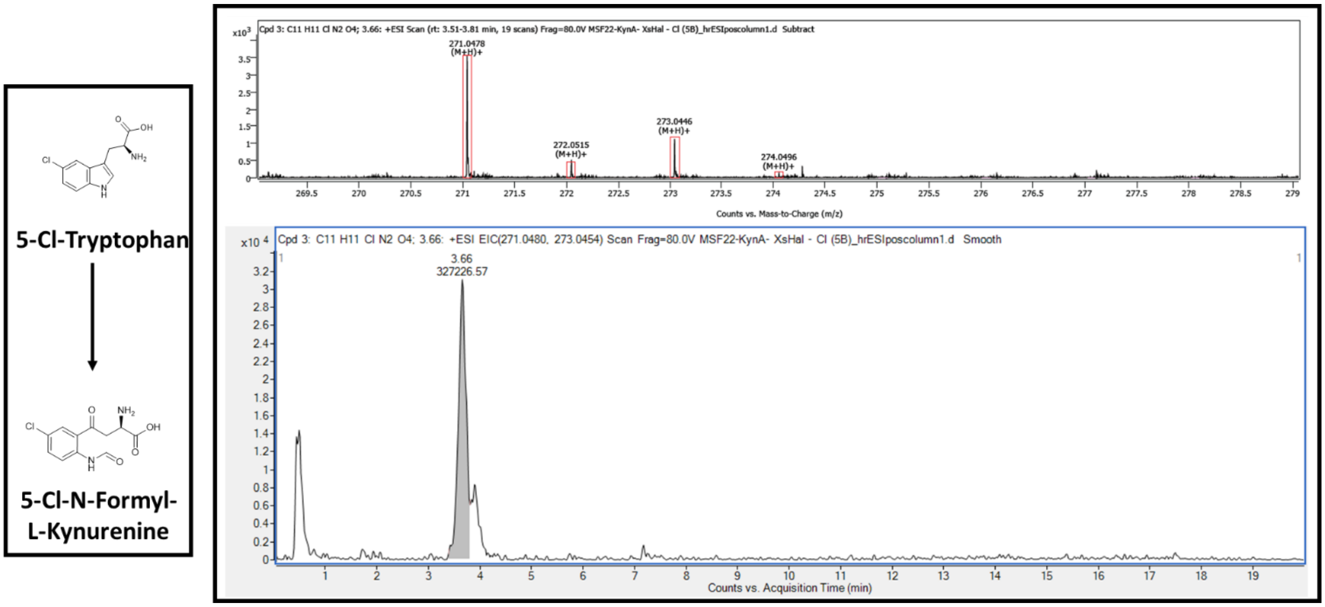

Supplementary Fig. 69. Analytical confirmation of *de novo* formation of 5-Cl-N-formyl-L-kynurenine from glucose via coculture.

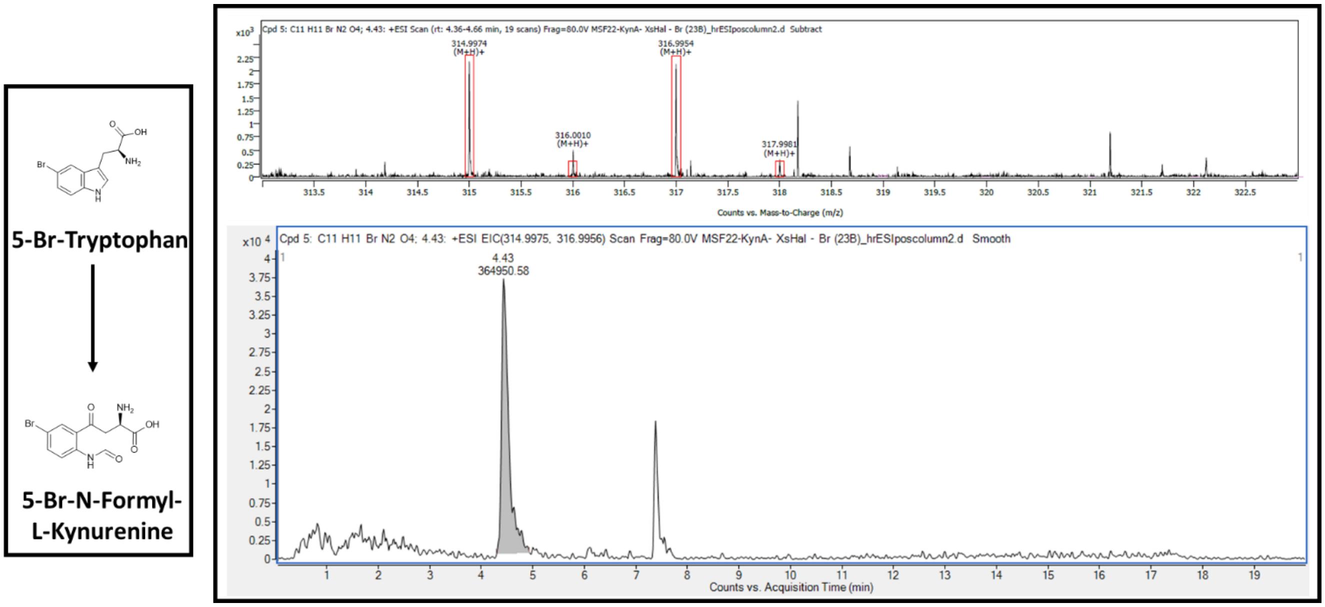

Supplementary Fig. 70. Analytical confirmation of *de novo* formation of 5-Br-N-formyl-L-kynurenine from glucose via coculture.

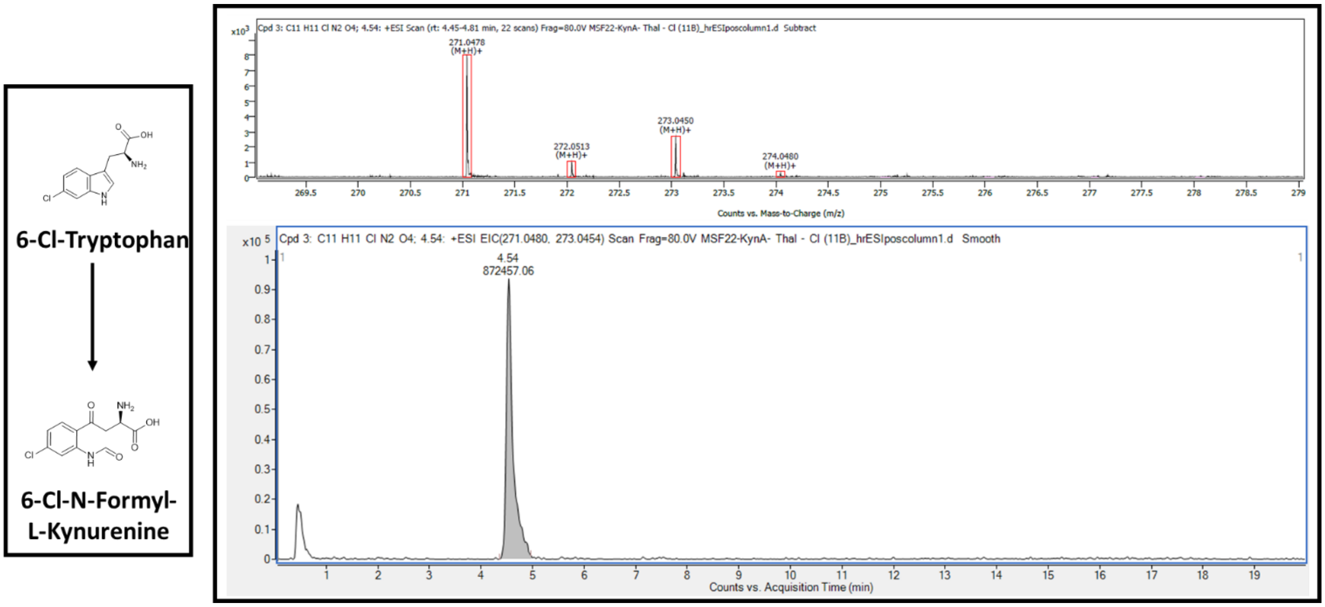

Supplementary Fig. 71. Analytical confirmation of *de novo* formation of 6-Cl-N-formyl-L-kynurenine from glucose via coculture.

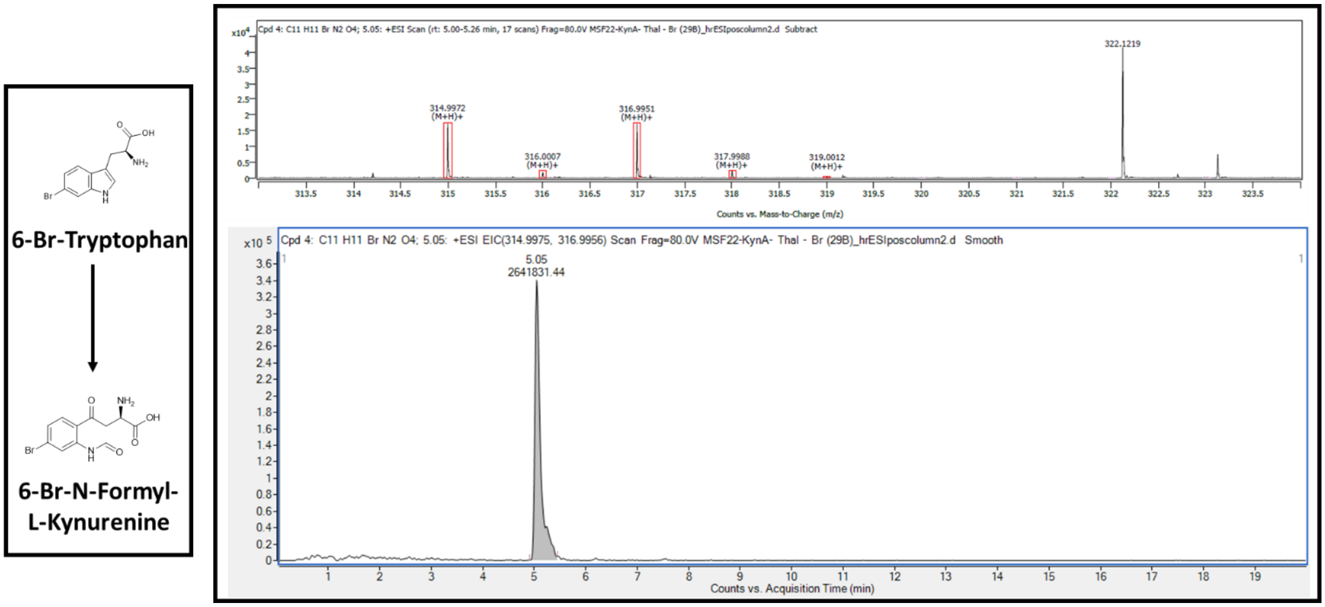

Supplementary Fig. 72. Analytical confirmation of *de novo* formation of 6-Br-N-formyl-L-kynurenine from glucose via coculture.

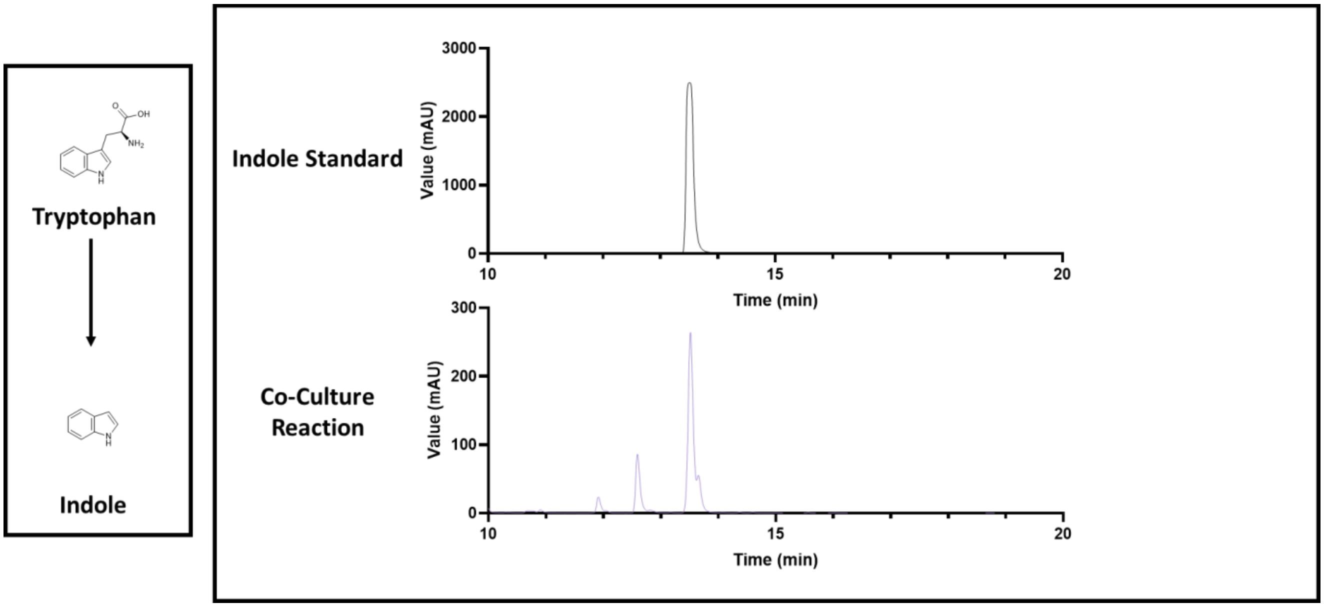

Supplementary Fig. 73. Analytical confirmation of *de novo* formation of indole from glucose via coculture using HPLC.

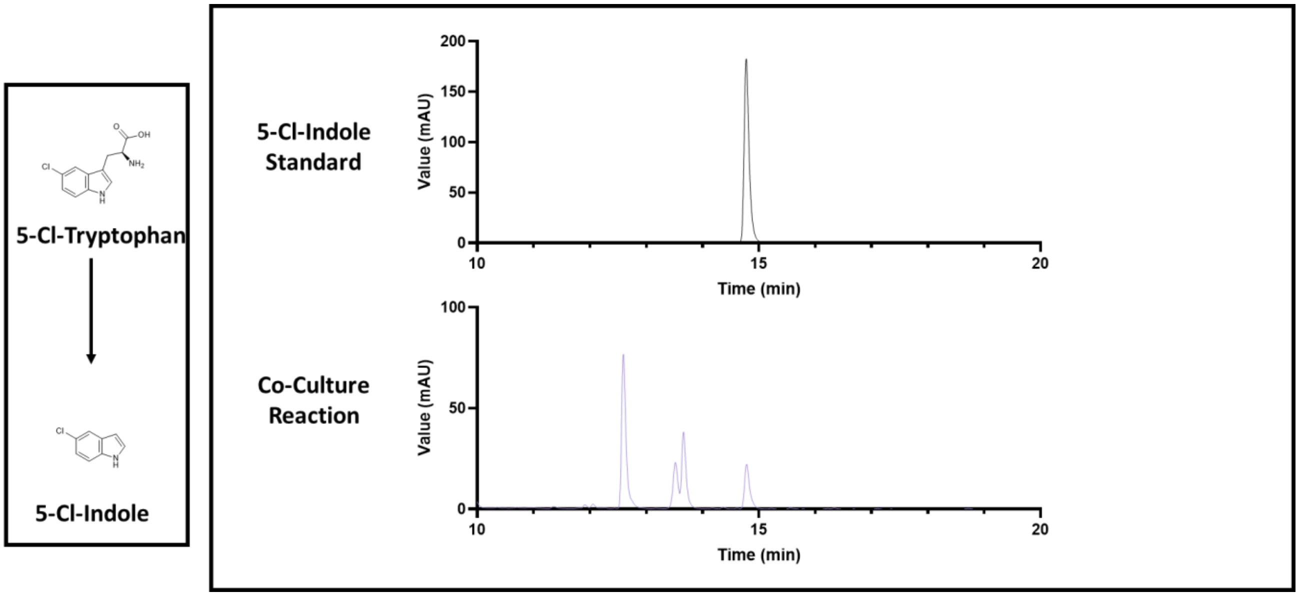

Supplementary Fig. 74. Analytical confirmation of *de novo* formation of 5-Cl-indole from glucose via coculture using HPLC.

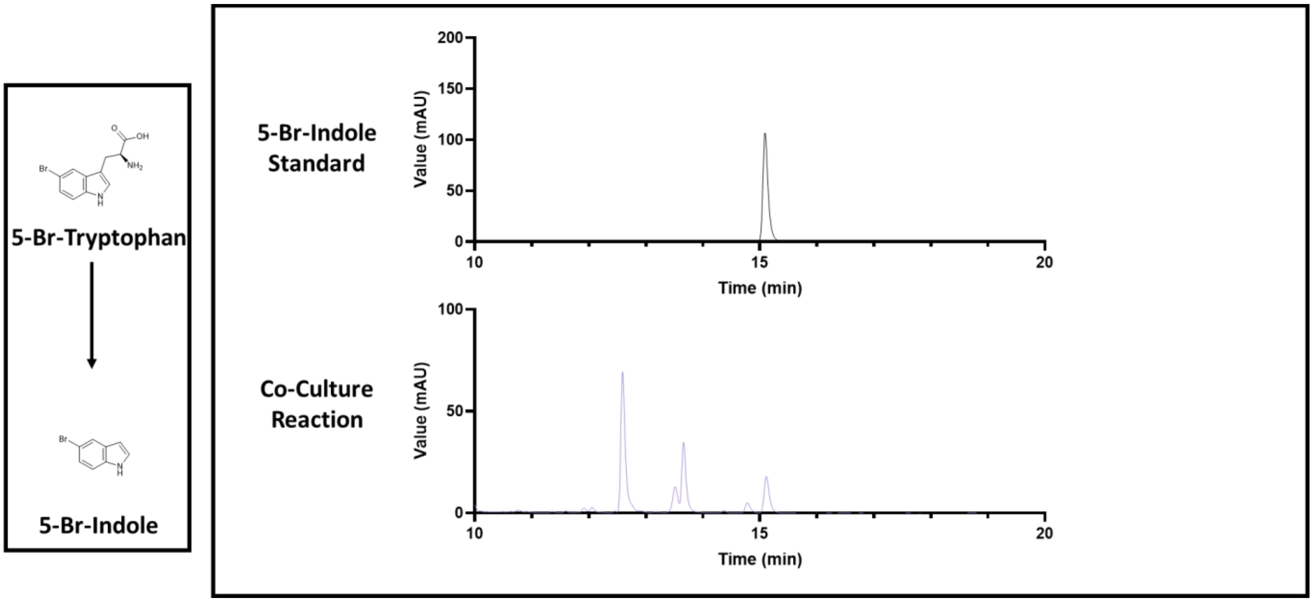

Supplementary Fig. 75. Analytical confirmation of *de novo* formation of 5-Br-indole from glucose via coculture using HPLC.

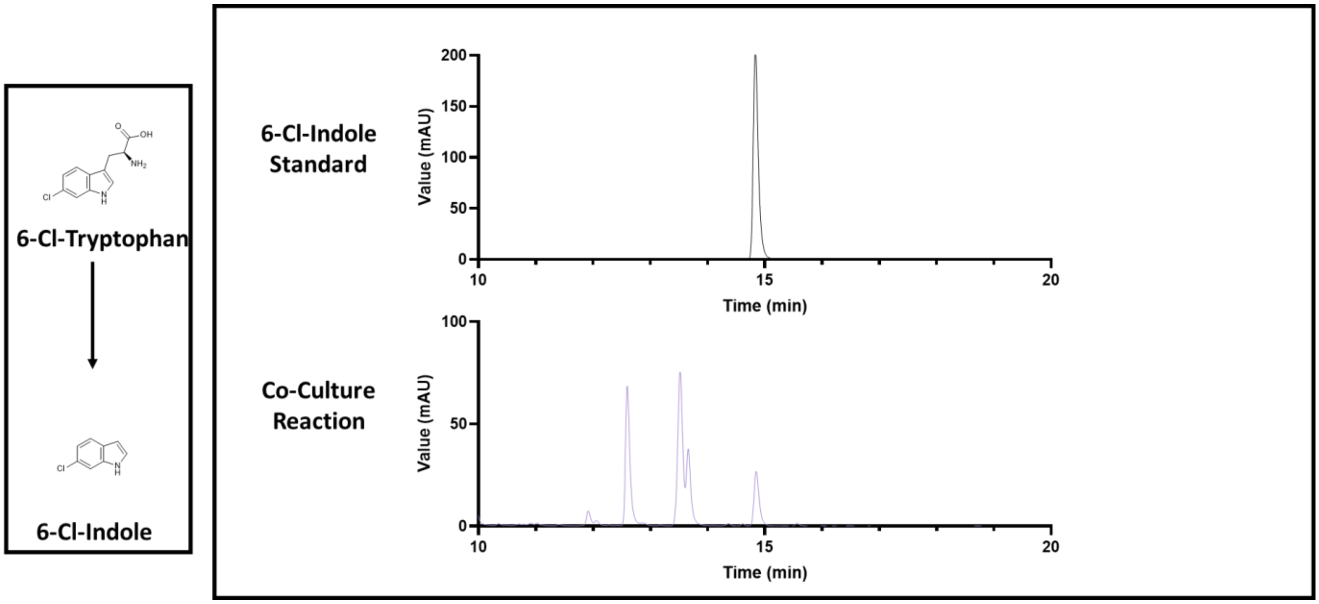

Supplementary Fig. 76. Analytical confirmation of *de novo* formation of 6-Cl-indole from glucose via coculture using HPLC.

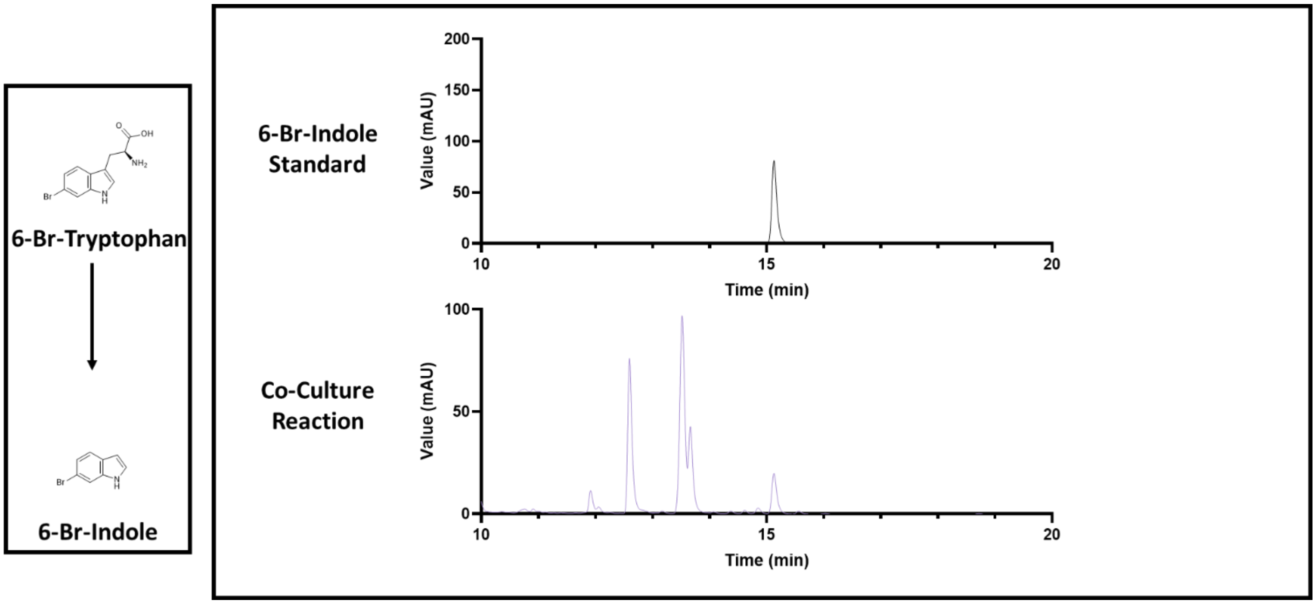

Supplementary Fig. 77. Analytical confirmation of *de novo* formation of 6-Br-indole from glucose via coculture using HPLC.

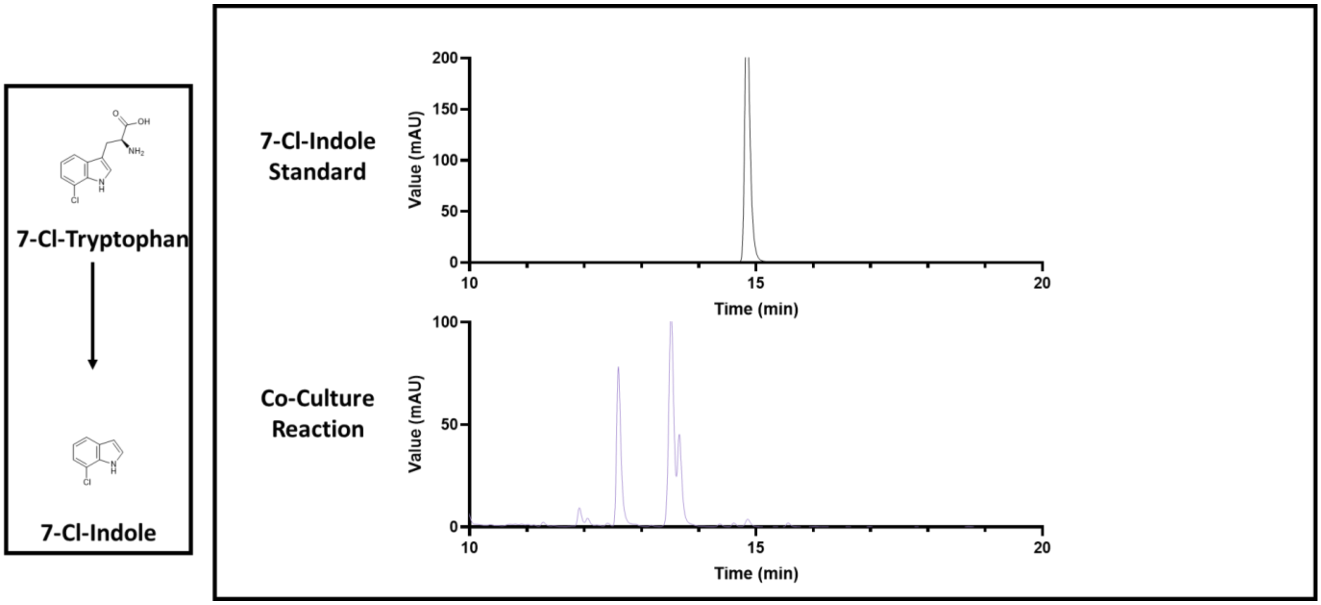

Supplementary Fig. 78. Analytical confirmation of *de novo* formation of 7-Cl-indole from glucose via coculture using HPLC.

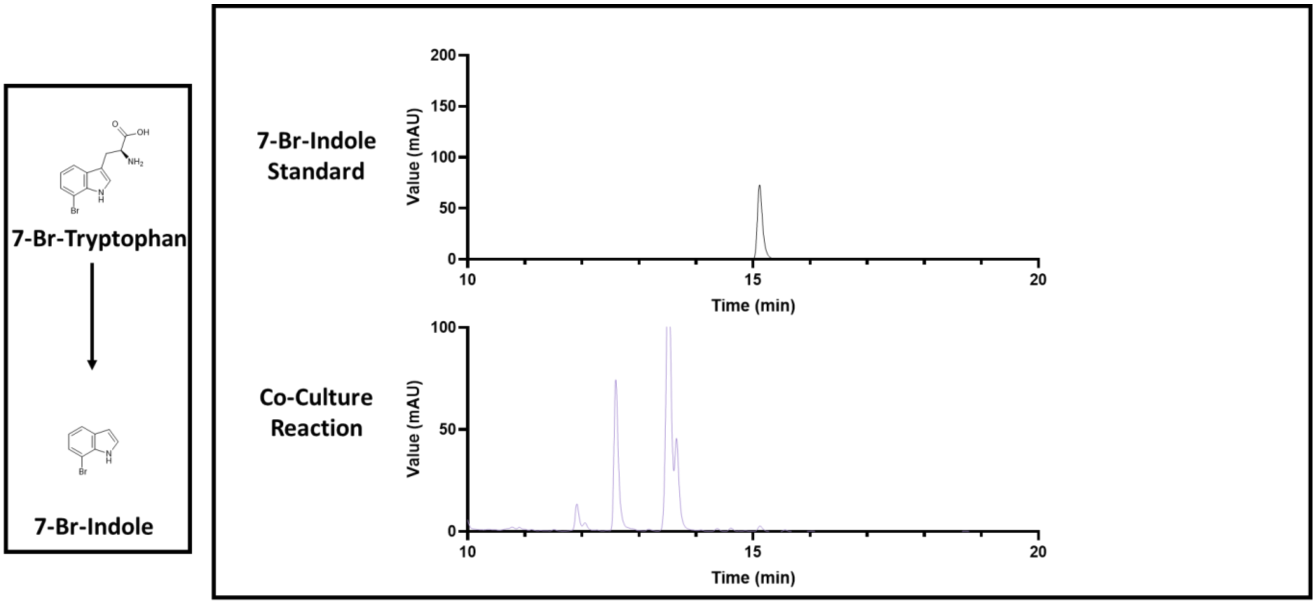

Supplementary Fig. 79. Analytical confirmation of *de novo* formation of 7-Br-indole from glucose via coculture using HPLC.

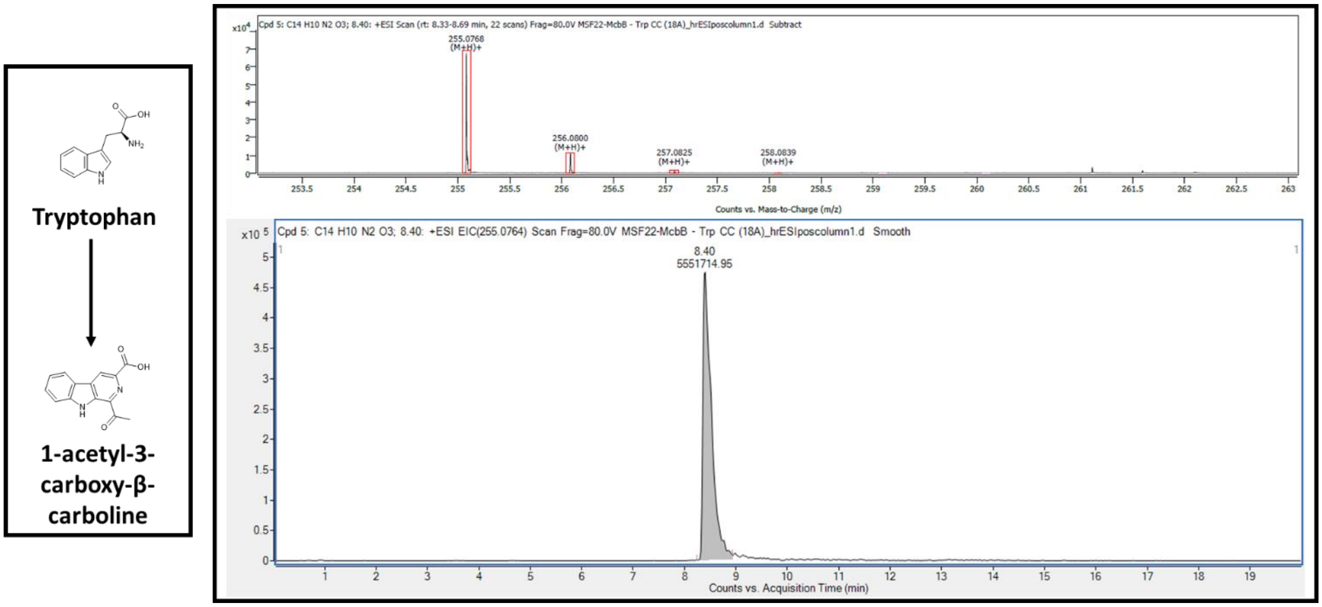

Supplementary Fig. 80. Analytical confirmation of *de novo* formation of 1-acetyl-3-carboxyl β-carboline from glucose via coculture.

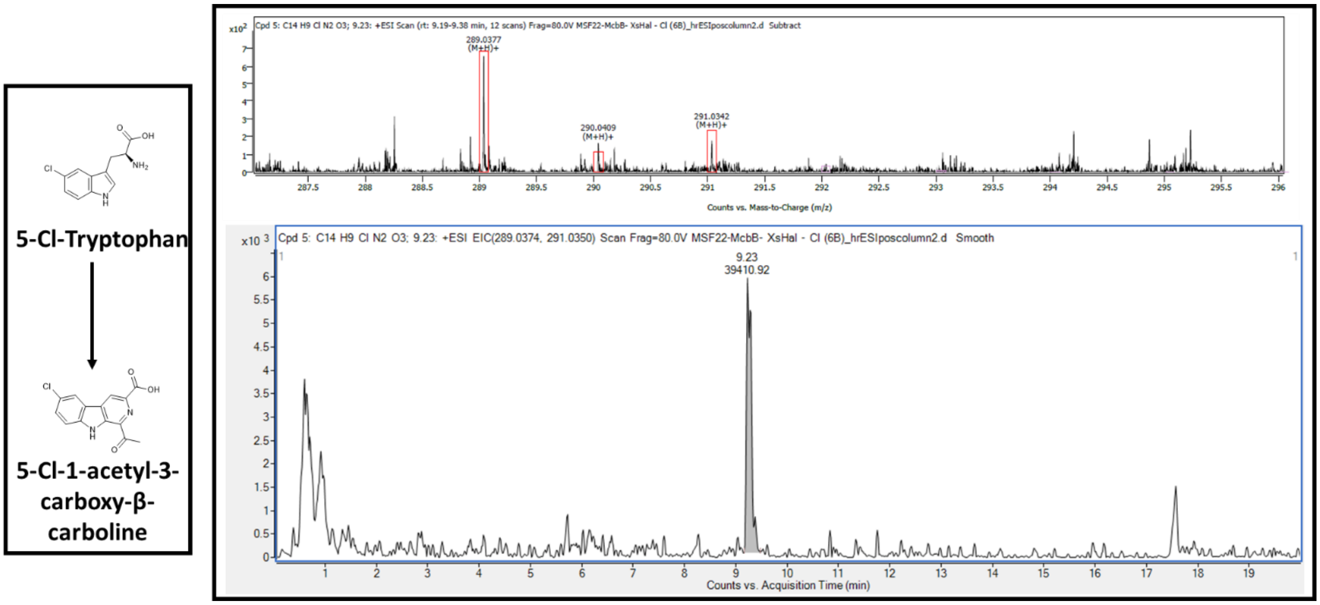

Supplementary Fig. 81. Analytical confirmation of *de novo* formation of 5-Cl-1-acetyl-3-carboxyl β-carboline from glucose via coculture.

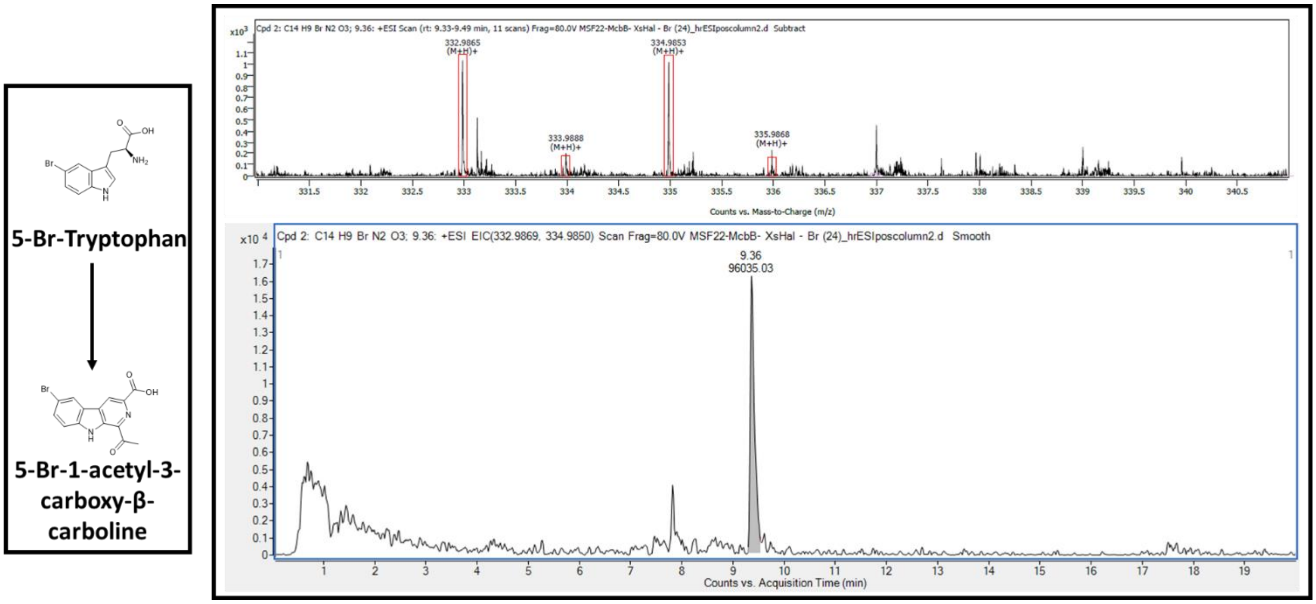

Supplementary Fig. 82. Analytical confirmation of *de novo* formation of 5-Br-1-acetyl-3-carboxyl β-carboline from glucose via coculture.

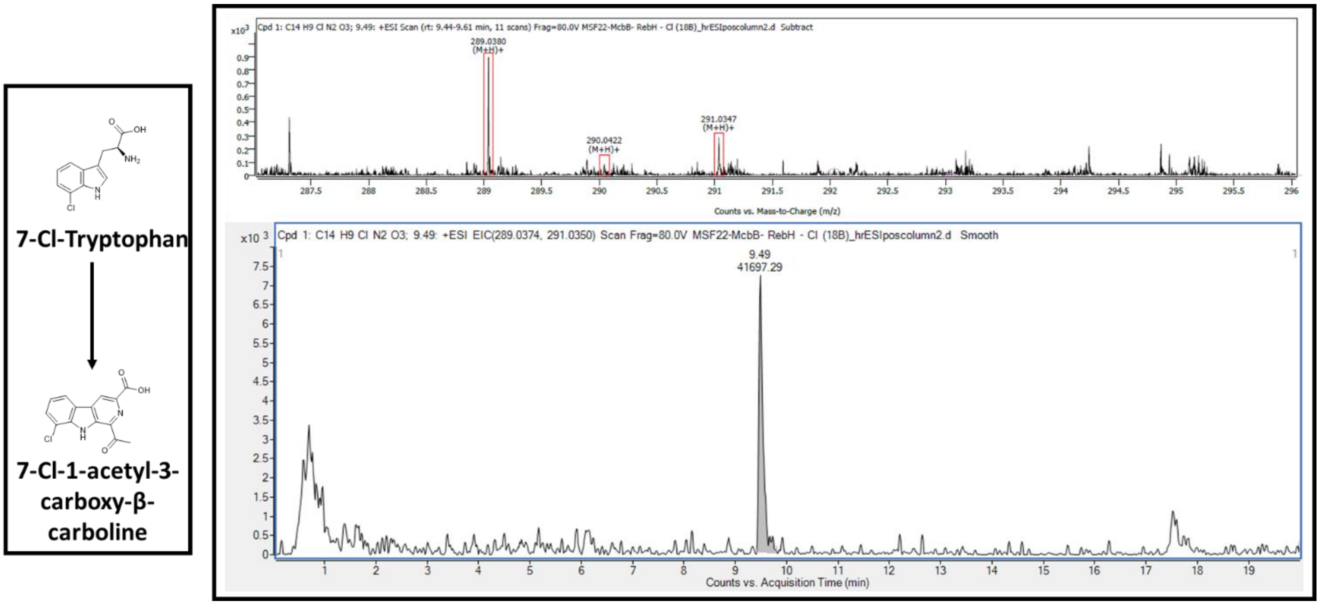

Supplementary Fig. 83. Analytical confirmation of *de novo* formation of 7-Cl-1-acetyl-3-carboxyl β-carboline from glucose via coculture.

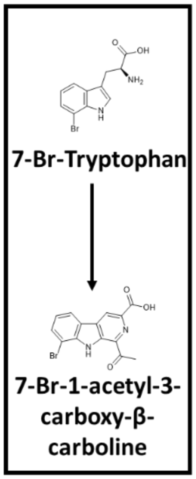

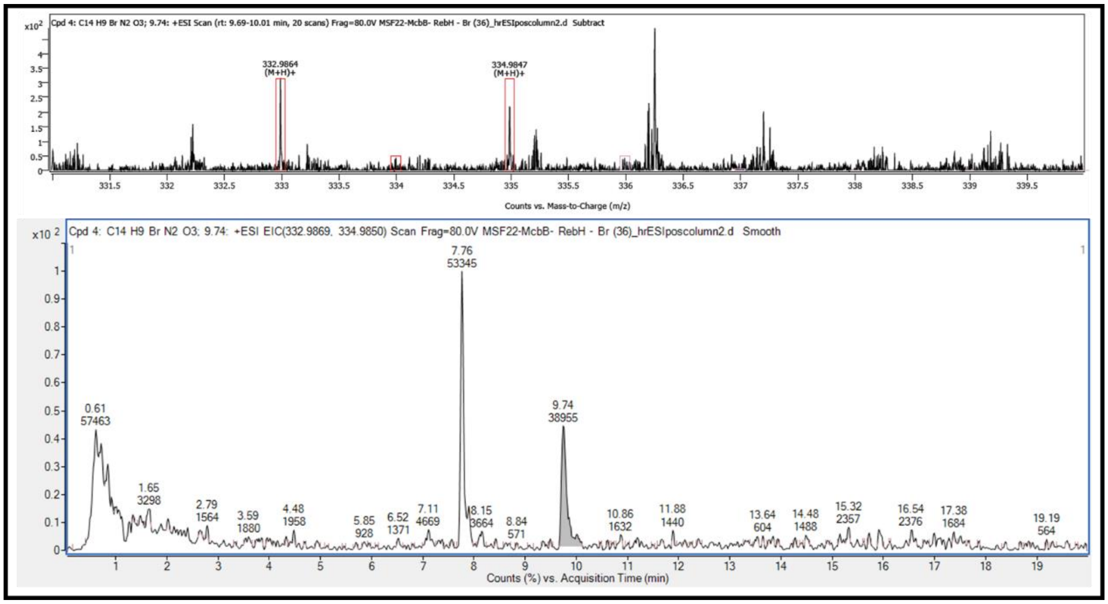

Supplementary Fig. 84. Analytical confirmation of *de novo* formation of 7-Br-1-acetyl-3-carboxyl β-carboline from glucose via coculture.
