# Supplementary material for: A modular and synthetic biosynthesis platform for de novo production of diverse halogenated tryptophan-derived molecules
Source: Nat Commun. 2024 Apr 12;15:3188. doi: 10.1038/s41467-024-47387-1 (PMC11015028; doi:10.1038/s41467-024-47387-1)
Supplement: Supplementary file 3 — Description of Additional Supplementary Files [file 41467_2024_47387_MOESM3_ESM.pdf]

Description of Additional Supplementary Files

File Name: Supplementary Data 1
Description: Estimated conversion of fed tryptophan or halo-tryptophan precursor in feeding assays. Conversion values calculated by dividing estimated feeding assay titers from Supplementary Table 2 by the quantity of precursor fed, multiplied by 100%.

File Name: Supplementary Data 2
Description: Estimated titers of downstream molecules produced by both the feeding assays and cocultures. As standards were not available for the vast majority of produced downstream molecules, titers from feeding assays (Fig. 5) were estimated based on consumed tryptophan or halogenated tryptophan precursor fed (on a mM basis), assuming all consumed precursor was converted to the desired downstream product. For the cocultures, estimated titers were calculated by multiplying the feeding assay estimated titers by the relative LCMS abundances of the appropriate downstream product (area from coculture divided by that of feeding assay). Direct comparisons of LCMS abundances were only carried out when values were from the same downstream product, to avoid the impact of differing ionization capacity impacting the results. Compounds not detected are denoted as N.D.

File Name: Supplementary Data 3
Description: Estimated yield of downstream products from cocultures. Estimated yields were calculated by dividing the estimated downstream product titers by the sum of the produced downstream product, and residual tryptophan and/or halogenated tryptophan precursors. The sum of the produced downstream product, and residual tryptophan and/or halogenated tryptophan precursors is taken to represent the maximum mM of product possible for each respective coculture, with a maximum achievable yield of 100%.

File Name: Supplementary Data 4
Description: Genes, promoters, and other genetic elements used in this study (generated via PCR or synthesized by IDT)
